# Identification of SET Domain-Containing Proteins in *Gossypium raimondii* and Their Response to High Temperature Stress

**DOI:** 10.1038/srep32729

**Published:** 2016-09-07

**Authors:** Yong Huang, Yijia Mo, Pengyun Chen, Xiaoling Yuan, Funing Meng, Shengwei Zhu, Zhi Liu

**Affiliations:** 1 College of Bioscience and Biotechnology, Hunan Agricultural University, Changsha 410128, P. R. China; 2 Key laboratory of Plant Molecular Physiology, Institute of Botany, Chinese Academy of Sciences, Beijing 100093, P. R. China

## Abstract

SET (Su(var), E(z), and Trithorax) domain-containing proteins play an important role in plant development and stress responses through modifying lysine methylation status of histone. *Gossypium raimondii* may be the putative contributor of the D-subgenome of economical crops allotetraploid *G. hirsutum* and *G. barbadense* and therefore can potentially provide resistance genes. In this study, we identified 52 SET domain-containing genes from *G. raimondii* genome. Based on conserved sequences, these genes are grouped into seven classes and are predicted to catalyze the methylation of different substrates: *GrKMT1* for H3K9me, *GrKMT2* and *GrKMT7* for H3K4me, *GrKMT3* for H3K36me, *GrKMT6* for H3K27me, but *GrRBCMT* and *GrS-ET* for nonhistones substrate-specific methylation. Seven pairs of *GrKMT* and *GrRBCMT* homologous genes are found to be duplicated, possibly one originating from tandem duplication and five from a large scale or whole genome duplication event. The gene structure, domain organization and expression patterns analyses suggest that these genes’ functions are diversified. A few of *GrKMT*s and *GrRBCMT*s, especially for *GrKMT1A;1a*, *GrKMT3;3* and *GrKMT6B;1* were affected by high temperature (HT) stress, demonstrating dramatically changed expression patterns. The characterization of SET domain-containing genes in *G. raimondii* provides useful clues for further revealing epigenetic regulation under HT and function diversification during evolution.

Epigenetics is the study of inheritable genetic changes without a change in DNA sequence^1^. Molecular mechanisms of epigenetic regulation mainly consist of DNA methylation, chromatin/histone modifications and small non-coding RNAs etc^2^. Being one of most important epigenetic modifications, histone modification occurs primarily on lysines and arginines, including phosphorylation, ubiquitination, acetylation, methylation and others^3^. Among these covalent modifications, histone methylation and demethylation are catalyzed by Histone Lysine Methyltransferases (KMTs) and Histone Lysine Demethylases (KDMs), respectively.

KMTs commonly include an evolutionarily conserved SET (Su(var), E(z), and Trithorax) domain, which carries enzyme catalytic activity for catalyzing mono-, di-, or tri- methylation on lysine^4^. The SET domain typically constitutes a knot-like structure formed by about 130–150 amino acids, which contributes to enzymatic activity of lysine methylation^5^. To date, a number of SET domain-containing proteins have been discovered and analyzed in the released genomic sequences of model plants. Baumbusch *et al*. early reported that *Arabidopsis thaliana* had at least 29 active genes encoding SET domain-containing proteins^6^, and Springer *et al*. found 32 *Arabidopsis* SET proteins, which were divided into five classes and 19 orthology groups^7^, and then Ng *et al*. detected 7 classes, 46 *Arabidopsis* SET proteins^8^. Based on different substrate specificities, Huang *et al*. have recently proposed a new and rational nomenclature, in which plant SET domain-containing proteins were grouped into six distinct classes: KMT1 for H3K9, KMT2 for H3K4, KMT3 for H3K36, KMT6 for H3K27 and KMT7 for H3K4, while S-ETs contain an interrupted SET domain and are likely involved in the methylation of nonhistone proteins^9^. Besides the above major KMT classes, rubisco methyltransferase (RBCMT) family proteins are also identified as specific methyltransferases for nonhistone substrate in plants and consist of large subunit Rubisco methyltransferase (LSMT) and small subunit Rubisco methyltransferase (SSMT)^8^,^10^.

It was shown that SET domain-containing proteins regulated plant developmental processes such as floral organogenesis, seed development^11^ and plant senescence^12^. More recent studies demonstrated that SET domain-containing proteins were also involved in plant defense in response to different environmental stresses. In euchromatin, methylation of histone H3K4, H3K36 and H3K27me3 were shown to be associated with gene regulations including transcriptional activation and gene silencing^13^. For example, histone modifications (e.g. enrichment in H3K4me3) on the H3 N-tail activated drought stress-responsive genes^14^. By establishing the trimethylation pattern of H3K4me3 residues of the nucleosomes, *ATX1/SDG27* (*Arabidopsis Homolog of Trithorax*) regulates the SA/JA signaling pathway for plant defense against bacterial pathogens by activating the expression of the *WRKY70*, which was a critical transcription factor^15^. By regulating H3K36 methylation of histone proteins in JA (jasmonic acid) and/or ethylene^13^ and brassinosteroids signaling pathway, *Arabidopsis* SDG8 (SET Domain Group 8) was shown to play a critical role against fungal pathogens *Alternaria brassicicola* and *Botrytis cinerea*^16^.

Furthermore, low or high temperature stress is one of serious environmental stresses affecting plant development. When *Arabidopsis* plants were exposed to cold temperature, H3K27me3 was significantly reduced in the area of chromatin containing COR15A (Cold-regulated15A) and ATGOLS3 (Galactinol Synthase 3) 17, which are cold stress response genes. In recent years, high temperature (HT) stress has gradually become a serious threat to crop production as global warming is getting worse. Cotton (*Gossypium* spp) is one of important crops in many parts of the world and is sensitive to HT stress^18^, which severely affects pollen formation, pollen germination, subsequent fertilization, and ovule longevity, leading to boll shedding and the significant reduction of cotton yield^19^. Therefore there is a great urge to screen and identify the potential genes conferring resistance to HT stress in molecular breeding of cotton. However, our understanding of mechanisms of resistance to HT in cotton is limited. The progenitor of *Gossypium raimondii* (*G. raimondii*) may be the putative contributor of the D-subgenome of *Gossypium hirsutum* (*G. hirsutum*) and *Gossypium barbadense* (*G. barbadense*) and, more importantly, provides lots of resistant genes^20^. In this study, we identified SET domain-containing proteins from whole genome of *G. raimondii*. Based on the analysis of phylogenetic tree, classification, gene structure and domain organization, gene expression profiling and response to HT stress, these results suggested the possible roles of different *GrKMT* and *GrRBCMT* genes in the development of *G. raimondii* and in response to HT. This study of SET domain-containing protein in *G. raimondii* have expanded understanding of the mechanism of epigenetic regulation in cotton and potentially provide some clues for discovering new resistant genes to HT stress in cotton molecular breeding.

## Results

### Identification of 52 SET domain-containing proteins in G. raimondii

**Figure 1:**
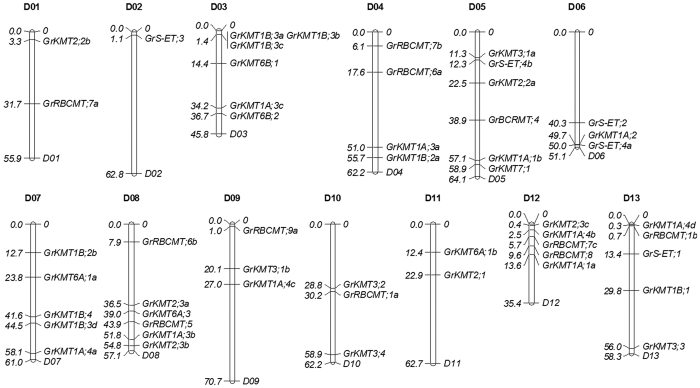
Chromosomal distribution of *GrKMT* and *GrRBCMT* genes. 52 *Gr*KTTs and *GrRBCMT*s have been mapped on chromosomes D01-D13 except *GrRBCMT;9b* (Gorai.N022300). The chromosome map was constructed using the Mapchart 2.2 program. The scale on the chromosome represents megabases (Mb) and the chromosome number is indicated at the top of each chromosome.

To obtain all the member of SET domain-containing proteins in *G. Raimondii*, BLASTP analysis was performed using the sequence of SET domains of known *Arabidopsis* SET domain-containing protein against *G. Raimondii* genome Database. Fifty-two SET domain-containing members were identified in *G. raimondii* (Fig. 1, Supplementary Table S2, S3). Based on the KMT nomenclature and relationship to *Arabidopsis* homologs, each sequence was assigned to different KMT families (GrKMTs)^9^, and the candidate proteins similar to Rubisco methyltransferase family proteins were named as GrRBCMTs^8^.

In total, 51 *GrKMT*s and *GrRBCMT*s have been mapped on chromosomes D01-D13 except for *GrRBCMT;9b* (Gorai.N022300) that is still on a scaffold (Fig. 1, Supplementary Table S2). In Chromosome D03, D05 and D08, there are at least six *GrKMT*s or *GrRBCMT*s; in chromosome D07, D12 and D13, there are less than six but more than one *GrKMT*s or *GrRBCMT*s, while chromosome D02 with 62.8Mb in length has only one member, *GrS-ET;3*.

**Figure 2:**
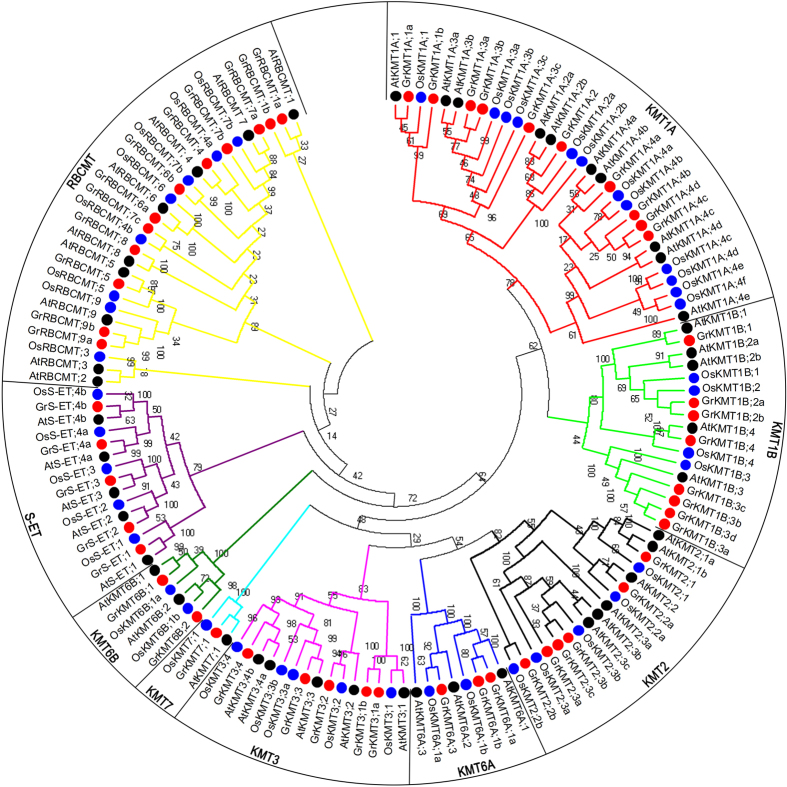
Phylogenetic tree of KMT and RBCMT proteins. This tree includes 52 SET domain-containing proteins from *G. raimondii*, 45 from *A. thaliana* and 44 from *O. sativa*. The 141 SET domain-containing proteins could be grouped into seven distinct classes, Class KMT1, KMT2, KMT3, KMT6, KMT7, S-ET and RBCMTs. KMT and RBCMT proteins sequences were aligned using Clustal W, and the phylogenetic tree analysis was performed using MEGA 6.0. The tree was constructed with the following settings: Tree Inference as Neighbor-Joining; Include Sites as Partial deletion option for total sequence analyses; Substitution Model: p-distance; and Bootstrap test of 1000 replicates for internal branch reliability. Gr, *G. raimondii*; At, *A. thaliana*; Os, *O. sativa*.

**Figure 3:**
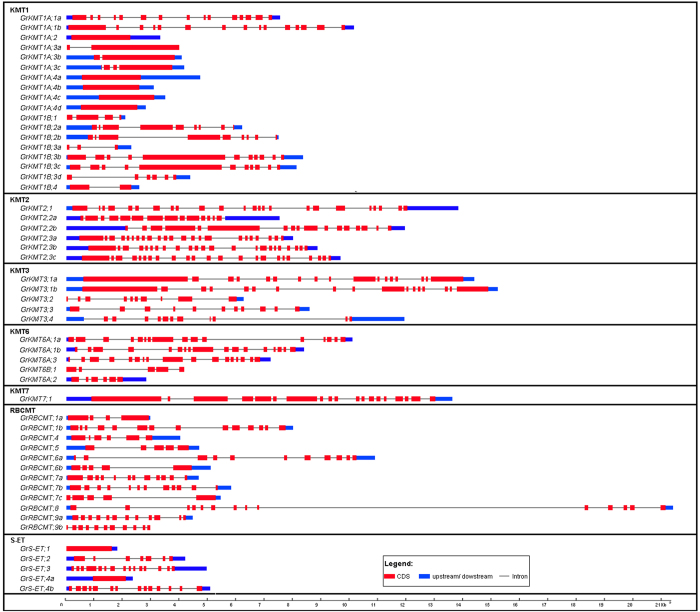
Gene structure of *GrKMT*s and *GrRBCMT*s. The gene structure of *GrKMT*s and *GrRBCMT*s were constructed by Gene Structure Display Server (http://gsds.cbi.pku.edu.cn/).

According to the canonical criteria^21^,^22^, six pairs genes, *GrKMT1B;2a/2b, GrKMT1B;3a/3d, GrKMT1B;3b/3c GrKMT2;3b/3c, GrKMT6A;1a/1b, GrRBCMT;9a/9b* were diploid and *GrKMT1A;4b/4c/4d* were triploid. Most of duplicated genes are in class GrKMT1. Among them, *GrKMT1B;3b/3c* may be tandemly duplicated and others are more likely due to large scale or whole genome duplication except that *GrRBCMT;9a/9b* cannot be confirmed (Supplementary Table S4). In general, homologous genes are clustered together in the phylogenic tree and the duplicated genes share similar exon-intron structures, higher coverage percentage of full-length-CDS sequence and higher similarity of encoding amino acid (Figs 2 and 3; Supplementary Table S4).

### Phylogenetic analysis of SET domain-containing proteins

To analyze the characteristics of 52 SET domain-containing protein sequences in *G. raimondii*, 45 SET domain-containing protein sequences from *A. thaliana* and 44 SET domain-containing protein sequences from *O. sativa* (Supplementary Tables S2 and S3) were also extracted for the phylogenetic analysis. Based on canonical KMT proteins, the above 141 SET domain-containing proteins could be grouped into seven distinct classes (Fig. 2), class KMT1, KMT2, KMT3, KMT6, KMT7 and S-ET^9^, and class RBCMT once named SETD^23^. KMT1 exhibits H3K9 substrate specificities activity, KMT2/KMT7 for H3K4, KMT3 for H3K36 and KMT6 for H3K27. RBCMT possesses H3K4 and H3K36 methyltransferase activity in animals, but non-histone target specific proteins in plant^8^,^10^. The function of S-ET is still unclear. Furthermore, there are 18 members (10 in KMT1A and 8 in KMT1B) in Class KMT1 as the largest family of KMTs in the SET domain-containing proteins, following by 12 members in class RBCMT, while there is only one member in class KMT7 from each examined species.

### Gene structure and domain organization of *GrKMTs* and *GrRBCMTs*

To understand the evolutionary origin and putative functional diversification, the gene structure of *GrKMTs* and *GrRBCMTs* was analyzed in their constitution of introns/exons. Our results showed that the number of introns/exons was various among different *GrKMTs* and *GrRBCMTs*. Most of *GrKMT* and *GrRBCMT* genes possess multiple exons, except *GrKMT1A;2*, *GrKMT1A;4a/4b/4c/4d* and *GrS-ET;1/4a* with only one (Fig. 3, Supplementary Table S2). Class *GrKMT1A* consists of relatively consistent exon number except *GrKMT1A;1a/1b* with fifteen, *GrKMT1A;3a/3b* with two and *GrKMT1A;3c* with four. Altogether, the number of exons in each class genes is greatly variable, and most of Class GrKMT2 genes contain the largest number of exons.

**Figure 4:**
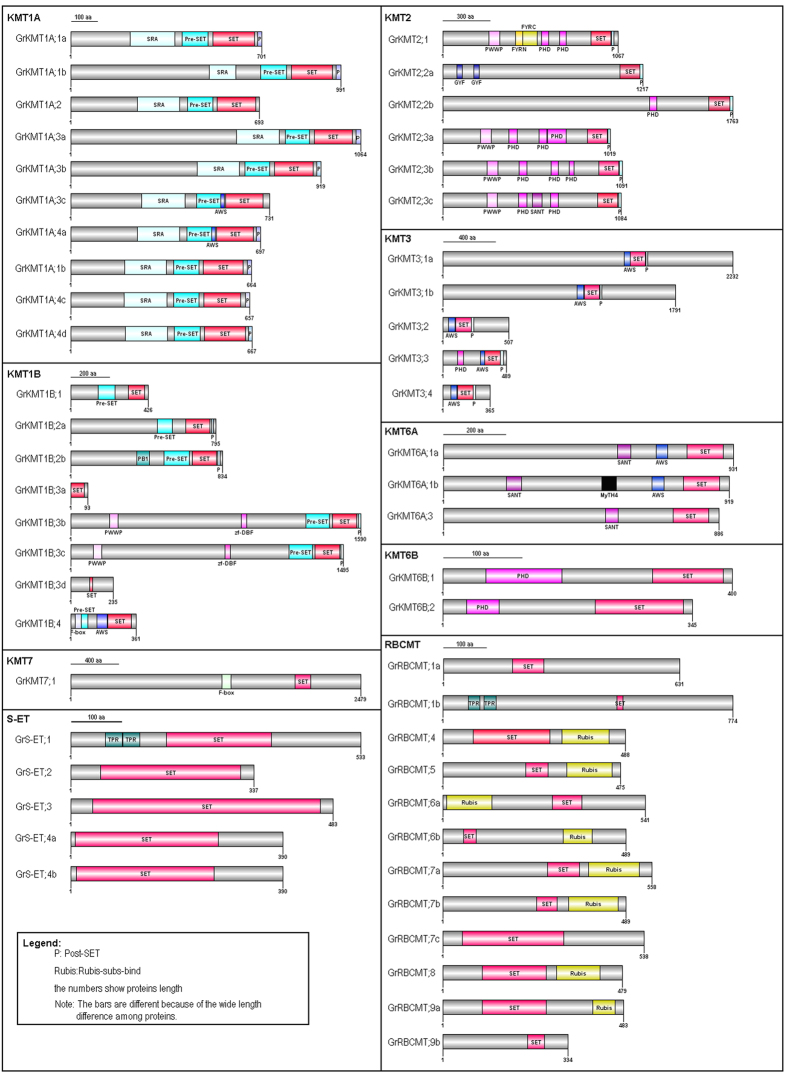
Domain organization of GrKMT and GrRBCMT proteins. Domain organization of SET domain-containing proteins in *G. raimondii* were detected by SMART and NCBI (http://www.ncbi.nlm.nih.gov/Structure/cdd/wrpsb.cgi), and the low-complexity filter was turned off, and the Expect Value was set at 10. The site information of domains was subjected to Dog2.0 to construct the proteins organization sketch map.

To explore the gene structure, the sequences of full-length GrKMTs and GrRBCMTs were deduced and their domain organization was examined. In GrKMTs, SET domain always locates at the carboxyl terminal of proteins, except Class S-ET and RBCMT. Among the same KMT class, the predicted GrKMTs and GrRBCMTs always share relatively conserved domain organization (Fig. 4, Supplementary Table S3).

Based on the analysis of protein motifs in Class GrKMT1 proteins, they has mostly associated with SET motif and SRA (SET- and RING-associated) motif facilitating DNA accession and the binding of target genes at the catalytic center^24^. In Class GrKMT1 proteins, they also possess SET domain boundary domains, Pre-SET and Post-SET domains, which are usually present in other plant species^25^. Pre-SET is involved in maintaining structural stability and post-SET forms a part of the active site lysine channel^26^. Besides these typical domains, GrKMT1A;3c/4a also include additional AWS domain (associated with SET domain), which is highly flexible and involved in methylation of lysine residues in histones and other proteins^27^. Class KMT1B proteins also possess SET and Pre-SET domains except GrKMT1B;3a/3d, which are much shorter than the others and only has SET domain (Fig. 4, Supplementary Table S3). Other GrKMT proteins have some additional domain(s): Post-SET domain in GrKMT1B;2a; PB1 (a protein-protein interaction module) and Post-SET domain in GrKMT1B;2b; PWWP (Pro-Trp-Trp-Pro) that is a DNA binding domain and protein-protein interaction domain^28^, Zf-DBF that is predicted to bind to metal ions and Post-SET in GrKMT1B;3b/3c; F-box which is required for gene silence by means of interaction with core components^29^ and AWS domain in GrKMT1B;4. Class GrKMT2 proteins contain SET, post-SET and PHD (plant homeodomain) domain except GrKMT2;2a without PHD domain (Fig. 4, Supplementary Table S3). PHD domain has multiple functions by controlling gene expression as an epigenome reader through binding to nucleosomes^30^. GrKMT2;1 has additional PWWP and FYRN-FYRC (DAST, Domain associated with SET in Trithorax) domains as chromatin-associated proteins involved in histone modifications and a signature feature for the trithorax gene family respectively^31^. GrKMT2;2a has two GYF (glycine-tyrosine-phenylalanine) domains, which bind to lots of different proline-rich sequences (PRS)^32^. GrKMT2;3c has an additional SANT (SWI3-ADA2-N-CoR-TFIIIB) domain, which is mainly found in KMT6A. In Class GrKMT3, the SET-domain containing GrKMT3 proteins are more conserved in domain organization and all possess AWS, SET and post-SET domains except GrKMT3;3 with an additional PHD domain (Fig. 4, Supplementary Table S3). It is surprising that SET domain in GrKMT3;2 and GrKMT3;4 are located at the N-terminal or in the middle of the protein sequence, respectively. In Class GrKMT6, the SET-domain containing GrKMT6 proteins are also conserved in domain organization and proteins length (Fig. 4, Supplementary Table S3). GrKMT6A proteins possess SANT, AWS and SET domain except GrKMT6A;1b with an additional MyTH4 (Myosin Tail Homology) domain that can bind to microtubules in combination with FERM proteins (band 4.1, ezrin, radixin, moesin)^33^. SANT is a putative DNA-binding domain in many transcriptional regulatory proteins and is essential for histone acetyltransferase activity^34^. GrKMT6B proteins only include PHD and SET domain. In the class GrKMT7 proteins, there is only one member, GrKMT7;1, which is the longest GrKMT protein analyzed with F-box and SET domain.

S-ET proteins commonly have an interrupted SET domain and may be involved in H3K36me3 in human, but their functions are unknown in plant species^8^. GrS-ET family has 5 members with an interrupted SET domain with 194–264 aa in length. Compared to S-ET proteins in other plant species, they only contain a full interrupted SET domain except GrS-ET;1, which has two additional tandem TPR domains (tetratricopeptide repeat) acting as interaction scaffolds for the formation of multi-protein complexes^35^. GrRBCMT (plant SETD orthology groups) proteins include SET and Rubis-subs-bind domains except that GrRBCMT;1a/7c/9b only contains a SET domain and GrRBCMT;1b has TPR and SET domains (Fig. 4, Supplementary Table S3).

### Tissue and organ expression of *GrKMTs* and *GrRBCMTs*

To explore the possible physiological functions of SET domain-containing proteins in *G. raimondii*, we designed gene-specific real-time quantitative RT-PCR primers (Supplementary Table S1) for detecting the expression patterns of 52 *GrKMT* and *GrRBCMT* genes in different tissues and organs, including root, stem, leaf, petal, anther, and ovary.

**Figure 5:**
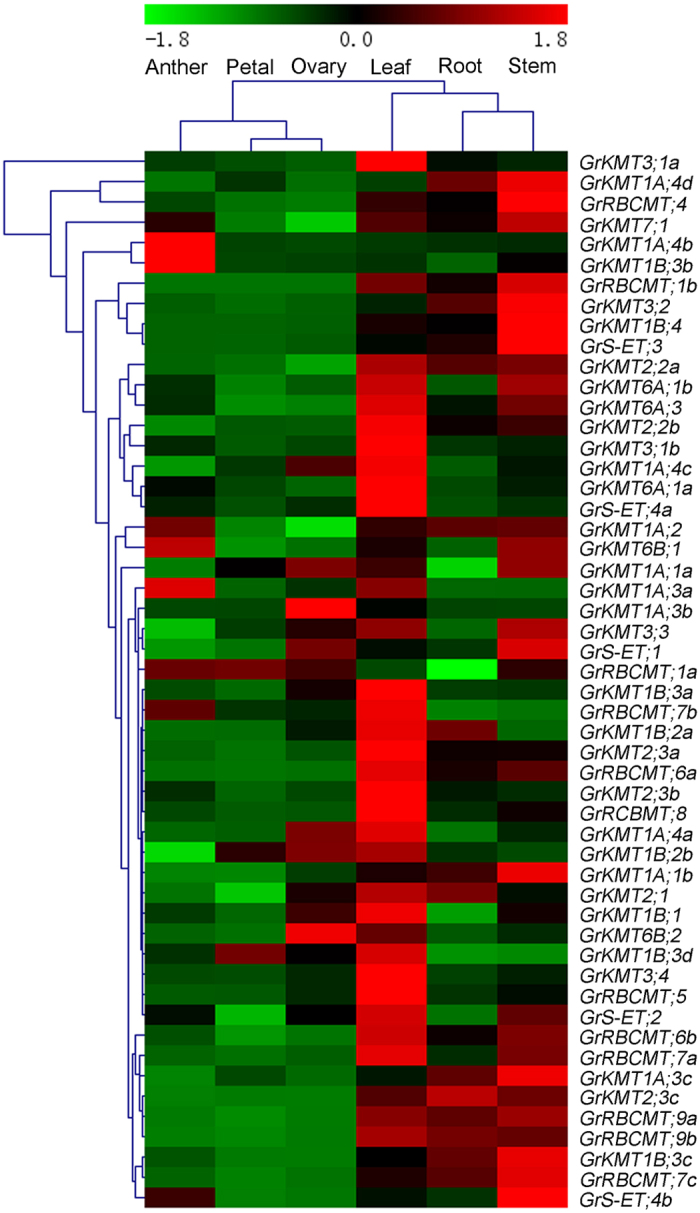
Tissue and organ expression of *GrKMT*s and *GrRBCMT*s. A heatmap for gene expression patterns was generated with the software MultiExperiment Viewer (MeV). The expression patters of *GrKMT* and *GrRBCMT* genes are obviously tissue and organ special. Most of genes low express in petal, and high in leaf. Duplicated genes higher express in vegetative organs, and except that GrKMT1A;4b and GrKMT1B;3b strongly express in reproductive organs.

As indicated in Fig. 5, the SET domain-containing genes from *G. raimondii* showed diverse expression patterns in different tissues and organs. First, some genes from the same class differentially expressed in the six tissues and organs tested while other genes from different classes could also show similar expression patterns in different tissues and organs, indicating that dramatically functional divergence of *GrKMT* and *GrRBCMT* genes during plant development. Second, the expression patterns of *GrKMT*s and *GrRBCMT*s are obviously tissue and organ specific at a very low level of expression in reproductive organs and relatively high expression level in vegetative tissues. Furthermore, the majority of genes from different *GrKMT* and *GrRBCMT* classes were highly expressed in leaf and stem, indicating that they may play important roles in the development of leaf and stem (Fig. 5). In addition, *GrKMT1A;4b* and *GrKMT1;3b*, *GrKMT1A;3b* and *GrS-ET;1* were highly expressed in anther and ovary, respectively, implying their specific functions in the corresponding tissues.

Seven pairs of duplicated genes from the *GrKMTs* and *GrRBCMTs* were also highly expressed in vegetative organs, leaf and stem, but with a low expression level in reproductive organs, except that *GrKMT1A; 4b* and *GrKMT1B;3b* highly expressed in anther (Fig. 5, Supplementary Figure S1). *GrKMT6A;1a*/*1b* and *GrRBCMT;9a/9b* showed similar expression patterns, while other duplicated genes differentially expressed in the six tissues and organs tested, suggesting that the expression patterns and functions of these genes are diverged during the evolution of gene duplication.

### Expression profiles of *GrKMT*s and *GrRBCMT*s in response to high temperature stress

**Figure 6:**
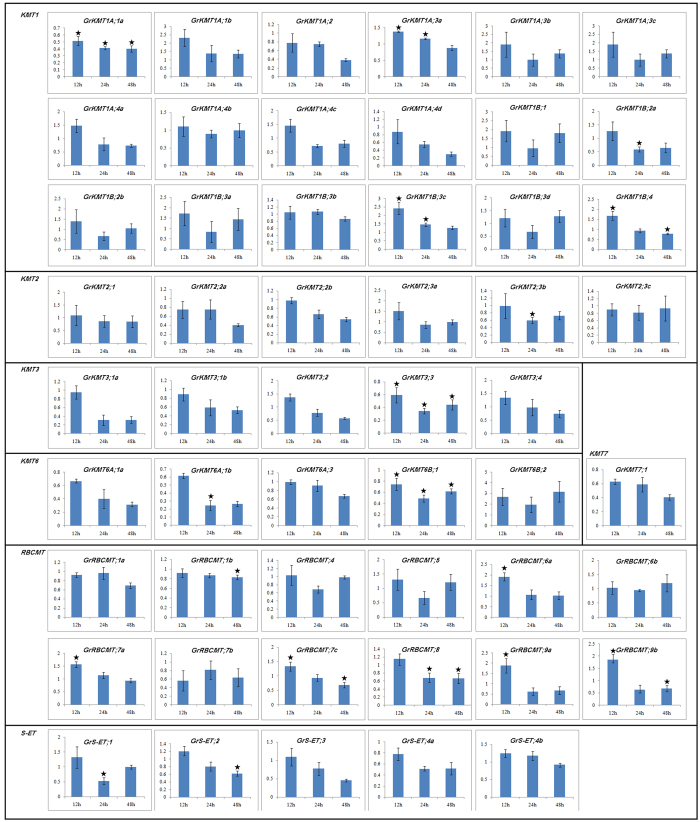
Expression of *GrKMT*s and *GrRBCMT*s in response to high temperature. Many *GrKMT* and *GrRBCMT* genes are involved in high temperature response. Among them GrKMT1A;1a with H3K9 activity, GrKMT3;3 with H3K36 activity and GrKMT6B;1 with H3K27 activity maintain lower expression level at the process of the high temperature treatments. The error bars depict SD, and the asterisk shows the corresponding gene significantly up- or down-regulated by Student′s *t* test between the treatment and the control (P < 0.05).

Molecular mechanism of epigenetic regulation is poorly understood in response to HT stress in cotton. In our current study, most of *GrKMTs* and *GrRBCMTs* were strongly expressed in leaf (Fig. 5). To better understand the roles of the SET domain-containing proteins in response to HT stress, after treatment at 38 °C, the expression profiles of *GrKMT* and *GrRBCMT* genes in leaves of seedlings were examined by real-time quantitative RT-PCR (Fig. 6), showing that the expression level of all the *GrKMTs* and *GrRBCMTs* genes were more or less affected by HT stress, but the change of their expression patterns were diverse. The expression of most of genes was shown to be decreased under HT conditions; only *GrKMT1A;1b*, *GrKMT1B;3c*, and *GrKMT6B;2* were up-regulated and reached a peak at 12h after HT treatment. Of these examined genes, *GrKMT1A;1a*, *GrKMT3;3* and *GrKMT6B;1* were dramatically down-regulated after the HT treatment.

All in all, upon exposure to HT, the transcript levels of seven members of *GrRBCMT* (*GrRBCMT;1b/6a/7a/7c/8/9a/9b*), five of *GrKMT1* (*GrKMT1A;1a/3a*, *GrKMT1B;2a/3c/4*), two of *GrKMT6* (*GrKMT6A;1b/GrKMT6B;1*), two of *GrS-ET* (*GrS-ET;1/GrS-ET;2*), *GrKMT2;3b* and *GrKMT3;3*, were significantly different from that of control at least at one time point (P < 0.05, Fig. 6).

## Discussion

### Classification and putative functions of *GrKMTs* and *GrRBCMTs* genes were predicted

Allotetraploid cotton *G. hirsutum* and *G. barbadense* are important economical crops and model plants for polyploids evolution studies. Genomes of *G. hirsutum* and *G. barbadense* may derived from allopolyploidization of D-subgenome (*G. raimondii* Ulbrich) and A-subgenome (*G. herbaceum* L)^36^. D-subgenome does not produce any spinnable fiber, but provides many fiber genes after merging with A genome^37^, contributing to stress tolerance during allotetraploid cotton domestication^20^. Nowadays, it is known that *G. ramondii* genome encodes 1004 resistant genes to *Verticillium wilt*^38^, 35 auxin response factors (*ARFs*)^22^ and 205 putative R2R3-MYB genes^39^ and so on.

In previous studies, it was shown that histone modifications played important roles in plant development^11^ and response to biotic and abiotic stress^40^. KMTs and KDMs tightly regulated the methylation status of lysine residues within histones^41^. Furthermore the status of histone lysine methylation links to the regulation of the expression of targeted genes. For example, H3K9 and H3K27 methylation is associated with gene silencing, whereas H3K4 and H3K36 methylation lead to gene activation^42^. It was known that histone lysine methyltransferases shared a highly conserved SET domain except Dot1 for H3K79 methylation^43^. SET domain-containing proteins could be divided into seven classes, based on their specificity for substrates^9^. In this study, we revealed that *G. ramondii* possessed 52 SET domain-containing proteins, which could be grouped to six KMT and one RBCMT classes (Fig. 2) including KMT1 (18), KMT2 (6), KMT3 (5), KMT6 (5), KMT7 (1), S-ET (5) and RBCMT (12). In SET domain-containing proteins of *G. ramondii* belonging to the first six classes, it was found that their domain organization was largely similar to the counterparts in *Arabidopsis* and *Brassica rapa*^9^. Besides SET domain and several associated domains, our results also showed that GrKMT1A, GrKMT2, GrKMT3, GrKMT6A, GrKMT6B and GrKMT7 proteins also contained SRA domain, PHD and PWWP domain, AWS domain, SANT domain, PHD domain, F-box domain respectively. Moreover, the domain organization of KMT1B is much more complex (see Fig. 4, Supplementary Table S3). Among these domain or motifs in the KMT proteins from *G. ramondii*, the intact SET domain in GrKMT proteins could insure the necessary methyltransferases activity, and other domains could provide more auxiliary roles.

*G. ramondii* was also found to have longer and interrupted SET domain in GrS-ET and Rubis-subs-bind domains in GrRBCMT. Previous report indicated that class S-ET proteins might lack methyltransferase activity. In animals, SETD3 containing SET and Rubis-subs-bind domains was found to have a H3K4/K36 methyltransferase activity^44^. Even though RBCMT proteins were identified in land plants and green algae, but their biological functions were still uncertain^23^. Ng *et al*. suggested that RBCMT class proteins had the weaker KMT activity from their similar and longer SET domain than that of canonical KMTs, but maintained the activity of non-histone substrate-specific methylation^8^. Ma *et al*. also found that LSMTs could trimethylate Rubisco in *Fabaceae*, *Cucurbitaceae* and *Rosaceae*, in addition to chloroplastic aldolases, which were only aldolases in most other plants^10^. However, possible biological functions of both GrS-ET and GrRBCMT proteins are still unclear in our current study.

Based on previous studies in SET domain-containing proteins in several plant species, we could predict the substrate specificities of different SET domain-containing proteins in *G. ramondii*: KMT1 for H3K9, KMT2 for H3K4, KMT3 for H3K36, KMT6 for H3K27 and KMT7 for H3K4 and also RBCMT for putative non-histone substrates.

### *GrKMT*s and *GrRBCMT*s genes were involved in HT response

Genetic and epigenetic regulations of genes were demonstrated to play key roles in plant response to environmental high or low temperature. It was documented that histone methylation was the major epigenetic regulatory mechanism in response to biotic or abiotic stresses^45^. KMT proteins regulated the activity of target genes by methylating histone H3, such as, H3K4me and H3K36me associating with transcriptional activation, whereas H3K9me and H3K27me leading to gene silence^13^. It was also documented that drought stress^14^, pathogens^46^ and chilling^17^ response gene could be regulated by histone methylation. However, the roles of KMT proteins in HT stress were shown to be controversial at best: H3K4me1 of *Chlamydomonas reinhardtii* and H3K9me2 of *OsFIE1* were sensitive to HT, while H3K9me2, H3K27me1/me2/me3 and H3K4me3 in *Arabidopsis* were not; a transcriptome analysis indicated that differential gene expressions between normal and high temperature conditions were directly related to epigenetic modifications, carbohydrate metabolism, and plant hormone signaling^47^. Our current results showed that many GrKMTs with histone methylation activity were involved in HT response (Fig. 6). Upon exposure to HT, up- or down- regulation of these genes might affect the status of methylation and further regulate the activity of target genes in response to HT. *GrKMT1A;1a* with H3K9 activity, *GrKMT3;3* with H3K36 activity and *GrKMT6B;1* with H3K27 activity maintain lower expression level during the HT response. *AtKMT1A;1* (*SDG33*/*SUVH4*), homologous gene to *GrKMT1A;1a* is involved in host defense system by regulating target genes H3K9me^48^. *KMT6B;1*(*SDG1*/*CLF*) is one of core components of PRC2 and mainly contributes to the H3K27 activity^49^, whose increase at stress gene loci will repress heat shock response (HSR)^50^. However, the function of *AtKMT3;3* (*SDG4*/*ASHR*/*SET4*) in resistance response is unknown. Therefore, we may infer that the lower level of H3K9 and H3K27 methylation will activate more target genes that are involved in HT responses, and the change of H3K27 activity is completely consistent with Kwon *et al*.^17^.

Plant reproductive tissues or organs contribute to seed set yield and are the most vulnerable parts to HT stress^51^. Our study predicted that *GrKMT1A;4b*, *GrKMT1B;3b*, *GrKMT1A;3a* and *GrKMT1A;3b* were presumed to be involved in H3K9me. These genes were found to be strongly expressed in anther or ovary, but at a low expression level in the vegetative organs. Among the genes in leaves dramatically regulated by HT stress, *GrKMT1A;1a*, *GrKMT1A;2*, *GrKMT3;3*, *GrKMT6B;1*, and *GrKMT6B;2* highly expressed in anther and ovary (Figs 5 and 6), suggesting that if the roles of *GrKMT*s and *GrRBCMT*s were further investigated in reproductive tissues or organs, it would be able to mine novel resistant genes and provide new understanding for plant HT stress response.

### Evolution of *GrKMT*s and *GrRBCMT*s impacts differentially on their functions

It has been our main interest how the evolution of duplicated genes affects their biological functions, since gene duplication has played a vital role in the evolution of new gene functions and is one of the primary driving forces in the evolution of genomes and genetic systems^52^. Gene families may evolve primarily through tandem duplication and polyploidy or large-scale segmental duplications^52^. *Arabidopsis* genome has undergone about two rounds of duplications before *Arabidopsis*/*Brassica rapa* split and after the monocot/dicot divergence^53^. The outcomes of duplicated genes include nonfunctionalization, neofunctionalization and subfunctionalization^54^. The nonfunctionalization of one copy is the most likely fate due to deleterious mutation, functionally redundant and dosage constraints^54^. *G. ramondii* undergone independent whole-genome duplication event approximately 13.3 to 20.0 million years ago, and shared one paleohexaploidization event with eudicots, but has a higher gene number and lower mean gene density compared with *Arabidopsis*^36^, meaning many genes were lost after duplication. We identified 46 *KMT*s and *RBCMT*s in *Arabidopsis* (2n = 10) and only 52 members in *G. ramondii* (2n = 26). Based on the canonical criteria^21^,^22^, seven pairs of *GrKMT* or *GrRBCMT* genes were created by the duplication of homologous genes. *GrKMT1B;2a*/*2b*, *GrKMT1B;3a/3d*, *GrKMT2;3b*/*3c*, *GrKMT6A;1a*/*1b*, *GrRBCMT;9a*/*9b*, *GrKMT1A;4b*/*4c*/*4d* might be due to ancient large-scale duplication event, while *GrKMT1B;3b*/*3c* may formed by tandem duplication (Supplementary Table S4). Even though *GrKMT1B;3a* was also shown to meet the parameters of duplicated genes for *GrKMT1B;3b/3c/3d* in NCBI, they were not considered as duplicated genes since *GrKMT1B;3d* is much shorter than *GrKMT1B;3b/3c* (Fig. 4; Supplementary Table S4). *GrRBCMT;9a*/*9b* as duplicated genes also could not be confirmed, because *GrRBCMT;9b* (Gorai. N022300) still not be mapped on any chromosome (Fig. 1).

Duplicated genes can generally be grouped into one clade of phylogenetic tree (Fig. 2); most of these genes exist in sister pairs or triplets and have similar gene structure with possible similar functions, whereas others are divergent in the distribution of introns/exons, suggesting the possibility of functional diversification^22^. We found that the gene structure was conserved in most of GrKMT genes, except GrKMT6A;1a/1b and GrRBCMT;9a/9b with one exon difference; domain organization of GrKMT1A;4b/4c/4d and GrKMT2;3b/3c were conserved, but GrKMT1B;2a/2b, GrKMT6A;1a/1b and GrRBCMT;9a/9b are divergent (Figs 3 and 4, Supplementary Table S3); only sisters genes of GrKMT6A;1a/1b and GrRBCMT;9a/9b showed similar expression patterns in different tissues and organs. For example, GrKMT1;3b/3c have same gene structure, domain organization, but GrKMT1;3b only highly expresses in anther, and is not involved in HT stress, and GrKMT1; 3c strongly expresses in root, stem and leaf and is sensitive to HT stress (Figs 3, 4, 5, 6; Supplementary Figures S1 and S2). Most duplicated genes also showed similar expression pattern in leaf except GrKMT1A;4b/4c/4d (Supplementary Figures S1 and S2), suggesting that some duplicated genes undergone functional differentiation but others not.

## Methods

### Identification of SET domain-containing proteins and construction of chromosome map

Sequences of SET domain-containing proteins from *Arabidopsis thaliana* were retrieved from the official website (https://www.arabidopsis.org/Blast/index.jsp). The sequences of SET domain of these sequences were used as queries to search *G. raimondii* homologs (http://www.phytozome.net, version 10.3) using the BLASTp. The sequence of SET domain-containing proteins of rice was extracted from Huang *et al*.^9^ and web http://www.phytozome.net (version 10.3). All the sequences were re-confirmed in SMART database (http://smart.embl-heidelberg.de/). The gene loci information of *G. raimondii* was used to generate the chromosome maps by the Mapchart 2.2 program^55^.

When candidate genes was found to be both >70% coverage of shorter full-length-CDS sequence and >70% identical in the sequence of their encoding amino acids, they were regarded as duplicated genes^21^. When the duplicated genes were located within 100 kb and were separated by ten or fewer non-homologues, they were defined as tandem duplicated genes^22^. The coverage of full-length-CDS sequence and the similarity of amino acid sequences were detected by Blastn/Blastp in NCBI.

### Analysis of gene structure, domain organization and phylogenetic tree

The gene structure was reconstructed using Gene Structure Display Server (http://gsds.cbi.pku.edu.cn/). Domain organization was confirmed by SMART and NCBI (http://www.ncbi.nlm.nih.gov/Structure/cdd/wrpsb.cgi), and the low-complexity filter was turned off, and the Expect Value was set at 10. Then the site information of domains was subjected to Dog2.0 to construct the proteins organization sketch map^56^.

Multiple sequence alignments of SET domains were carried out by the Clustal W program^57^ and the resultant file was subjected to phylogenic analysis using the MEGA 6.0 program^58^. Based on the full-length protein sequences, the phylogenetic trees were constructed using Neighbor-Joining methods with Partial deletion and p-distance Method, Bootstrap test of 1000 replicates for internal branch reliability.

### Plant material and high temperature treatment

*G. raimondii* seedlings were grown in greenhouse at 28 °C under a 10 h day/14 h night cycle. 5-week-old seedlings with 5–6 true leaves were placed in a growth chamber at high temperature condition (38 °C; 28 °C as a mock) for 12, 24, and 48 h. The leaves were harvested at the appropriate time points as indicated (triplicate samples were collected at each time point) for detecting genes expression in response to HT. The roots, stems and leaves were collected from plants at the stage of 5–6 true leaves and the petals, anther and ovary were sampled on the day of flowering for gene expression analysis of tissue/organ. The materials were quick frozen in liquid nitrogen and stored at −70 °C for further analysis.

### RNA extraction and real-time quantitative RT-PCR

Total RNA was extracted from the materials mentioned above using TRIzol reagent kit (Invitrogen, Carlsbad, CA, US) according to the manufacturer’s specification. The yield of RNA was determined using a NanoDrop 2000 spectrophotometer (Thermo Scientific, USA), and the integrity was evaluated using agarose gel electrophoresis stained with ethidium bromide. According to gene sequences of SET domain-containing proteins in *G. raimondii* (Supplementary Table S2), the primer pairs (Supplementary Table S1) used for real-time quantitative RT-PCR (RT-qPCR) were designed using Roche LCPDS2 software and synthesized by Generay Biotech (Generay, PRC). The amplified fragment lengths were between 75 bp and 200 bp, and the annealing temperature was between 58 °C and 60 °C. The cotton histone3 (AF024716) gene was used as the reference gene.

Quantification was performed with a two-step reaction process: reverse transcription (RT) and PCR. Each RT reaction consisted of 0.5 μg RNA, 2 μl of PrimerScript Buffer, 0.5 μl of oligo dT, 0.5 μl of random 6 mers and 0.5 μl of PrimerScript RT Enzyme Mix I (TaKaRa, Japan), in a total volume of 10 μl. Reactions were performed in a GeneAmp PCR System 9700 (Applied Biosystems, USA) for 15 min at 37 °C, followed by heat inactivation of RT for 5 s at 85 °C. The 10 μl RT reaction mix was then diluted × 10 in nuclease-free water and held at −20 °C. Real-time PCR was performed using LightCycler 480 Real-time PCR Instrument (Roche, Swiss) with 10 μl PCR reaction mixture that included 1 μl of cDNA, 5 μl of 2 × LightCycler 480 SYBR Green I Master (Roche, Swiss), 0.2 μl of forward primer, 0.2 μl of reverse primer and 3.6 μl of nuclease-free water. Reactions were incubated in a 384-well optical plate (Roche, Swiss) at 95 °C for 10 min, followed by 40 cycles of 95 °C for 10 s, 60 °C for 30 s. Each sample was run in triplicate for analysis. At the end of the PCR cycles, melting curve analysis was performed to validate the specific generation of the expected PCR product. PCR efficiency (E) was determined from the slope produced by a RT-qPCR standard curve for each pair of primers using the following equation: E = 10^(−1/slope)^ −1, and all the 53 gene primers yielded RT-qPCR data of good quality with a PCR efficiency >0.9 (Supplementary Table S1). The expression values of SET domain-containing proteins genes tested were normalized with the internal reference gene, and the relative expression levels in tissues and in response to HT stress were calculated with 2^− ΔCT^ and 2^− ΔΔCT^ methods^59^, respectively.

## Additional Information

**How to cite this article**: Huang, Y. *et al*. Identification of SET Domain-Containing Proteins in *Gossypium raimondii* and Their Response to High Temperature Stress. *Sci. Rep*. **6**, 32729; doi: 10.1038/srep32729 (2016).

## Supplementary Material

Supplementary Information

Supplementary Table S1

Supplementary Table S2

Supplementary Table S3

Supplementary Table S4
