# Supplementary material for: Identification of SET Domain-Containing Proteins in Gossypium raimondii and Their Response to High Temperature Stress
Source: Sci Rep. 2016 Sep 7;6:32729. doi: 10.1038/srep32729 (PMC5013442; doi:10.1038/srep32729)
Supplement: Supplementary Information [file srep32729-s1.doc]

**Supplementary Information**

**Identification of SET Domain-Containing Proteins in *Gossypium raimondii* and Their Response to High Temperature Stress**

**Yong Huang****^1^, Yijia Mo^1^, Pengyun Chen^1^, Xiaoling Yuan^1^, Funing Meng^2^, Shengwei Zhu^2, *^, Zhi Liu^1, *^**

^1^ College of Bioscience and Biotechnology, Hunan Agricultural University, Changsha 410128, P. R. China

^2^ Key laboratory of Plant Molecular Physiology, Institute of Botany, Chinese Academy of Sciences, Beijing 100093, P. R. China

**^*^**Corresponding author

Corresponding author:

Zhu S.

Key laboratory of Plant Molecular Physiology, Institute of Botany, Chinese Academy of Sciences, Beijing 100093, P. R. China

e-mail：zhusw@ibcas.ac.cn

Liu Z.

College of Bioscience and Biotechnology, Hunan Agricultural University, Changsha 410128, P. R. China

e-mail: tigerzhiliu@gmail.com

**Supplementary Information**

**Supplementary Figures**

**Supplementary Figure S1** Tissue and organ expression of duplicated *GrKMT*s and *GrRBCMT*s. A heatmap for gene expression patterns was generated with the software MultiExperiment Viewer (MeV).

**Supplementary Figure S2** Expression of duplicated *GrKMT*s and *GrRBCMT*s in response to high temperature. The error bars depict SD and the asterisk shows the corresponding gene significantly up- or downregulated by Student′s *t* test between the treatment and the control (P<0.05).

**Supplementary Tables**

**Supplementary Table S1** Primers list and PCR efficiencies in real-time quantitative RT-PCR.

**Supplementary Table S2** SET domain-containing proteins in plants used in this paper.

**Supplementary Table S3** Sequences and main motifs of the SET domain-containing proteins in this paper.

**Supplementary Table S4** Blastn and Blastp results between homologous genes or proteins pairs.

**
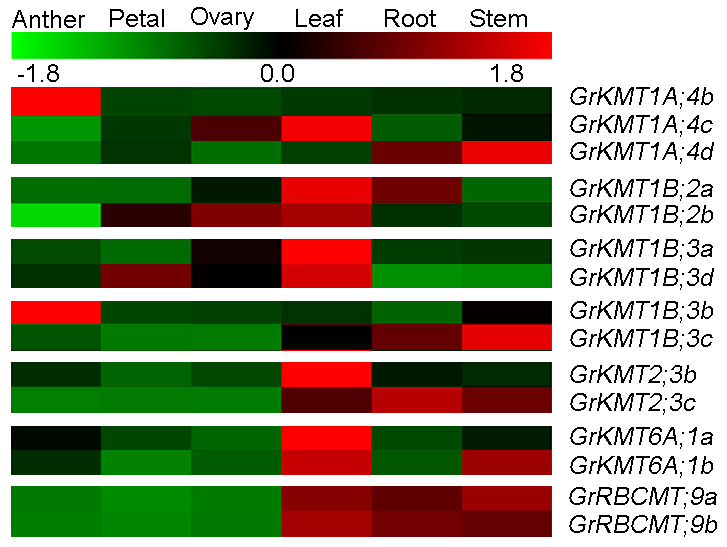
**

**Supplementary Figure S1** Tissue and organ expression of duplicated *GrKMT*s and *GrRBCMT*s. A heatmap for gene expression patterns was generated with the software MultiExperiment Viewer (MeV).

**
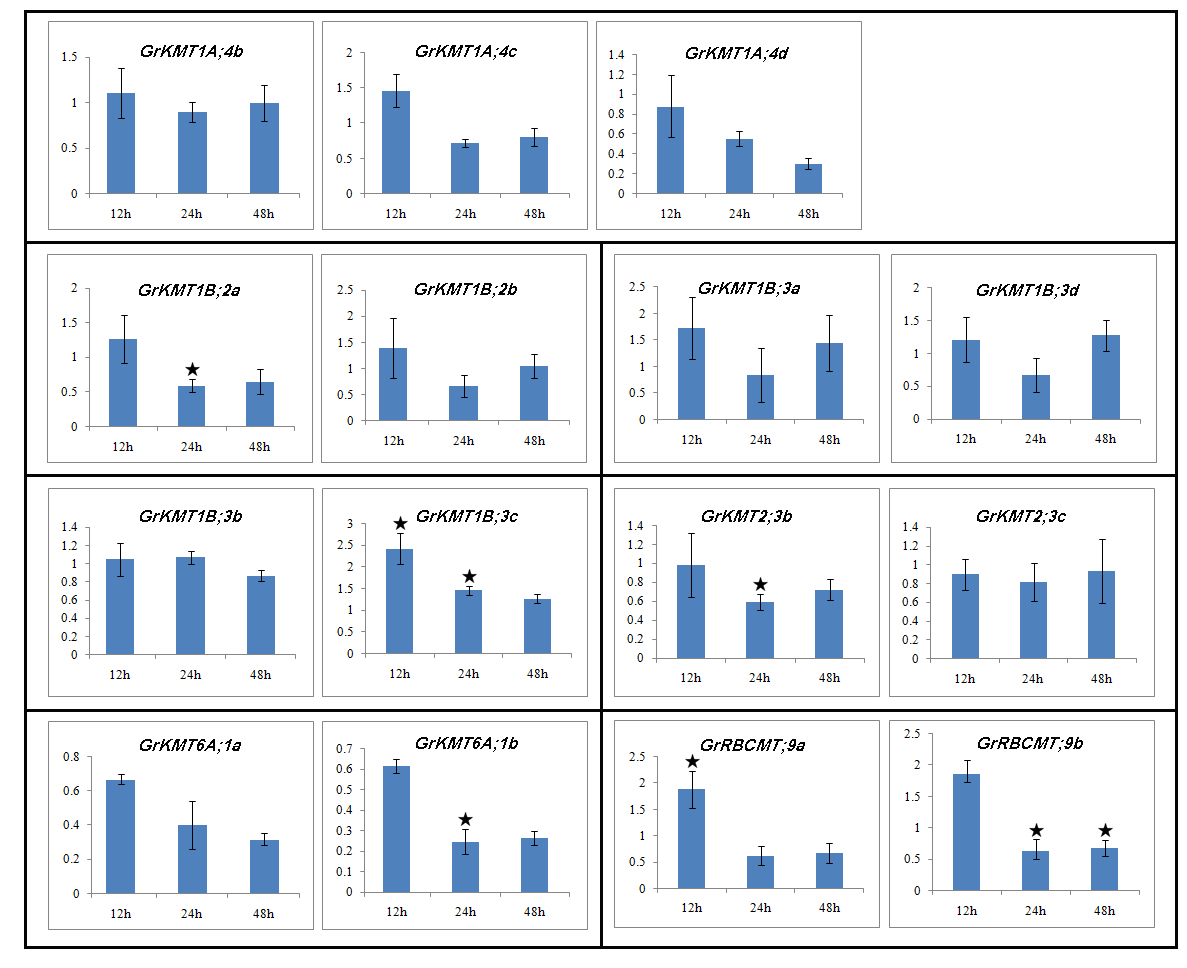
**

**Supplementary Figure S2** Expression of duplicated *GrKMT*s and *GrRBCMT*s in response to high temperature. The error bars depict SD and the asterisk shows the corresponding gene significantly up- or downregulated by Student′s *t* test between the treatment and the control (P<0.05).
