# Supplementary material for: Identification of SET Domain-Containing Proteins in Gossypium raimondii and Their Response to High Temperature Stress
Source: Sci Rep. 2016 Sep 7;6:32729. doi: 10.1038/srep32729 (PMC5013442; doi:10.1038/srep32729)
Supplement: Supplementary Table S1 [file srep32729-s2.pdf]

Supplementary Table S1

Identification of SET Domain-Containing Proteins in *Gossypium raimondii* and Their Response to High Temperature Stress

Yong Huang[1], Yijia Mo[1], Pengyun Chen[1], Xiaoling Yuan[1], Funing Meng[2], Shengwei Zhu[2, *], Zhi Liu[1, *]

[1] College of Bioscience and Biotechnology, Hunan Agricultural University, Changsha 410128, P. R. China

[2] Key laboratory of Plant Molecular Physiology, Institute of Botany, Chinese Academy of Sciences, Beijing 100093, P. R. China

[*]Corresponding author

Corresponding author:

Zhu S.

Key laboratory of Plant Molecular Physiology, Institute of Botany, Chinese Academy of Sciences, Beijing 100093, P. R. China

e-mail：zhusw@ibcas.ac.cn

Liu Z.

College of Bioscience and Biotechnology, Hunan Agricultural University, Changsha 410128, P. R. China

e-mail: tigerzhiliu@gmail.com

Supplementary Table S1 Primers list and PCR efficiencies in real-time quantitative RT-PCR.

No.	Gene Symbol	Forward primer	Reverse primer	Product length(bp)	Ta(℃)	PCR Efficiency*
1	*GrKMT1A;1a*	GTATGCCCTTGATAGCGT	TTCAGGACCTAGAATAGGCG	102	60	0.9934
2	*GrKMT1A;1b*	TGAGTAACGCAGGTTATTCATC	CACCATACTGGAGCTGAAG	139	60	0.9476
3	*GrKMT1A;2*	AAGATGAACGGTGATACTTTGA	GATATGATGGACACACGTACTC	106	60	0.9502
4	*GrKMT1A;3a*	GCTACCAGTATTATAGCTTCCG	TTGCTTGCCTTTCTGCAT	105	60	0.9980
5	*GrKMT1A;3b*	TGTGAAATGTTCGTGTGTGAT	GTAGGGCCACATTCATAGAC	102	60	0.9871
6	*GrKMT1A;3c*	TTGCATTGTCAAGAACGGAG	TGAAGCACTTACAAGAAGGTC	101	60	1.0500
7	*GrKMT1A;4a*	AAGGCAATGTAAGGATTCTAGT	AAGAAAGGGAAAGAAACGGA	126	60	0.9940
8	*GrKMT1A;4b*	CAGTGATAAGGCAAGGCAG	ATAATCTTCGGTGGCGTC	142	60	0.9949
9	*GrKMT1A;4c*	GGGAGTGCCAGTAGAGATAAA	AAGCCCGTCATACACATAG	159	60	0.9759
10	*GrKMT1A;4d*	TTGCTAAGAAACACATTCCTCC	GGCGATCCACATAAGCAC	121	60	0.9241
11	*GrKMT1B;1*	ACTGTCAGGATTGTCCATTAG	GTACAACTCGATTCCCGCA	138	60	0.9436

No.	Gene Symbol	Forward primer	Reverse primer	Product length(bp)	Ta(℃)	PCR Efficiency*
12	*GrKMT1B;2a*	TTGCCCAAGAAGAGTTCAG	AAGGAGGTAAGCTATCACCA	106	60	0.9859
13	*GrKMT1B;2b*	TGAGGTGAAGATTTCTGTAAGC	GACATCTCTGTGCCATTAGT	104	60	0.9799
14	*GrKMT1B;3a*	TAAGACTTCTACATGCCACG	AACGAAATTGAATCCAAGTCAG	129	60	0.9396
15	*GrKMT1B;3b*	AGGAGCTTTACCTCCATCA	GATTTGGCAGTTTATAGTCGC	108	60	0.9303
16	*GrKMT1B;3c*	TAATGGGATCTCTATGGCGTC	CCTAGTGCAAGAACCACC	168	60	0.9325
17	*GrKMT1B;3d*	GTCTGTTCAGGACAGGATT	GCAACCCGCTTCGTTCTA	114	60	0.9353
18	*GrKMT1B;4*	GGAAATTAGGGTTCAGCCTAAG	TCTAGCCATCTACGAGCAT	160	60	0.9515
19	*GrKMT2;1*	TCTGACACAACCAAGTCTCATC	AACCATACTCATATCCAACACC	113	60	0.9565
20	*GrKMT2;2a*	GGTGCTGATCTCTTGAAAGCTA	TATCTGAAATCCTCGGACGG	166	60	0.9959
21	*GrKMT2;2b*	GGAATCAACCTGTGCTCGAA	TTGAGGAACAAAGCATCCG	109	60	0.9592
22	*GrKMT2;3a*	ACTATTTCCATTTCCATCCGT	AGGAACTTTAAGCTCATCGT	134	60	0.9829
23	*GrKMT2;3b*	CTGACCAGTTGCTAAACG	TCCTTCTTGAATGTTTCTGCG	121	60	0.9843

**No.**	**Gene Symbol**	**Forward primer**	**Reverse primer**	**Product length(bp)**	**Ta(℃)**	**PCR Efficiency***
24	*GrKMT2;3c*	TGCAGGTGTCTAGTGGTTAT	TTTCGACAATTTGGAGCCTT	137	60	0.9872
25	*GrKMT3;1a*	TACAGGTTTCCCTCCACCTA	GCAGTGGGATTCCGTATG	113	60	0.9865
26	*GrKMT3;1b*	TATGCAAAGGTGAAGCCG	CAGCAGTCGATGAAACCAA	111	60	0.9626
27	*GrKMT3;2*	GAACAAGTGAAGCCTTGTG	AGTCTGTAACATTGGTTTCGT	113	60	1.0181
28	*GrKMT3;3*	GTTTAGACATTTGGCTGCTT	GAATGTTCAACCTTTGACTCAC	109	60	0.9625
29	*GrKMT3;4*	ACAAATGCCAATCATTCTCG	ACTTCTACTAAGTGTCCACAAC	111	60	0.9554
30	*GrKMT6A;1a*	GATCTAGGAGGAAAGAGTGGTC	CACCATTGCTAGTAGCCTG	102	60	0.9927
31	*GrKMT6A;1b*	ATGCCAAGGTCATTATGGT	GCTCGTAACGGTAGTCATAGA	102	60	0.9815
32	*GrKMT6A;3*	TTGTAGGAATTGTTGGGTTAGT	TTGCCAAGAGTATTCTCTGC	122	60	0.9809
33	*GrKMT6B;1*	CGGTAGAAGCAGATGCAC	GGTCATCATGCTATCACAGT	110	60	1.0111
34	*GrKMT6B;2*	CATCAACAACTTCTCACCGT	TTTCTCCCATCCCTGCAT	116	60	1.0064
35	*GrKMT7;1*	GCCGAAGGACCGAATATG	CATCTCTCTGTCCAGTGAAC	100	60	0.9923

No.	Gene Symbol	Forward primer	Reverse primer	Product length(bp)	Ta(℃)	PCR Efficiency*
36	*GrRBCMT;1a*	GCCTTGACCTCAACCTTTATAG	CAACAGGATTCAGGATCACC	106	60	0.9806
37	*GrRBCMT;1b*	TCTGGGTGATTGTACTGCT	ATTGAATGATACGGCTCCTC	134	60	0.9969
38	*GrRBCMT;4*	TTTAGGAACGGTTGCGTG	TCTATGCAAATCCTCAAGAGTG	146	60	0.9833
39	*GrRBCMT;5*	CTCCCTCTGATTGATATGTGC	TTATTGACATGAACCTGCCTTT	111	60	0.9864
40	*GrRBCMT;6a*	GGAACCTGGCTTTCAGAG	GGGAATGAGATTACAAGGGTC	122	60	1.0033
41	*GrRBCMT;6b*	GAAATGCCAACATCAGTCGAA	CTCGCCTCCAAATACTGC	117	60	1.0009
42	*GrRBCMT;7a*	AATGCTTACATGCAACTTCC	CAGTTCACCACTCCTCATA	110	60	1.0143
43	*GrRBCMT;7b*	TGTTTCTTCCTGATTGGCCT	AAGCTCTCAGTTCACCAC	108	60	1.0050
44	*GrRBCMT;7c*	GCAAACATTGGCATGATCT	AACTCTCACGAATGTCCG	133	60	0.9852
45	*GrRBCMT;8*	TTGATGGCCGGTTAGCTC	AAAGTGGCAAGTAGATTACCT	135	60	0.9808
46	*GrRBCMT;9a*	ATTACAGTCCGAGTTGAAGT	CTTGATAGATGTCCAATGCC	107	60	0.9933
47	*GrRBCMT;9b*	ATTACAGTCCGAGTTGAAGT	GCTCTTGATAGATGTCCAATGC	110	60	0.9223

No.	Gene Symbol	Forward primer	Reverse primer	Product length(bp)	Ta(℃)	PCR Efficiency*
48	*GrS-ET;1*	CGTATGCTGAGGTATATGGTTC	CCTTCAACAGTCTCTCATCAC	123	60	0.9927
49	*GrS-ET;2*	CTTATCGAAGTAAGGACGAGC	CCTAGAAGAGACAGTGGCAA	148	60	0.9752
50	*GrS-ET;3*	GCATTGCTTGCAGATTACG	TGAATGCTATGAAACAACCACT	103	60	0.9947
51	*GrS-ET;4a*	ATCGTTGTTTACTCCGCT	ATAATGGACACGGCACTAC	112	60	1.0012
52	*GrS-ET;4b*	CAGATGATGCTATCAAGTCGT	TCCTCCAACTTCATCAACAG	103	60	1.0021
53	*histone 3*	CATGATGGTAGTGGTAGGTTC	GGCAAAGCAACTTTCGATT	130	60	0.9946

*Note: Standard curve assays were performed for each pair of primers by creating a ten-fold dilution scheme, PCR efficiency (E) was determined from the slope of the log-linear portion of the calibration curve using the following equation: $E=10^{(-1/slope)}-1$. The correlation coefficient (R^2) for each pair of primers >0.99.
