# Supplementary material for: Identification of SET Domain-Containing Proteins in Gossypium raimondii and Their Response to High Temperature Stress
Source: Sci Rep. 2016 Sep 7;6:32729. doi: 10.1038/srep32729 (PMC5013442; doi:10.1038/srep32729)
Supplement: Supplementary Table S2 [file srep32729-s3.pdf]

Supplementary Table S2

Identification of SET Domain-Containing Proteins in *Gossypium raimondii* and Their Response to High Temperature Stress

Yong Huang[1], Yijia Mo[1], Pengyun Chen[1], Xiaoling Yuan[1], Funing Meng[2], Shengwei Zhu[2, *], Zhi Liu[1, *]

[1] College of Bioscience and Biotechnology, Hunan Agricultural University, Changsha 410128, P. R. China

[2] Key laboratory of Plant Molecular Physiology, Institute of Botany, Chinese Academy of Sciences, Beijing 100093, P. R. China

[*]Corresponding author

Corresponding author:

Zhu S.

Key laboratory of Plant Molecular Physiology, Institute of Botany, Chinese Academy of Sciences, Beijing 100093, P. R. China

e-mail：zhusw@ibcas.ac.cn

Liu Z.

College of Bioscience and Biotechnology, Hunan Agricultural University, Changsha 410128, P. R. China

e-mail: tigerzhiliu@gmail.com

Supplementary Table S2 SET domain-containing Proteins in plants used in this paper.

Arabidopsis thaliana					*Oryza sativa*		
**Name**	**Synonyms**	**Formal Name**	**Location**	**Protein Group**	**Name**	**Formal Name**	**Locus Name**
AtKMT1A;1	SDG33	SUVH4	At5g13960	ARATH_Kryptonite Group	OsKMT1A;1	SDG714	Os01g70220
AtKMT1A;2a	SDG3	SUVH2	At2g33290	ARATH_SUVH2/SUVH9 Group	OsKMT1A;2a	SDG726	Os07g25450
AtKMT1A;2b	SDG22	SUVH9	At4g13460	ARATH_SUVH2/SUVH9 Group	OsKMT1A;2b	SDG715	Os08g45130
AtKMT1A;3a	SDG9	SUVH5	At2g35160	ARATH_SUVH5/SUVH6 Group	OsKMT1A;3a	SDG710	Os08g30910
AtKMT1A;3b	SDG23	SUVH6	At2g22740	ARATH_SUVH5/SUVH6 Group	OsKMT1A;3b	SDG727	Os09g19830
AtKMT1A;4a	SDG32	SUVH1	At5g04940	SUVH1/SUVH3 Group	OsKMT1A;3c	SDG703	Os04g45990
AtKMT1A;4b	SDG19	SUVH3	At1g73100	SUVH1/SUVH3 Group	OsKMT1A;4a	SDG704	Os11g38900
AtKMT1A;4c	SDG17	SUVH7	At1g17770	SUVH1/SUVH3 Group	OsKMT1A;4b	SDG713	Os03g20430
AtKMT1A;4d	SDG21	SUVH8	At2g24740	SUVH1/SUVH3 Group	OsKMT1A;4c	SDG728	Os05g41170
AtKMT1A;4e	SDG11	SUVH10	At2g05900	SUVH1/SUVH3 Group	OsKMT1A;4d	SDG709	Os01g59620
AtKMT1B;1	SDG31	SUVR4	At3g04380	ARATH_SUVR4 Group	OsKMT1A;4e	SDG734	Os12g03460

AtKMT1B;2a	SDG13	SUVR1	At1g04050	ARATH_SUVR4 Group	OsKMT1A;4f	SDG733	Os11g03700
AtKMT1B;2b	SDG18	SUVR2	At5g43990	ARATH_SUVR4 Group	OsKMT1B;1	SDG742	N
AtKMT1B;3	SDG6	SUVR5	At2g23740	ARATH_SUVR5 Group	OsKMT1B;2	SDG712	Os02g40770
AtKMT1B;4	SDG20	SUVR3	At3g03750	ARATH_SUVR3 Group	OsKMT1B;3	SDG706	Os02g47900
					OsKMT1B;4	SDG729	Os01g56540
AtKMT2;1a	SDG27	ATX1	At2g31650	ARATH_ATX1 Group	OsKMT2;1	SDG723	Os09g04890
AtKMT2;1b	SDG30	ATX2	At1g05830	ARATH_ATX1 Group	OsKMT2;2a	SDG717	Os12g41900
AtKMT2;2	SDG25	ATXR7	At5g42400	ARATH_ATXR7 Group	OsKMT2;2b	SDG732	Os09g38440
AtKMT2;3a	SDG14	ATX3; SET14	At3g61740	ARATH_ATX4/5 Group	OsKMT2;3a	SDG721	Os01g11950
AtKMT2;3b	SDG16	ATX4	At4g27910	ARATH_ATX4/5 Group	OsKMT2;3b	SDG705	Os01g46700
AtKMT2;3c	SDG29	ATX5	At5g53430	ARATH_ATX4/5 Group			
AtKMT3;1	SDG8	EFS,ASHH2; SET8	At1g77300	ARATH_EFS Group	OsKMT3;1	SDG725	Os02g34850

AtKMT3;2	SDG26	ASHH1	At1g76710	ARATH_ASHH1 Group	OsKMT3;2	SDG708	Os04g34980
AtKMT3;3	SDG4	ASHR3; SET4	At4g30860	ASH1 Group	OsKMT3;3a	SDG736	Os02g39800
AtKMT3;4a	SDG7	ASHH3	At2g44150	ASH1 Group	OsKMT3;3b	SDG707	Os08g34370
AtKMT3;4b	SDG24	ASHH4	At3g59960	ASH1 Group	OsKMT3;4	SDG724	Os09g13740
AtKMT6A;1	SDG1	CLF	At2g23380	ARATH_CLF Group	OsKMT6A;1a	SDG718	Os03g19480
AtKMT6A;2	SDG10	EZA1	At4g02020	ARATH_EZA1 Group	OsKMT6A;1b	SDG711	Os06g16390
AtKMT6A;3	SDG5	FIS1; SET5	At1g02580	ARATH_EZA1 Group	OsKMT6B;1a	SDG720	Os01g73460
AtKMT6B;1	SDG15	ATXR5; SET15	At5g09790	ARATH_ATXR5 Group	OsKMT6B;1b	SDG730	Os02g03030
AtKMT6B;2	SDG34	ATXR6	At5g24330	ARATH_ATXR5 Group			
AtKMT7;1	SDG2	ATXR3; DL3635W; FCAALL.214; SET2	At4g15180	TRR_TRX	OsKMT7;1	SDG701	Os08g08210
AtRBCMT1	SDG40	At4g15180	At5g17240	Rubisco methyltransferase family	OsRBCMT3		Os06g03676

AtRBCMT2	SDG41		At1g43245	S-ET interrupted	OsRBCMT4a		Os09g24530
AtRBCMT3	SDG42		At1g01920	Rubisco methyltransferase family	OsRBCMT4b		Os01g65730
AtRBCMT4	SDG43		At1g14030	Rubisco methyltransferase family	OsRBCMT5		Os02g50100
AtRBCMT5		AEE30553.1	AT1G24610	Rubisco methyltransferase family	OsRBCMT6		Os08g14660
AtRBCMT6		AEC06814	AT2G18850	Rubisco methyltransferase family	OsRBCMT7a		Os02g49326
AtRBCMT7		AEE74585.1	AT3G07670	Rubisco methyltransferase family	OsRBCMT7b		Os07g28840
AtRBCMT8		AAX12881	AT3G55080	Rubisco methyltransferase family	OsRBCMT9		Os05g50980
AtRBCMT9		AEE84278	AT4G20130	Rubisco methyltransferase family			
AtS-ET;1	SDG35		At1g26760	S-ET interrupted	OsS-ET;1	SDG739	Os03g07260
AtS-ET;2	SDG38	ATXR4	At5g06620	S-ET interrupted	OsS-ET;2	SDG741	Os10g27060
AtS-ET;3	SDG36	ATXR2	At3g21820	S-ET interrupted	OsS-ET;3	SDG722	Os04g53700
AtS-ET;4a	SDG39	ASHR2	At2g19640		OsS-ET;4a	SDG740	Os08g10470
AtS-ET;4b	SDG37	ASHR1	At2g17900	S-ET interrupted	OsS-ET;4b	SDG716	Os03g49730

Gossypium raimondii							
**Name**	**Locus Name**	**Location**	**Genomic (bp)**	**Transcript (bp)**	**CDS (bp)**	**Protein (aa)**	**No. of exon**
GrKMT1A;1a	Gorai.012G082600	Chr12:13642672..13650189 forward	7518	2431	2106	701	15
GrKMT1A;1b	Gorai.005G196900	Chr05:57079137..57089243 reverse	10107	3315	2976	991	15
GrKMT1A;2	Gorai.006G254500	Chr06:49739100..49742394 forward	3295	3169	2082	693	1
GrKMT1A;3a	Gorai.004G192900	Chr04:51039230..51043193 reverse	3964	3195	3195	1064	2
GrKMT1A;3b	Gorai.008G231400	Chr08:51753532..51757573 reverse	4042	3257	2760	919	2
GrKMT1A;3c	Gorai.003G111600	Chr03:34185125..34189264 reverse	4140	3200	2196	731	4
GrKMT1A;4a	Gorai.007G350900	Chr07:58110934..58115639 reverse	4706	2968	2094	697	1
GrKMT1A;4b	Gorai.012G020600	Chr12:2539490..2542558 reverse	3069	2797	1995	664	1
GrKMT1A;4c	Gorai.009G301500	Chr09:27029059..27032532 forward	3474	2646	1974	657	1
GrKMT1A;4d	Gorai.013G003600	Chr13:266710..269484 reverse	2775	2598	2004	667	1
GrKMT1B;1	Gorai.013G119500	Chr13:29778225..29780285 forward	2061	1415	1281	426	4

GrKMT1B;2a	Gorai.004G222200	Chr04:55677407..55683589 reverse	6183	2727	2388	795	9
GrKMT1B;2b	Gorai.007G149600	Chr07:12646664..12654128 reverse	7465	2691	2505	834	9
GrKMT1B;3a	Gorai.003G018400	Chr03:1381180..1383441 reverse	2262	733	279	92	3
GrKMT1B;3b	Gorai.003G018700	Chr03:1392709..1401038 reverse	8330	5445	4773	1590	11
GrKMT1B;3c	Gorai.003G018800	Chr03:1416523..1424629 reverse	8107	5155	4488	1495	11
GrKMT1B;3d	Gorai.007G266700	Chr07:44533398..44537743 reverse	4346	1185	708	235	6
GrKMT1B;4	Gorai.007G256400	Chr07:41643942..41646490 reverse	2549	1455	1086	361	2
GrKMT2;1	Gorai.011G144000	Chr11:22883173..22897018 reverse	13846	4053	3204	1067	24
GrKMT2;2a	Gorai.005G115300	Chr05:22538082..22545612 reverse	7531	4525	3654	1217	16
GrKMT2;2b	Gorai.001G035300	Chr01:3257118..3269066 forward	11949	6863	5292	1763	17
GrKMT2;3a	Gorai.008G125100	Chr08:36443252..36451251 reverse	8000	3741	3060	1019	23
GrKMT2;3b	Gorai.008G269400	Chr08:54810676..54819536 forward	8861	3989	3276	1091	23
GrKMT2;3c	Gorai.012G003100	Chr12:372633..382302 forward	9670	4116	3255	1084	23

GrKMT3;1a	Gorai.005G087500	Chr05:11248345..11262806 reverse	14462	7635	6999	2332	17
GrKMT3;1b	Gorai.009G248900	Chr09:20111117..20126421 forward	15305	6452	5913	1970	17
GrKMT3;2	Gorai.010G131600	Chr10:28832644..28838929 forward	6286	1748	1524	507	10
GrKMT3;3	Gorai.013G240900	Chr13:56041038..56049641 reverse	8604	1901	1470	489	10
GrKMT3;4	Gorai.010G220300	Chr10:58934343..58946325 reverse	11983	2116	1098	365	12
GrKMT6A;1a	Gorai.007G215200	Chr07:23786729..23796782 reverse	10054	3020	2796	931	16
GrKMT6A;1b	Gorai.011G106100	Chr11:12348918..12357283 reverse	8366	3334	2760	919	17
GrKMT6A;3	Gorai.008G139100	Chr08:38989212..38996424 forward	7213	3077	2661	886	17
GrKMT6B;1	Gorai.003G068200	Chr03:14413107..14417268 forward	4162	1203	1203	400	5
GrKMT6B;2	Gorai.003G122100	Chr03:36675863..36678677 reverse	2815	2046	1038	345	6
GrKMT7;1	Gorai.005G208600	Chr05:58938189..58951843 forward	13655	8698	7440	2479	19

GrRBCMT;1a	Gorai.010G134600	Chr10:30193871..30196802 reverse	2932	1965	1893	630	4
GrRBCMT;1b	Gorai.013G010300	Chr13:676119..684073 reverse	7955	2680	2325	774	14
GrRBCMT;4	Gorai.005G142600	Chr05:38913199..38917184 forward	3986	2583	1467	488	6
GrRBCMT;5	Gorai.008G168000	Chr08:43918275..43922924 reverse	4650	2412	1428	475	5
GrRBCMT;6a	Gorai.004G100200	Chr04:17619271..17630119 reverse	10849	2496	1626	541	11
GrRBCMT;6b	Gorai.008G054300	Chr08:7888400..7893449 forward	5050	2268	1470	489	5
GrRBCMT;7a	Gorai.001G190900	Chr01:31667729..31672356 forward	4628	2055	1677	558	11
GrRBCMT;7b	Gorai.004G061300	Chr04:6062981..6068761 forward	5781	2026	1470	489	12
GrRBCMT;7c	Gorai.012G045900	Chr12:5737964..5743372 forward	5409	1774	1617	538	5
GrRBCMT;8	Gorai.012G067000	Chr12:9594930..9616282 forward	21353	1709	1440	479	14
GrRBCMT;9a	Gorai.009G012700	Chr09:1041503..1045913 reverse	4411	1853	1452	483	12
GrRBCMT;9b	Gorai.N022300	scaffold_456:1014..3946 reverse	2933	1005	1005	334	11
GrS-ET;1	Gorai.013G087800	Chr13:13403753..13405518 reverse	1766	1766	1599	532	1

GrS-ET;2	Gorai.006G144800	Chr06:40300737..40304893 reverse	4157	1681	1014	337	7
GrS-ET;3	Gorai.002G016300	Chr02:1060812..1065736 reverse	4925	2169	1452	483	15
GrS-ET;4a	Gorai.006G259000	Chr06:50007748..50010061 forward	2314	1545	1173	390	1
GrS-ET;4b	Gorai.005G091200	Chr05:12292855..12297894 reverse	5040	1832	1491	496	14
