# Supplementary material for: Identification of SET Domain-Containing Proteins in Gossypium raimondii and Their Response to High Temperature Stress
Source: Sci Rep. 2016 Sep 7;6:32729. doi: 10.1038/srep32729 (PMC5013442; doi:10.1038/srep32729)
Supplement: Supplementary Table S3 [file srep32729-s4.pdf]

Supplementary Table S3

Identification of SET Domain-Containing Proteins in *Gossypium raimondii* and Their Response to High Temperature Stress

Yong Huang[1], Yijia Mo[1], Pengyun Chen[1], Xiaoling Yuan[1], Funing Meng[2], Shengwei Zhu[2,*], Zhi Liu[1,*]

[1] College of Bioscience and Biotechnology, Hunan Agricultural University, Changsha 410128, P. R. China

[2] Key laboratory of Plant Molecular Physiology, Institute of Botany, Chinese Academy of Sciences, Beijing 100093, P. R. China

[*]Corresponding author

Corresponding author:

Zhu S.

Key laboratory of Plant Molecular Physiology, Institute of Botany, Chinese Academy of Sciences, Beijing 100093, P. R. China

e-mail：zhusw@ibcas.ac.cn

Liu Z.

College of Bioscience and Biotechnology, Hunan Agricultural University, Changsha 410128, P. R. China

e-mail: tigerzhiliu@gmail.com

Supplementary Table S3 Sequences and main motifs of the SET domain-containing proteins in this paper

SRA	Pre-SET	SET	Post-SET	AWS	SANT	GYF	F-box	PB1
zf-DBF	PWWP	PHD	FYRN FYRC	MyTH4	Rubis-subs-bind	TRR		

Domains abbreviation:

SET: Su(var), E(z), and Trithorax;

SRA: SET- and RING-associated;

AWS: Associated with SET;

SANT: SWI3-ADA2-N-CoR-TFIIIB;

GYF: Glycine-tyrosine-phenylalanine;

PB1: Phox and Bem1;

zf-DBF： DBF zinc finger;

PWWP： Pro-Trp-Trp-Pro;

PHD： Plant homeodomain;

FYRN: F/Y-rich N-terminus;

FYRC: F/Y-rich C-terminus;

MyTH4: Myosin Tail Homology;

Rubis-subs-bind: Rubisco substrate-binding;

TRR: Tetratricopeptide repeat

KMT1A

>AtKMT1A;1

MAGKRKRANAPDQTERRSSVRVQKVRQKALDEKARLVQERVKLLSDRKSEICVDDTELHEK
EEENVDGSPKRRSPPKLTAMQKGKQKLSVSLNGKDVNLEPHLKVTKCLRLFNKQYLLCVQA
KLSRPDLKGVTEMIKAKAILYPRKIIGDLPGIDVGHRFFSRAEMCAVGFHNHWLNGIDYMSM
EYEKEYSNYKLPLAVSIVMSGQYEDDLDNADTVTYTGQGGHNLTGNKRQIKDQLLERGNLA
LKHCCEYNVPVRVTRGHNCKSSYTKRVYTYDGLYKVEKFWAQKGVSGFTVYKYRLKRLEG
QPELTTDQVNFVAGRIPTSTSEIEGLVCEDISGGLEFKGIPATNRVDDSPVSPTSGFTYIKSLIIEP
NVIIPKSSTGCNCRGSCTDSKKCACAKLNGGNFPYVDLNDGRLIESRDVVFECGPHCGCGPKC
VNRTSQKRLRFNLEVFRSAKKGWAVRSWEYIPAGSPVCEYIGVVRRTADVDTISDNEYIFEID
CQQTMQGLGGRQRRLRDVAVPMNNGVSQSSEDENAPEFCIDAGSTGNFARFINHSCEPNLFV
QCVLSSHQDIRLARVVLFAADNISPMQELTYDYGYALDSVHGPDGKVKQLACYCGALNCRK
RLY

>AtKMT1A;2a

MSTLLPFPDLNLMPDSQSSTAGTTAGDTVVTGKLEVKSEPIEEWQTPPSSTSDQSANTDLIAEF
IRISELFRSAFKPLQVKGLDGVSVYGLDSGAIVAVPEKENRELIEPPPGFKDNRVSTVVVSPKF
ERPRELARIAILGHEQRKELRQVMKRTRMTYESLRIHLMAESMKNHVLGQGRRRRSDMAAA
YIMRDRGLWLNYDKHIVGPVTGVEVGDIFFYRMELCVLGLHGQTQAGIDCLTAERSATGEPI
ATSIVVSGGYEDDEDTGDVLVYTGHGGQDHQHKQCDNQRLVGGNLGMERSMHYGIEVRVI
RGIKYENSISSKVYVYDGLYKIVDWWFAVGKSGFGVFKFRLVRIEGQPMMGSAVMRFAQTL
RNKPSMVRPTGYVSFDLSNKKENVPVFLYNDVDGDQEPRHYEYIAKAVFPPGIFGQGGISRT
GCECKLSCTDDCLCARKNGGEFAYDDNGHLLKGKHVVFECGEFCTCGPSCKSRVTQKGLRN
RLEVFRSKETGWGVRTLDLIEAGAFICEYAGVVVTRLQAEILSMNGDVMVYPGRFTDQWRN
WGDLSQVYPDFVRPNYPSLPPLDFSMDVSRMRNVACYISHSKEPNVMVQFVLHDHNHLMFP
RVMLFALENISPLAELSLDYGLADEVNGKLAICN

>AtKMT1A;2b

MGSSHIPLDPSLNPSPSLIPKLEPVTESTQNLAFQLPNTNPQALISSAVSDFNEATDFSSDYNTV
AESARSAFAQRLQRHDDVAVLDSLTGAIVPVEENPEPEPNPYSTSDSSPSVATQRPRPQPRSSE
LVRITDVGPESERQFREHVRKTRMIYDSLRMFLMMEEAKRNGVGGRRARADGKAGKAGSM
MRDCMLWMNRDKRIVGSIPGVQVGDIFFFRFELCVMGLHGHPQSGIDFLTGSLSSNGEPIATS
VIVSGGYEDDDDQGDVIMYTGQGGQDRLGRQAEHQRLEGGNLAMERSMYYGIEVRVIRGL
KYENEVSSRVYVYDGLFRIVDSWFDVGKSGFGVFKYRLERIEGQAEMGSSVLKFARTLKTNP
LSVRPRGYINFDISNGKENVPVYLFNDIDSDQEPLYYEYLAQTSFPPGLFVQQSGNASGCDCV
NGCGSGCLCEAKNSGEIAYDYNGTLIRQKPLIHECGSACQCPPSCRNRVTQKGLRNRLEVFRS
LETGWGVRSLDVLHAGAFICEYAGVALTREQANILTMNGDTLVYPARFSSARWEDWGDLSQ
VLADFERPSYPDIPPVDFAMDVSKMRNVACYISHSTDPNVIVQFVLHDHNSLMFPRVMLFAA
ENIPPMTELSLDYGVVDDWNAKLAICN

>AtKMT1A;3a

MVHSESSILSSLRGGDGGGIPCSKDELAINGSYTDPMGRRKSKRFKVAAESEFSPDFGSITRQL
RSRRMQKEFTVETYETRNVSDVCVLSSQADVELIPGEIVAERDSFKSVDCNDMSVGLTEGAE
SLGVNMQEPMKDRNMPENTSEQNMVEVHPPSISLPEEDMMGSVCRKSITGTKELHGRTISVG
RDLSPNMGSKFSKNGKTAKRSISVEEENLVLEKSDSGDHLGPSPEVLELEKSEVWIITDKGVV
MPSPVKPSEKRNGDYGEGSMRKNSERVALDKKRLASKFRLSNGGLPSCSSSGDSARYKVKET

MRLFHETCKKIMQEEEARPRKRDGGNFKVVCEASKILKSKGKNLYSGTQIIGTVPGVEVGDE
FQYRMELNLLGIHRPSQSGIDYMKDDGGELVATSIVSSGGYNDVLDNSDVLIYTGQGGNVGK
KKNNEPPKDQQLVTGNLALKNSINKKNPVRVIRGIKNTTLQSSVVAKNYVYDGLYLVEEYW
EETGSHGKLVFKFKLRRIPGQPELPWKEVAKSKKSEFRDGLCNVDITEGKETLPICAVNNLDD
EKPPPFIYTAKMIYPDWCRPIPPKSCGCTNGCSKSKNCACIVKNGGKIPYYDGAIVEIKPLVYE
CGPHCKCPPSCNMRVSQHGIKIKLEIFKTESRGWGVRSLESIPIGSFICEYAGELLEDKQAESLT
GKDEYLFDLGDEDDPFTINAAQKGNIGRFINHSCSPNLYAQDVLYDHEEIRIPHIMFFALDNIP
PLQELSYDYNYKIDQVYDSNGNIKKKFCYCGSAECSGRLY

>AtKMT1A;3b

MEMGVMENLMVHTEISKVKSQSNGEVEKRGVSVLENGGVCKLDRMSGLKFKRRKVFAVRD
FPPGCGSRAMEVKIACENGNVVEDVKVVESLVKEEESLGQRDASENVSDIRMAEPVEVQPLR
ICLPGGDVVRDLSVTAGDECSNSEQIVAGSGVSSSSGTENIVRDIVVYADESSLGMDNLDQTQ
PLEIEMSDVAVAKPRLVAGRKKAKKGIACHSSLKVVSREFGEGSRKKKSKKNLYWRDRESL
DSPEQLRILGVGTSSGSSSGDSSRNKVKETLRLFHGVCRKILQEDEAKPEDQRRKGKGLRIDFE
ASTILKRNGKFLNSGVHILGEVPGVEVGDEFQYRMELNILGIHKPSQAGIDYMKYGKAKVAT
SIVASGGYDDHLDNSDVLTYTGQGGNVMQVKKKGEELKEPEDQKLITGNLALATSIEKQTPV
RVIRGKHKSTHDKSKGGNYVYDGLYLVEKYWQQVGSHGMNVFKFQLRRIPGQPELSWVEV
KKSKSKYREGLCKLDISEGKEQSPISAVNEIDDEKPPLFTYTVKLIYPDWCRPVPPKSCCCTTR
CTEAEARVCACVEKNGGEIPYNFDGAIVGAKPTIYECGPLCKCPSSCYLRVTQHGIKLPLEIFK
TKSRGWGVRCLKSIPIGSFICEYVGELLEDSEAERRIGNDEYLFDIGNRYDNSLAQGMSELML
GTQAGRSMAEGDESSGFTIDAASKGNVGRFINHSCSPNLYAQNVLYDHEDSRIPHVMFFAQD
NIPPLQELCYDYNYALDQVRDSKGNIKQKPCFCGAAVCRRRLY

>AtKMT1A;4a

MERNGGHYTDKTRVLDIKPLRTLRPVFPSGNQAPPFVCAPPFGPFPPGFSSFYPFSSSQANQHT
PDLNQAQYPPQHQQPQNPPPVYQQQPPQHASEPSLVTPLRSFRSPDVSNGNAELEGSTVKRRI
PKKRPISRPENMNFESGINVADRENGNRELVLSVLMRFDALRRRFAQLEDAKEAVSGIIKRPD
LKSGSTCMGRGVRTNTKKRPGIVPGVEIGDVFFFRFEMCLVGLHSPSMAGIDYLVVKGETEE
EPIATSIVSSGYYDNDEGNPDVLIYTGQGGNADKDKQSSDQKLERGNLALEKSLRRDSAVRVI
RGLKEASHNAKIYIYDGLYEIKESWVEKGKSGHNTFKYKLVRAPGQPPAFASWTAIQKWKT
GVPSRQGLILPDMTSGVESIPVSLVNEVDTDNGPAYFTYSTTVKYSESFKLMQPSFGCDCANL
CKPGNLDCHCIRKNGGDFPYTGNGILVSRKPMIYECSPSCPCSTCKNKVTQMGVKVRLEVFK
TANRGWGLRSWDAIRAGSFICIYVGEAKDKSKVQQTMANDDYTFDTTNVYNPFKWNYEPG
LADEDACEEMSEESEIPLPLIISAKNVGNVARFMNHSCSPNVFWQPVSYENNSQLFVHVAFFA
ISHIPPMTELTYDYGVSRPSGTQNGNPLYGKRKCFCGSAYCRGSFG

>AtKMT1A;4b

MQGVPGFNTVPNPNHYDKSIVLDIKPLRSLKPVFPNGNQGPPFVGCPPFGPSSSEYSSFFPFGA
QQPTHDTPDLNQTQNTPIPSFVPPLRSYRTPTKTNGPSSSSGTKRGVGRPKGTTSVKKKEKKT
VANEPNLDVQVVKKFSSDFDSGISAAEREDGNAYLVSSVLMRFDAVRRRLSQVEFTKSATSK
AAGTLMSNGVRTNMKKRVGTVPGIEVGDIFFSRIEMCLVGLHMQTMAGIDYIISKAGSDEES
LATSIVSSGRYEGEAQDPESLIYSGQGGNADKNRQASDQKLERGNLALENSLRKGNGVRVVR
GEEDAASKTGKIYIYDGLYSISESWVEKGKSGCNTFKYKLVRQPGQPPAFGFWKSVQKWKE
GLTTRPGLILPDLTSGAESKPVSLVNDVDEDKGPAYFTYTSSLKYSETFKLTQPVIGCSCSGSC
SPGNHNCSCIRKNDGDLPYLNGVILVSRRPVIYECGPTCPCHASCKNRVIQTGLKSRLEVFKTR
NRGWGLRSWDSLRAGSFICEYAGEVKDNGNLRGNQEEDAYVFDTSRVFNSFKWNYEPELV

DEDPSTEVPEEFNLPSPLLISAKKFGNVARFMNHSCSPNVFWQPVIREGNGESVIHIAFFAMRH
IPPMAELTYDYGISPTSEARDESLLHGQRTCLCGSEQCRGSFG

>AtKMT1A;4c

MDKSIPIKAIPVACVRPDLVDDVTKNTSTIPTMVSPVLTNMPSATSPLLMVPPLRTIWPSNKE
WYDGDAGPSSTGPIKREASDNTNDTAHNTFAPPPEMVIPLITIRPSDDSSNYSCDAGAGPSTGP
VKRGRGRPKGSKNSTPTEPKKPKVYDPNSLKVTSRGNFDSEITEAETETGNQEIVDSVMMRF
DAVRRRLCQINHPEDILTTASGNCTKMGVKTNTRRRIGAVPGIHVGDIFYYWGEMCLVGLHK
SNYGGIDFFTAAESAVEGHAAMCVVTAGQYDGETEGLDTLIYSGQGGTDVYGNARDQEMK
GGNLALEASVSKGNDVRVVRGVIHPHENNQKIYIYDGMYLVSKFWTVTGKSGFKEFRFKLV
RKPNQPPAYAIWKTVENLRNHDLIDSRQGFILEDLSFGAELLRVPLVNEVDEDDKTIPEDFDYI
PSQCHSGMMTHEFHFDRQSLGCQNCRHQPCMHQNCTCVQRNGDLLPYHNNILVCRKPLIYE
CGGSCPCPDHCPTRLVQTGLKLHLEVFKTRNCGWGLRSWDPIRAGTFICEFAGLRKTKEEVE
EDDDYLFDTSKIYQRFRWNYEPELLLEDSWEQVSEFINLPTQVLISAKEKGNVGRFMNHSCSP
NVFWQPIEYENRGDVYLLIGLFAMKHIPPMTELTYDYGVSCVERSEEDEVLLYKGKKTCLCG
SVKCRGSFT

>AtKMT1A;4d

MVSTPPTLLMLFDDGDAGPSTGLVHREKSDAVNEEAHATSVPPHAPPQTLWLLDNFNIEDSY
DRDAGPSTGPVHRERSDAVNEEAHATSIPPHAPPQTLWLLDNFNIEDSYDRDAGPSTSPIDRE
ASHEVNEDAHATSAPPHVMVSPLQNRRPFDQFNNQPYDASAGPSTGPGKRGRGRPKGSKNG
SRKPKKPKAYDNNSTDASAGPSSGLGKRRCGRPKGLKNRSRKPKKPKADDPNSKMVISCPDF
DSRITEAERESGNQEIVDSILMRFDAVRRRLCQLNYRKDKILTASTNCMNLGVRTNMTRRIGP
IPGVQVGDIFYYWCEMCLVGLHRNTAGGIDSLLAKESGVDGPAATSVVTSGKYDNETEDLET
LIYSGHGGKPCDQVLQRGNRALEASVRRRNEVRVIRGELYNNEKVYIYDGLYLVSDCWQVT
GKSGFKEYRFKLLRKPGQPPGYAIWKLVENLRNHELIDPRQGFILGDLSFGEEGLRVPLVNEV
DEEDKTIPDDFDYIRSQCYSGMTNDVNVDSQSLVQSYIHQNCTCILKNCGQLPYHDNILVCRK
PLIYECGGSCPTRMVETGLKLHLEVFKTSNCGWGLRSWDPIRAGTFICEFTGVSKTKEEVEED
DDYLFDTSRIYHSFRWNYEPELLCEDACEQVSEDANLPTQVLISAKEKGNVGRFMNHNCWP
NVFWQPIEYDDNNGHIYVRIGLFAMKHIPPMTELTYDYGISCVEKTGEDEVIYKGKKICLCGS
VKCRGSFG

>AtKMT1A;4e

MGLVGLHSGTIDMEFIGVEDHGDEEGKQIAVSVISSGKNADKTEDPDSLIFTGFGGTDMYHG
QPCNQKLERLNIPLEAAFRKKSIVRVVRCMKDEKRTNGNIYIYDGTYMITNRWEEEGQNGFI
VFKFKLVREPDQKPAFGIWKSIQNWRNGLSIRPGLILEDLSNGAENLKVCLVNEVDKENGPAL
FRYVTSLIHEVINNIPSMVDRCACGRRSCGSKHVFREKLSVSSSLVISAKKSGNVARFMNHSC
SPNVFWQSIAREQNGLWCLYIGFFAMKHIPPLTELRYDYGKSRGGGKKMCLCRTKKCCGSFG

>GrKMT1A;1a

MVVQSPHSLQSSVPTPNNGTKRVQILVKENGSAVTDNVAGQRRASARLQAAKQKAEMELLA
KRKLELLNEDVGRSTKKVNVDAEKLKPKLQPAQTVIQQLPEISSLPADPKINKKVAKMVERA
AKIAEGLDGTNAPNVAEKSAHMKVKETIRLFNKHYLHFVQEEEKRCGAAKVDKKALKAKK
KANRGSVSEADVKAKAKRPDLKAISKMMERNEVLYPEKRIGSIPGIDVGHRFYSRAEMVAV
GFHSHWLNGIDYMGQSYKKGEYEHYIFPLAVAIVLSGMYEDDLDNAEDVVYTGQGGHDLT
GNKRQIRDQVLERGNLALKNCVDLGVTVRVVRGHECANSYSGKVYTYDGLYKVVHYWAE
KGISGFTVFKYRLRRLEGQPTLTTSQVHFTYGRVPQCPSEIRGLVCEDISGGQEVVPIPATNLV
DDPPVAPTGYQYSKSMKFARNIKLPANAAGCDCKGLCWDPKTCACARLNGSDFPYVHRDG

GRLIEAKHVVFECGPKCGCDPSCVNRTSQRGLKYRLEVFCTPKKGWAVRSWDFIPAGAPVCE
YIGVLTRTEELDNVSENNYIFDIDCLQTMRGLGGRERRQLDASLPMIQNMDKIDEQRSDSVPE
FCIDAASIGNVARFINHSCEPNLFIQCVLSAHQDIKLARVMLFAADSIPPLQELTYDYGYALDS
VHGPDGKVKKMACYCGAEGCRKRLF

>GrKMT1A;1b

MEIQRRVSPRFQNLPNGSNSNIEKIWNATKSRKTLNTRGSPRFQNLLNVGNSNIEKIRNATDSG
KKSNTRASPNSNIVMIRNETDSAKISNTRVSPRFQNLPKFVKMRDATDTRKKSNTRVSPRFQN
LPNGGNSNNEKKRDATLSTKTSNTRASPRFQNLPNIEKIWGATDSRTTSNTRVSPRFQNLPNG
GNSNNEKKRDATPSTKTSNTRVSPRFQNLPKFEKIWDATDPRKTSNTRVSPRFQNLPNGDNS
NTEKLRDATLSIKTSNTRVSPRFQNVPNVDNSNIEKTWDPTDSRKTLNTRVSPRFQTLPNGDN
SNIEKIQNVPDSRKTPNTRVSPRLQSIPLEKRPFYGSSQKRKTMNDSQDEIMVKKHKVGNTKL
ECLSNGYVTVENGEKDVADLQETGSKGGNYGDDLTSIHGISTTMAVKDKLRLFNKYFLHFSK
AEDARCYRVNGSASDHEIANGKIKDKEEGCEGHVKQAKRSKHKGCVTKRPDLKAISEMLNK
NEVLCHERYFGDLPGIEVGHRFYSRAEMVAVGLHKLLLKRIDYIGKPYVKSEYNGYTFPLAA
AIVMSGQYEDDFGNREEIVYTGEGEKDIPGKKRQFRDQVMRCGNLALKNNKKQSVPVRVIR
GRKCDDSYSKKVYIYDGLYKVTGYWDEKGVSGFKVFKYRLKRLRGQDNLTSQNQVHFVRG
KVSRVQQELLGLVCKDLSNGQEDKCIPVFNFYNPSLAPTGFKYINSIKVAKNVSIPPDAPGCN
CRGKCTNPRSCSCAQLNGGDFPYVSRDGGRLFEAKDVVFECGPNCGCGPECVNRTSQQGLK
YQLEVYRTKEKGWAVRSLDFIPSGAPVCEYVGILRKNDELEDISENDYIFEIDCWHTMKGIGG
RERRQGDVSLPMSNLVDEVDERTLESEPEPEFCIDASSFGNVARFINHSCDPNLFVQCILSSHH
DVRLARIVLFAADDIPRMQELTYDYNYAIDSVIGPDGKTKQLPCFCGTSECRKRLY

>GrKMT1A;2

MGSLVPFQDLNLCPEPPLPATVNISTSATPSATINRTTTFNFLTPKIEPKQEPFDEPTPTQTIHQQ
ENFLFSVSNSTPDFIPNPETTPPANASSSADDQNALYSEYFRISELFRSAFAKRIQKYGDVEVLD
PDSRAIVPVPEQSQLTPETSPTDGSNLERALSVVVSRKKAGRSNELVRVTNLGIDDQRHFRDL
VRRTRMMYDSLRVLVISEEEKRTGPGNGRRARGDLRAAAVMRDRELWLNRDKRIVGAIPGI
EIGDVFFFRMELCVVGLHGQIQAGIDTLPASQSSSGEPIATSIIISGGYEDDEDSGDSIIYTGQGG
QDKFGKQCMHQKLEGGNLALERSMHYGIEVRVIRGFKYENRVSGKVYVYDGLYKILDCWF
DVGKSGFGVYKFRLSRIEGQPEMGSSIMKFAESLRTKPLSTRPMGYLTLDISMKKERVPIFLY
NDIDNDHDPMYYDYLVNTVFPLNVFGQGSNSTGCDCVSGCTEGCFCAMKNGGDFAYDYGG
ILLRGKPVIFECGNFCQCPPSCRNRVAQHGLRNRLEIFRSRETGWGVRSLDLIQAGAFICEYAG
VVLTRDQAEVFKMNGDTLIYPNRFSERWAEWGDLSRIFPEYVCPSYPSIPPLDFAMDVSRMR
NAACYISHSSSPNVLVQCVLYDHNNLMFPHLMLFAMENIPPMRELSIDYGVADEWTGKLSIC
N

>GrKMT1A;3a

MKLKSRRTTLSRFRQFLSSSSYTLVYKWPYGLKIIHEISMVASSSHSEGRSGRVPTENGHVAP
APRFKQRKVSAVRDFPPGCGRVAAPITRPSEQAQGLVVSESENPASLPDSVERVQCETKVSEE
SENATSSVNEVSGSQKDLPEENAASITRPSEQAQGQAVSESENPASLPDCVDKVQCETKFSGE
SRIATSSVNEVSGSEKDLPEENAAISEEEVPENGVEFHPGESSLARNYRPRKGVTVVRHFPPFC
GRNAPPLSDEERMKWLTSLKNKGFNLDKFVNKEKPLEKTLCTDVRQAIEDVQEIYALDGKV
EGCSPRLPVEEIQTKPEESASEKVGKRGAYEEASSRNDVEEDVENTNENNIKPSCESFPNEPDS
NSEKVTETRDDSRGLEENPIRDIVVYEGGDSFEKKLSVSSAFEGQLMEEDCIFVNEEGNSFEK
KLSDLSAFGDQLLEENHGSQEILSTSPHRQGKISFKADLAGGSLKRKKRSNATTKSSSRKRAH
QGQVIVWNKEDSLEQDELHKNDNFAWRSYSYDVSLPPCPSRSDHDNDEMTTRNKVRETLRL

FQAIFRKLLQEEESKMKEKGKAPKRIDILAAKILKEKGKYVNTGKQIIGPVPGIEVGDEFQYFV
ELNLVGLHRQSQGGIDYVKQGDKIIATSIIASGGYENDLDNSDVLCYMGQGGNVMQKGKQP
EDQKLERGNLALANSRFVKNPVRVIRGETKSSSTLLEARGKTYVYDGLYSVEEFKQEPGPHG
KLVYKYKLVRIPGQPELTWKVVKKSKVREGLCVHDISLGKEVIPISAINTIDSEKPPPFEYVCH
MIYPDWCRPIPPKGCGCTKGCSELGKCSCVMKNEGEIPYNHNGAIVEAKPLVYECGPNCNCP
PSCYNRVSQHGIKIQLEIFKTESRGWGVRSLNSISSGTFICEYAGELLEDREAEERKGNDEYLF
DIGNNIDSSLWDGLSNLLPDAHSSSCQVVHESGFTIDAARCGNIGRFINHSCSPNLYAQNVLY
DHEDTRIPHIMFFAAENIPPLQELTYHYNYMIDQVRDENGNIKKKICYCGSSECTGRLY

>GrKMT1A;3b

MVVSDDMLHIKTSKLASGTHSEKCSGRVSTENGHFASTSKYKHAKVYAIRDFPPDCGWLDEP
IDRPSEQKDLPQENVATSEIEVFPQGGVKSYPDQSSRRRILGRKCYTQEEVTVVRDFPPFCGRN
APPLNEEERMKWLASLKNKGIKLVTAVNEEKLSEKTRCTDGKQVIEAVQDVIALEGRAPRLS
AEEIQSKPEEPPSEKMRKQGACEASSRNDVKECMYNIIGNSTKSPCGASPKEFHSKLKKVIEK
RDGNIRVSEENPICNIALKAEDKDFETNLSDSSAFENQLLEEDDESQEVLSDRSIVPGLIASPWL
HAKVTCNPGLAGGSSKRKRMNSFALLSRANTKNNSPTKRAHQGLSQVVIWNKKDSLQQDV
LYTNDSSAQRSYGNNVSLPPCRTSTGRANDAMTSRNKVREILRLFQDICQKLLHEEESKINGE
GKTLKRVDFLAAKILKEKMEHVNTGKQIIGSVPGVEVGDEFQYFVELNIVGLHRPSQGGIDYI
KRGERTIATSIIASGAYEDELDNSDILTYMGQGGNVMQKGKQPEDQRLERGNLALANSRFVK
NPVRVIRGETRSSGLSEGRGKTYVYDGLYVVEEFKQEPGPHGKLVYKFKLVRIPGQPEIAWK
VVRKSKVREGLCVQDISQGKEIIPICAINTRDSERPPLFLYVPHMIYPDWCRRTPSKGCDCING
CSQSVKCSCVMRNGGQIPYNHNGAIVETKPLVYECGPTCKCPASCYNRVSQHGIKFQFEIFKT
KSTGWGVRSLNSIPSGSFICEYAGELLEDREAEKRTGKDEYLFDIGNKYSDSSLWDDLSTLIH
DSRSSFRQVVPECGFTIDAARFGNMGRFINHSCSPNLYAQNVLYDHDDKRIPHIMLFAAENIPP
LQELTYHYNYMIDQVHDESGDIRKKVCCCGSSKCTGRLY

>GrKMT1A;3c

MLKIYQRRFKRISLVTCCNDEQCQVGVSAATNVYKYTGFEESYSSMNIKGEQCAVDIPFANNI
YACIESKQEGVERANVLMNIGDDPCEAVVTSFDDYQLALKDEKNIFQAAFNDDEDYVKEHD
SQAAFSDDVVSVKTTLAEEARSLMVSDERCSDSQVAFNDDEDCVKEHDSLAAFSDGEESVK
TTLAEEKNSQVDLSNYEVFSNNELINDCDKVKQIINHFRHVYNKLLQVKSGKLRSGLAVEAAI
ILQNQHKWIKRNKQFGSISGVEIGDYFFWRAELNIIGLHCRNVHGIDFMKMDGKNLAISVVDS
GRYDNVFESNNEEFPDTLVYLGEGSNPKVQSKKSIEDQKLKGGNLALKNSVEAKNPVRVIRK
IFFKRGKVEKRKYIYDGLYFVDSYRQEIASSGKLVFKFFLKRFPSQPKLDWRKLVRELDCMSY
NSEGKERSPICVVNALDDEKPLITDDISQGKERIPIRAINALDDEKLPIFNYVTSVTYPESYCPS
MINDGCDCIDGCSDSEDCPCIVKNGGSTYDYEERLFEAKPLIVECGPSCKCFTSCLNRVSQRGI
RLPLEVFKTKAKGWGVRSRSFIRRGCFICEYTGEILRDNEGEQRIGNDEYLFDIGVTYGDHSLR
DANSLGSFEGNECFTIDAARVGNVGRFINHSCSPNLFPQGVLFDHDNKRMPHIMLFAMEDIPP
LNELTYDYNYEKGGVCDANGNIKIKHCYCDSSDCLGRMY

>GrKMT1A;4a

MEDDDGQDYVDKSRVLNVQPLRCLAPIFPSTASFPSFPHPQAASPFMCPPPAGHFPPGFASFYP
FSGYPDSHNMQTPMPFGFNGSIPAVPINSFRSGANGDAGSSGRSSRNPGPSQYEEEDDYSDWV
HVNEGEDSSKAATKKKRIPKRVRAGSGQDINVASSDVDVDAMVDKIVESFNLMDFDAFKRA
DGDKDSVGYIRMIYDLLRRKLSQIEDSKESTPGVTRRPDLRAGTILMNKGIRTNVKKRIGVVP
GVEVGDIFFFRMEMCLVGLHAPIMAGIDYMGLKVSQDEEPVAVSIVSSGGYEDNSEDADVLV
YSGQGGNINNKGMEITDQKLERGNLALEKSLHRGNEVRVIRGVKDIANPTGKIYVYDGLYKI

QESWVDKGKSGCNVFKYKLVRLSGQPEAYTVWKSVQQWKDGSTGRVGVISHDLTSGAESIP
VSLVNDVDDEKGPSNFTYYPGLKYLKPVNSNESSTGCGCHGGCLAGNSSCPCIQRNGGNLPY
TTNGVLVSQKPLIHECGSSCLCPPSCKNRVCQSGLKIRLEVFKTKDKGWGLRSWDPIRSGAFI
CEYAGEVIDITSAEELRCVNDDDYIFDATRTNQSGEGFLNASNETLKIPFPLIINAKHAGNVAR
FMNHSCSPNVFWQPVLRENSKECDLHIVFYAYRHIPPMAELTYSYGIVPPERADERRKKCLC
GSAKCLGYFY

>GrKMT1A;4b

MEGGLSGNTVVPNSNDKSTVLDVKPLRMLIPQFPDTSEGPPFVCVPPNGPFPSGFSPFFPFSGP
QGSQSTPDLNQNGLISTILPIRSFRVENASPMDAAHGTHKQKLVASSSVKKKAKVDKGSKLSF
TTPINFDPGISLSERDDGNRELVENVLSRFDALRRKLSQMEDAVELHSGIIKRSDLKAGNMMM
SKGVRTNMKKRIGAVPGVEIGDIFFFRMELCLVGLHAQSMAGIDYIKGELEGELVALSIVSSG
GYDNDAEDPDILIYSGQGGNAGRDKEASDQKLERGNLALEKSLHRGNEVRVIRGFKDAIFQA
SKVYVYDGLYKIQESWMEKGKSGCNTFKYKLVRIPGQTGAFATWKSILKWKEGVSSRVGIIL
PDLTSGAEITPVALVNEVDDEKGPACFTYLATAKYLKSFNLVQTTLGCNCHDACRPGNSNCS
CIKKNGGDFPYIANGVLACRKPLIYECGPSCPCIRNCKNRVSQGGLKFHLEVFKTSDRGWGLR
SWDSIRAGAFICEYAGEVFDSDKARQDDGDGEINEYIFYTNRLYEPFKWNYEPESVGEASSDA
TEDYDIPSPLTISAKNNGNVARFMNHSCTPNVFWQPIVYEHNNGAYLHICFFAKKRIPPMTEL
TYDYGTPCQNETEGDNTTSGRKVCLCGSPKCRGYFY

>GrKMT1A;4c

MEGGLGGNTGPTNPSDKSKVLDVKPLRTLVPLFPEPPEGPPFVCVPPNGPFLSGFSPFFPFSGQ
QVSQPTPDLNQNYLNSTAVPLRSVRAEPAPASGNGSHKHKSAGPSSVKKKAKRRKDSESVLT
SLMNFNPGISLSERDDGNRQLVESVLLRFDALRRKLSQMEDAKELHSSIIKRSDLKAANMML
TKGVRTNMKKRIGVVPGVEIGDIFFFRMELSLVGLHFPSMAGIDYMAIKSGDLEGERVALSIV
SSGGYDDDAEDPDVLVYSGQGGSASRDKEASDQQLVRGNLALERSFHRGNEVRVIRGLKDN
VHQMSKVYVYDGLYKIQESWMEKGKSGCNMFKYKLGRIPGQPAAFATWKSIQKWKEGLPS
RAGLIIPDLTSGAESTPVSLVNEVDDEKGPAYFTYSPTIKYSKPFKLVQPSYACSCHDACQPGN
SNCSCIQKNGGDFPYTTNGVLACRKPMIYECGPTCPCIRNCKNRVIQTGLKAHLEVFKTRDRG
WGLRSWDPIRAGTFICEYAGEVIDETKARQESGDGESEYIFHTNRLYESFKWNFEAESSEDFDI
PSPVIISSKNSGNVARFMNHSCTPNVFWQPVMYEHNNEAFLHIAFFAKKHIPPMTELTFDYGIP
QSGETQADNPPANGRKKCLCGSPKCRGYFY

>GrKMT1A;4d

MEGGVAGNSVPPNSFDKSKVLDVKPLRSLLPVFPEAPNGPPFVCAPPNGPFPTGFSPFFPFSGP
QGSPLTPGLNQNLFNSTAMPIRSFRAEPPPASNGENVQSSNKRKSVGPSSVKKKVKRSNDSEL
ALAALTNFKPGISAAEKDDGNRELVENVLMRFEALRRKLSQMEDAKESHSDIFKRANLKAG
NIMFTKGVRTNGKKRIGVVPGVEIGDIFFFRMELILIGLHSQSMAGIDFMPMKADIEGERVAISI
VSSGGYEDNAEDPDVLVYTGQGGNASADKEASDQKLVRGNLALERSLHRANEVRVIRGFKD
ATHQTSKVYVYDGLYKVQESWMEKGKTGCNMFKYKLVRLPGQTGAFSTWKSIRKWKEDPS
SRDGLILPDLTSGAESIPVSLVNEVDDEKGPAYFTYVSTVKYPKSFKLVQPSYGCNCRDACQA
GNSNCSCIQKNGGDFPYITTGILACRMPMIFECGSSCPCFRNCKNRVLQTGFKVHFEVFKTRD
KGWGLRSWDPIRAGTFICEYAGEVIEKIKEKADGDDGENNDYVFNTNRVYESFKWNHETES
AEERSDTSEKFDIPSPLIISSKNSGNIARFMNHSCSPNVFWQPIMYENNNEAFLHIAFFAKKHIP
PMTELTYDYGIPRSDETESNNMEHGKKKCLCGSPKCRGYFY

>OsKMT1A;1

MEVMDSVAVMEVSPVPKPPLEAALALRRSVRCLNRTRRPTYVEQEEPKESAGRRRGGKRKR
EEEKKEPVAQHAMKPVRMGDAASERKPSSEGKPMPAIAAEPVSCAGFARPAAEDDVLGNGK
SAKLRVKETLRAFTSHYLHLVQEEQKRAQAVLQEGQKRPSKRPDLKAITKMQESNAVLYPE
KIIGELPGVDVGDQFYSRAEMVVLGIHSHWLNGIDYMGMKYQGKEEYANLTFPLATCIVMS
GIYEDDLDKADEIIYTGQGGNDLLGNHRQIGSQQLQRGNLALKNSKDNGNPIRVIRGHISKNS
YTGKVYTYDGLYKVVDDWVQNGVQGHVVFKYKLKRLEGQPSLTTSEVRFTRAEAPTTISEL
PGLVCDDISGGQENLPIPATNLVDDPPVPPTGFVYSKSLKIPKGIKIPSYCNGCDCEGDCANNK
NCSCAQRNGSDLPYVSHKNIGRLVEPKAIVFECGANCSCNNNCVNRTSQKGLQYRLEVFKTA
SKGWGVRTWDTILPGAPICEYTGVLRRTEEVDGLLQNNYIFDIDCLQTMKGLDGREKRAGSD
MHLPSLHAENDSDPPAPEYCIDAGSIGNFARFINHSCEPNLFVQCVLSSHNDVKLAKVTLFAA
DTILPLQELSYDYGYVLDSVVGPDGNIVKLPCFCGAPYCRK
RLY

>OsKMT1A;2a

MEMDTSPSSSAPSSPAASSDSIDLNFLPFLKREPKSEPASPERGPLPLPAAAPPPPPPPPPPPPPPQ
VQAATVATPVPATPDLSAAAVMTPLQSLPPNPEEETLLAEYYRLATLYLSSAGAAGVIVPAA
APEASAGAVAQPGSGSGAKKRRPRSSELVRVSSLSVQDQIYFRDLVRRARITFESLRGILLRDD
ERAEVLGLTGVPGFGAVDRRRVRADLRAAALMGDRDLWLNRDRRIVGPIPGISVGDAFFFR
MELCVLGLHGQVQAGIDFVTAGQSSSGEPIATSIIVSGGYEDDDDRGDVLVYTGHGGRDPNL
HKHCVDQKLEGGNLALERSMAYGIEIRVIRAVKSKRSPVGKVYFYDGLYKVVDYWLDRGKS
GFGVYKYKMLRIEGQESMGSVNFRLAEQLKVNALTFRPTGYLGFDISMGREIMPVALYNDV
DDDRDPLLFEYLARPIFPSSAVQGKFAEGGGGCECTENCSIGCYCAQRNGGEFAYDKLGALL
RGKPLVYECGPYCRCPPSCPNRVSQKGLRNRLEVFRSRETGWGVRSLDLIKAGTFICEFSGIVL
THQQSEIMAANGDCLVRPSRFPPRWLDWGDVSDVYPEYVAPNNPAVPDLKFSIDVSRARNV
ACYFSHSCSPNVFVQFVLFDHYNAAYPHLMIFAMENIPPLRELSIDYGMIDEWVGKLTM

>OsKMT1A;2b

MASPPPPPRLLTPKPDPDAPLPPLPYPDPNLVQSMLFSAQSPQAQPQAPPPHIQPPASASAEAPS
GDEKNKKKKKRARASQEMVRITNLSIADHLHYRSLVRRARLTFEALRAIYQRQDLATAGGIR
NRFDLRASSKMLSKGLWMHRDIRTVGSIPGLLVGDSFFYRAELCVLGLHTAPQAGIGYIPASI
VDQGHPVATSIVSSGGYLDDEDSGDVLVYSGSGGRLRNRLDHSADQTLQRGNLALHYSCHY
GIEVRVIRGHACDHSPSSKVYVYDGLYRVVTSTFGPGKSGRDVCKFKLVRIPGQDDLGSKAW
HTAAELKDALDSKIRPPKYISLDIAKGKEPFRVPLYNKLDDDRSPLFYDYIACPDFPTTQQLLK
RQTQRGCHCAELCGSRCSCERKNRGADGPVYTSDGILLRGRPLVYECGPLCGCPMTCPNRVT
QQGMKHRLEVFRSKETGWGVRTLDLIQPGAFICEYAGDVLSLDSHSGDAPLPPMEDGSSIIDP
TKFPERWREWGDASVVYPDRVPHFPLFAGARYRLDVSQRRNVACYISHSCSPNVFLQYVIRG
NEDESYPHMMVFAMETIPPMRDLSIDYGLD

>OsKMT1A;3a

MDGPTLELSEMMLHAAQPWRSRCTQRDVRPGAVPPRPVAADGRGEGTSTVRGRVLEGTTR
GGGRGGGMEREREVVAPARNAVAVAGDLATHGGERVAGPLVAKEKRNGGGELGTKRGLE
KRAPLPPPKRRVVSAKRQFPPDFGRDSAVPLGRGRGRGGGVRPSDGAPARAVLGEKVASAG
NGDSMANVHHHAVMDTVLMKSSHASDENLVAFKVGSPENGAEGAARGKGAHNGELLGKR
EVLAQAVNLLPMRRTVSATHRFTAGCGRDAAAPLARREEGKVGSGLEVMPVDVGGGVSKE
VMATDGSKHSVNQCTANIVGAVGVLDGTVQYQELEEGEVADEAYCDVESQKVVGCDSFDD
SAGERHEGVVPVTFAVTEVLTSHAYDEMMQIKALQEGGSDAAQETEHDLPMGGKCETILPD

ASPKCSFGGPSNEIVHGKRVLGSHGMKGEVPSLAIEDHGGIAQIDQELEDVEMTTGEYRVQD
AQIATHVIPHESTTGRHEGGLCASAAAEDVKVMNKYKGTLPKGAAKSSMNIATGVFGDGIM
RSKILSTARKVVKPPVRASHKPPLNTLHRPFSTNSASFGHKKLKVKRPDQSKDIPMKIASTSGL
AGKDNLIDEKALSLEDDDILKALVVHDGKLEVYLNVPSCVQLHRQHGSGNADDRSKIRMLC
RRFQFICRALLHAVEQGSLKIRRVDLAADKIIRKLPGFTKPGPTVGNVNGVEVGDEFMYRVEL
ALVGLHRPYQGGIDTTDYNGVLVAISIVCSGGYPDELSSSGELIYTGSGGKPAGKKKDEDQKL
ERGNLALKNCIETKTPVRVIHGFKGQNREDNSHSRAKQILTFTYDGLYLVVDCWTEGLKGSR
IFKYKLQRIPGQPELPLHIAKGLRRSLSRPGLCIADISQGKEMDPICVINDVSNVHPTSFQYISRI
KYPSWLTKRHPQHHGCDCSDGCIDSTKCFCAVKNGGKIPFNSNGAIVHDKPLIFECGPSCRCH
SSCHNRVSQKGMKIHLEVFRTANKGWGVRSLRSISSGSFICEYVGILLTDKEADKRTNDEYLF
DISHNCDDEDCSKGRPSTISSLNSSGGCSQTMEDVCFTIDASEYGNIGRFINHSCSPNLYAQNV
LWDHDDQRVPHIMFFAAENIPPLQELTYDYNYKIGEVRDLNGRVKVKDCHCGSPQCCGRLY

>OsKMT1A;3b

MSVLNPPPKRRAISAIRKFPRDCGRAASTLAESGASMEELPLEATPISVATGGASMEDSLARTP
ISVQGASLVCGLDHSSEAIDGKTIEDDESSKVENRIQEFQVATNVALDDFEGAKNGSTHPNDSI
AKPSPSHGFVERVNGKGSQQEKKLVARSAGDGKMVSKYEERLQKGTPETRMRDLVDVKAK
KKILKSDKMNGALQNDARSSGDGKMKTKASSTQRGVVRSDMSLKQGDIARKVDATGKCKG
GVNSLIKEATSGKHATTNGIEENDDRDLVSDRIIVQALMAPDKCPWTRRRKSIGGSSESRTPK
LKKKFGRPRKELKDTTPREEVSPEVASCKAIKHEAIEDKEDSYFEDEGNSKASYSDVEGNSKE
LVRGGKALVVCGGKKELCVTLPPSAPFGTDPRSKIRNLLIKFHAACRKLVQVEEQHKGNIGRI
DIEAGKALKQNGFIKPGPIVGNVAGVEVGDEFNFRIELSFVGLHRPYQGGIDSTKVNGILVAISI
VASGGYHDELSSSDELIYTGSGGKAIGNKAAGDQKLERGNLALKNSIETKTPVRVIHGFKGHS
KGEASHSKSKQISTYIYDGLYMVVDYWKEGPEGSMVYKYKLQRIPGQPELALHIIKATRKSK
VREGVCVPDISQGRERIPIPAINTIDDTQPTAFKYTTEVIYPHSYAKEPLKGCDCTNGCSDSNRC
ACAVKNGGEIPFNSNGAIVEAKPLVYECGPSCRCPPTCHNRVSQHGIKIPLEIFKTGNKGWGV
RSLSSISSGSFVCEYAGEVLQENGDEHVETDEYLFDIGHHYHDEVWEDPKFEGILGLESSTSKT
TEDTEGSKTTEDTEGSTIDASKCSNVGRFINHSCSPNLYAQNVLWDHDDMKKPHIMFFATENI
PPLQELTYDYNYGKVEDKNGKEKVKPCFCGSPDCSRRLY

>OsKMT1A;3c

MGIPEVVVPPRAAGPRRYKGLVPWRFQPGFVRPPPVKPPAAAAAVAGGGVAGTPGGKGRGL
GASGEGVGSSGGRGDPQSRRCTRSASAKGSGDARSVEEGGPRVAGDDGGSGKSGVAAEGSG
FEGLRNGRGGGVGTAAAEDCGLEKSNPDGIVGDADVHLESGSDARDGECVSEGLKKPCVNN
SNGSSAADCAPKVKKGNDSGNGGADECNAAAKSSNLACPGNNGDETNRKGRKVVLPWRFQ
VGFKRSFSKAFCSDSESSGPSGTQFYRAQDSSTPCTPATRSSVRCYASAHSGVRVSAMRDFSV
KGEKETSTPYKKSKTGMDGPSQGMPKNGVVLARENIMGSLQNFRLIYRDLLDEEEEKSTEAV
IRPDLQAYRIFRERFITDCDEKKYIGNVPGIKVGDIFHLRVELCVVGLHRPHRVGVDHIKQEDG
TCIAVSIVSYAQSSDIKNNLDVLVYSGAMTAIANQKIEGTNLALKKSMDTNTPVRVIHGFVTH
LNGNCQRKKIPTYIYGGLYIVEKYWREKEGNDRYVYMFRLRRMAGQKHIDIQDILNSGQAES
YGGIIIKDISRGLEKIPVSVVNSISDEYPMPYRYIAHLQYPRNYQPAPPAGCGCVGGCSDSKRC
ACAVKNGGEIPFNDKGRILEAKPLVYECGPSCKCPPTCHNRVGQHGLRFRLQVFKTKLMGW
GVRTLDFIPSGSFVCEYIGEVLEDEEAQKRSTDEYLFAIGHNYYDEALWEGLSRSIPSLQKGPD
KDEEAGFAVDASKMGNFAKFINHSCTPNLYAQNVLYDHDDKSVPHIMFFACEDIPPRQELSY
HYNYTIDQVHDANGNIKKKKCLCGSIECDGWLY

>OsKMT1A;4a

MAGTRQTTSVPMDNAAVVDAKPLRTLTPMFPAALGLHTFTAKENSSSIVCITPFGPYAGGTE
QAMPASIPPMFASPAAPADPNQRQPYAVHLNGAAPANGTANNTGVIPDLQIAVAGTVESAKR
KRGRPKRVQDSSVPSAHLVPSAPGGNITAVQTPPSATTDESGKKKRGRPKRVQDVPVLSTPSA
PQVDSTVFQTPASAVNESVTRKRGRPRRVQDGADTSAPPIQSKYNEPVLQTPSAVTLPEDGKR
KRGRPKRVPDGALIPLSHSGVSIDDDSGEIITGKRGRPRKIDVNLLNLPSLFSDDPRESVDNVL
MMFDALRRRLMQLDEVKQGAKQQHNLKAGSIMMSAELRANKNKRIGEVPGVEVGDMFYF
RIEMCLVGLNSQSMSGIDYMSAKFGNEEDPVAISIVSAGVYENTEDDPDVLVYTGQGMSGKD
DQKLERGNLALERSLHRGNQIRVVRSVRDLTCPTGKIYIYDGLYKIREAWVEKGKTGFNVFK
HKLLREPGQPDGIAVWKKTEKWRENPSSRDHVILRDISYGAESKPVCLVNEVDDEKGPSHFN
YTTKLNYRNSLSSMRKMQGCNCASVCLPGDNNCSCTHRNAGDLPYSASGILVSRMPMLYEC
NDSCTCSHNCRNRVVQKGSQIHFEVFKTGDRGWGLRSWDPIRAGTFICEYAGEVIDRNSIIGE
DDYIFETPSSEQNLRWNYAPELLGEPSLSDSSETPKQLPIIISAKRTGNIARFMNHSCSPNVFWQ
PVLYDHGDEGYPHIAFFAIKHIPPMTELTYDYGQSQGNVQLGINSGCRKSKNCLCWSRKCRG
SFG

>OsKMT1A;4b

MESNQHKASDPQDSMVHLDLDEDKIMVTSALPCPSMSVGKSVMRKRGRPSRHARGTSLSSV
TPEGCKKMEGRSYNLRSDSTILLRNSCLLIADGSTKQKRSWGLDKDDLHIPFFQISDNPREAV
DDILMTFGGLHRRIMQLIDVKMASKQLVFQALNLMRKVGYHVNKDKRVGEVPGVKIGDIFY
SRIEMLLVGLHSNINRGIEFMSGAFINKEDKIATCIVSSGMYENGDDDPYTLVYNGQGKVHHK
LERGNYSLNQSFIRRNHIRLIRSEPNPLVRLGSKEKIYIYDGLYKIEEKYRQTTKSRSNLKFNKL
VRELGQPNGIVVWKNTQKWRENPSCRDHVIMPDMSNGAEIARVCVVNNIDSEDAPNNFTYS
TKLDNGNHMVSANKMCVCKCTSSCLGEDNCSCLKTNGSYLPYNSSGILVCRKTMIYECNDS
CACTINCSNRVVQRGSYLHFEVFKTMDRGWGLRSWDPIPAGAFVCEYVGVVIDKDSLVEED
EYIFEVTRPEHNLKWNYLPELIGEPSFYDMNDTFKKLCLVFSVNKLPTIISAKQTGNVARFMN
HSCSPNVFYQPVMYDHGDEGYPHIAFFAIKNIPPMTELTYDYGQSNGSGCRRPKICICQSHMC
KGTFG

>OsKMT1A;4c

MDRASNFIPGPYQELVDAKPIRSLAPMFPAPLGINVNQSSTPPLVCVTPVGQFPVGFGSGILPTF
GSTTAFTTTANGVSYTSYTNNGAIDATPISAYKTRPGIVSLDGDEPYSGSASGRKSKRSSGSAA
DGSNGVKFKRPKPVYKNFVAGKELAFLPPSSSDPREVVEAVHMTFEALRRRHLQLDEIQETS
KRADLKAGAIMMASNIRANVGKRVGLVPGVEIGDIFYFRMELCIIGLHAPSMGGIDYMSAKF
GSDEDSVAICIVAAGGYENVDDDTDTLVYSGSGGNSRNSEERHDQKLERGNLALERSLHRKN
EIRVVRGFRDPFCLTGKIYIYDGLYKIQESWKERTKSGINCFKYKLLREPGQPDGAALWKMT
QGWIDNPASRGRVILPDLSSAAEALPVCLVNEVDHEKGPGHFTYASQVKYLRPLSSMKPLQG
CGCQSVCLPGDPNCACGQHNGGDLPYSSSGLLACRKPIIYECGDACHCTTNCRNRVTQKGVR
FHFEVFRTANRGWGLRCWDPIRAGAFICEYTGEVIDELKVNLDDSEDDYIFQTVCPGEKTLKF
NFGPELIGEESTYVSADEFEPLPIKISAKKMGNVSRFMNHSCSPNVFWQPVQHDHGDDSHPHI
MFFALKHIPPMTELTFDYGVAGSESSGSRRTKNCFCGSSNCRGVF

>OsKMT1A;4d

MNRESNFMPTPDQDVLEVKPLRTLAPMFPAPLGIDVLNRLTAPPLVFVAPAGQFPGGFGSLNI
PAVRSFAAFGGQDASGGKTAGGGDQDASGGKTAAGGDQDAGRGETAAFGGQETVRGEFVA
NGTPNVGASATGPIDATPISACKSTQPSVISLDDDDNDDDEPYGGNQTSASGRKIKRPSHLKG
YNVSDGLGTDSSNGTKKRPKTSNRKAATDNEISLMPPSSDPREVVEVLLMTFEALRRRHLQL

DETQETSKRADLKAGAIMLASNLRANIGKRIGAVPGVEVGDIFYFRMELCIIGLHAPSMGGID
YMNKFGDEDDSVAICIVAAGVYENDDDDTDTLVYSGSGGISRNSEEKQDQKLERGNLALERS
LSRKNVIRVVRGYKDPACLTGKVYIYDGLYKIHESWKERTKTGINCFKYKLQREPGQPDAVA
IWKMCQRWVENPAARGKVLHPDLSSGAENLPVCLINDVNSEKGPGHFNYITQVKYLKPLRS
MKPFQGCRCTSVCLPGDTSCDCAQHNGGDLPYSSSGLLVCRKLMVYECGESCRCSINCRNRV
AQKGVRIHLEVFRTTNRGWGLRSWDPIRAGSFICEYVGEVVDDTKVNLDGEDDYLFRTVCP
GEKTLKWNYGPELIGEHSINISADTFEPLPIKISAMKMGNVARFMNHSCNPNTFWQPVQFDH
GEDGYPHIMFFALKHIPPMTELTYDYGDIGCESRGVGSRAKNCLCGSSNCRGFFS

>OsKMT1A;4e

MENSEDEAENGKLPLELEPLRSLAPKFPTILGYDVETQSTNPLLVYATPFITTASRSPSFIFSALT
KVTNESSDQDYKPFCRQKKPTLPKRAKRPQQAEKSNDANIKRRSIRRSLDNEFNLCSSSSDNP
KESVEGILMMFDSLRRRVLQLDEKEDASRRADLKAGTLMMQNNLRINNHKMIGHVPGVEV
GDIFFFRIEMCIIGLHAPAMGGIDYISSKNKDETLAVCIISSGGYENDDDDTDILVYTGQGGNSR
HKEKHDQKLERGNLALMNSKIKKNQIRVVRSAQDPFCNSGKIYIYDGLYRIEDTWTDTAKNG
FNVFKYKLRRDPGQPDGISLWKMTEKWKANPATREKAILLDLSSKVEHLPVCLVNDVDDEK
GPSHFNYVAGVKYLRPLRKTKPLQCCKCPSVCLPGDPNCSCAQQNGGDLPYSATGLLAKHTP
MVYECSSNCQCSHNCRNRITQKGIKLNFEVFWTGDRGWGLRSWDPIRAGTFICEYAGEVIDE
TKMDIDVEEDKYTFCASCPGDKALSWNLGAELLEEKSTAVTTKNFKKLPIIIRANNEGNVARF
LNHSCSPNLLWQAVQYDHGDDSYPHIMFFAMEHIPPMTELTYDYGTRGAPPGFEGKPFKAC
KLKSCLCGSKHCRGLF

>OsKMT1A;4f

MENSEDEAESDKLPLDLEPLRSLAPKFPTILGYDVETQSTDPLLVYATPSIPCSSSEQPQEAPAS
FSLPLPKSPVPIKATPISAAFPTPQHEDESSDQDYKPFCKNKKPAMPKRAKRPQQAEKSNDANI
KRRSIRRNLDNEFNLCSSSSDNPKESVEGILMMFDSLRRRVLQLDEKEDASRRADLKAGTLM
MQNNLRINNHKMIGHVPGVEVGDIFFFRIEMCIVGLHAPAMGGIDYISSKNKDETLAVCIISSG
GYENDDDDTDILVYTGQGGNSRHKEKHDQKLERGNLALMNSKSKKNQIRVVRSAQDPFCNS
GKIYIYDGLYRIEDTWTDTAKNGFNVFKYKLRRDPGQPDGISLWKMTEKWKANPATREKAI
LLDLSSKVEHLPVCLVNDVDDEKGPSHFNYVAGVKYLRPLRKTKPLQCCKCPSVCLPGDPNC
SCAQQNGGDLPYSATGLLAKHTPMVYECSSNCQCSHNCRNRITQKGIKLNFEVFWTGDRGW
GLRSWDPIRAGTFICEYAGEVIDETKMDIDVEEDKYTFRASCPGNKALSWNLGEELLEEKSTA
VITKNFKKLPIIIRANNEGNVARFLNHSCSPNLLWQAVQYDHGDDSYPHIMFFAMEHIPPMTE
LTYDYGTRGAPPGFEGKPFKACKLKSCLCGSKHCRGY

KMT1B

>AtKMT1B;1

MISLSGLTSSVESDLDMQQAMLTNKDEKVLKALERTRQLDIPDEKTMPVLMKLLEEAGGNW
SYIKLDNYTALVDAIYSVEDENKQSEGSSNGNRGKNLKVIDSPATLKKTYETRSASSGSSIQV
VQKQPQLSNGDRKRKYKSRIADITKGSESVKIPLVDDVGSEAVPKFTYIPHNIVYQSAYLHVS
LARISDEDCCANCKGNCLSADFPCTCARETSGEYAYTKEGLLKEKFLDTCLKMKKEPDSFPK
VYCKDCPLERDHDKGTYGKCDGHLIRKFIKECWRKCGCDMQCGNRVVQRGIRCQLQVYFT
QEGKGWGLRTLQDLPKGTFICEYIGEILTNTELYDRNVRSSSERHTYPVTLDADWGSEKDLK
DEEALCLDATICGNVARFINHRCEDANMIDIPIEIETPDRHYYHIAFFTLRDVKAMDELTWDY
MIDFNDKSHPVKAFRCCCGSESCRDRKIKGSQGKSIERRKIVSAKKQQGSKEVSKKRK

>AtKMT1B;2a

MDEDEFPLKRRLRSRRGRASSSSSSSSYNNEDLKTQPEEEDEDDGVTELPPLKRYVRRNGER
GLAMTVYNNASPSSSSRLSMEPEEVPPMVLLPAHPMETKVSEASALVILNDEPNIDHKPVISD
TGNCSAPMLEMGKSNIHVQEWDWETKDILNDTTAMDVSPSSAIGESSEHKVAAASVELASST
SGEAKICLSFAPATGETTNLHLPSMEDLRRAMEEKCLKSYKIVHPEFSVLGFMKDMCSCYIDL
AKNSTSQLLETETVCDMSKAGDESGAVGISMPLVVVPECEISGDGWKAISNMKDITAGEENV
EIPWVNEINEKVPSRFRYMPHSFVFQDAPVIFSLSSFSDEQSCSTSCIEDCLASEMSCNCAIGVD
NGFAYTLDGLLKEEFLEARISEARDQRKQVLRFCEECPLERAKKVEILEPCKGHLKRGAIKEC
WFKCGCTKRCGNRVVQRGMHNKLQVFFTPNGKGWGLRTLEKLPKGAFICEYIGEILTIPELY
QRSFEDKPTLPVILDAHWGSEERLEGDKALCLDGMFYGNISRFLNHRCLDANLIEIPVQVETP
DQHYYHLAFFTTRDIEAMEELAWDYGIDFNDNDSLMKPFDCLCGSRFCRNKKRSTKTMQIL
NKA

>AtKMT1B;2b

MAPNLHIKKAFMAMRAMGIEDARVKPVLKNLLALYEKNWELIAEDNYRVLADAIFDSHEDQ
AIQESEEKKADEVKEDEGCAAEVDRGKKKLHESIEDDEDVMAESDRPLKRLRRRGEGGSAL
ASPSLGSPTLEGPSINDEENAPILLPYHPVPIENDHDAGELILTKVEPITNMPLSSIPDSVDRGDS
SMLEIDKSNGHVEEKAGETVSTADGTTNDISPTTVARFSDHKLAATIEEPPALELASSASGEVK
INLSFAPATGGSNPHLPSMEELRRAMEEKCLRSYKILDPNFSVLGFMNDICSCYLDLATNGRD
SANQLPKNLPFVTTNIDALKKSAARMAYTSQASNDVVEICSNEHMRDAENGAVGDSMALVV
VPECQLSADEWRLISSVGDISLGKETVEIPWVNEVNDKVPPVFHYIAQSLVYQDAAVKFSLGN
IRDDQCCSSCCGDCLAPSMACRCATAFNGFAYTVDGLLQEDFLEQCISEARDPRKQMLLYCK
ECPLEKAKKEVILEPCKGHLKRKAIKECWSKCGCMKNCGNRVVQQGIHNKLQVFFTPNGRG
WGLRTLEKLPKGAFVCELAGEILTIPELFQRISDRPTSPVILDAYWGSEDISGDDKALSLEGTH
YGNISRFINHRCLDANLIEIPVHAETTDSHYYHLAFFTTREIDAMEELTWDYGVPFNQDVFPTS
PFHCQCGSDFCRVRKQISKGKNVKKRA

>AtKMT1B;3

MILQRYMDPIWLENSFPLWVQKCNNAVNAESIELLNEEFDNCIKWNEVKSLSESPMQPMLLS
EWKTWKHDIAKWFSISRRGVGEIAQPDSKSVFNSDVQASRKRPKLEIRRAETTNATHMESDT
SPQGLSAIDSEFFSSRGNTNSPETMKEENPVMNTPENGLDLWDGIVVEAGGSQFMKTKETNG
LSHPQDQHINESVLKKPFGSGNKSQQCIAFIESKGRQCVRWANEGDVYCCVHLASRFTTKSM
KNEGSPAVEAPMCGGVTVLGTKCKHRSLPGFLYCKKHRPHTGMVKPDDSSSFLVKRKVSEI
MSTLETNQCQDLVPFGEPEGPSFEKQEPHGATSFTEMFEHCSQEDNLCIGSCSENSYISCSEFST
KHSLYCEQHLPNWLKRARNGKSRIISKEVFVDLLRGCLSREEKLALHQACDIFYKLFKSVLSL

RNSVPMEVQIDWAKTEASRNADAGVGEFLMKLVSNERERLTRIWGFATGADEEDVSLSEYP
NRLLAITNTCDDDDDKEKWSFSGFACAICLDSFVRRKLLEIHVEERHHVQFAEKCMLLQCIPC
GSHFGDKEQLLVHVQAVHPSECKSLTVASECNLTNGEFSQKPEAGSSQIVVSQNNENTSGVH
KFVCKFCGLKFNLLPDLGRHHQAEHMGPSLVGSRGPKKGIRFNTYRMKSGRLSRPNKFKKSL
GAVSYRIRNRAGVNMKRRMQGSKSLGTEGNTEAGVSPPLDDSRNFDGVTDAHCSVVSDILL
SKVQKAKHRPNNLDILSAARSACCRVSVETSLEAKFGDLPDRIYLKAAKLCGEQGVQVQWH
QEGYICSNGCKPVKDPNLLHPLIPRQENDRFGIAVDAGQHSNIELEVDECHCIMEAHHFSKRP
FGNTAVLCKDISFGKESVPICVVDDDLWNSEKPYEMPWECFTYVTNSILHPSMDLVKENLQL
RCSCRSSVCSPVTCDHVYLFGNDFEDARDIYGKSMRCRFPYDGKQRIILEEGYPVYECNKFCG
CSRTCQNRVLQNGIRAKLEVFRTESKGWGLRACEHILRGTFVCEYIGEVLDQQEANKRRNQY
GNGDCSYILDIDANINDIGRLMEEELDYAIDATTHGNISRFINHSCSPNLVNHQVIVESMESPL
AHIGLYASMDIAAGEEITRDYGRRPVPSEQENEHPCHCKATNCRGLLS

>AtKMT1B;4

MQRLRESPPPKTRCLGEASDIIPAADRFLRCANLILPWLNPRELAVVAQTCKTLSLISKSLTIHR
SLDAARSLENISIPFHNSIDSQRYAYFIYTPFQIPASSPPPPRQWWGAAANECGSESRPCFDSVS
ESGRFGVSLVDESGCECERCEEGYCKCLAFAGMEEIANECGSGCGCGSDCSNRVTQKGVSVS
LKIVRDEKKGWCLYADQLIKQGQFICEYAGELLTTDEARRRQNIYDKLRSTQSFASALLVVR
EHLPSGQACLRINIDATRIGNVARFINHSCDGGNLSTVLLRSSGALLPRLCFFAAKDIIAEEELS
FSYGDVSVAGENRDDKLNCSCGSSCCLGTLPCENT

>GrKMT1B;1

MSVRERARKAFQATRALGLPDQEVTPVLEHLLKLFNNNWDLIESEDYRALIDAYFELKENKG
EDNRKNVVGDYGESSRLPKRLCLQDSEGQASPTKGSARQILSPEEHGKPSSVNLQQGATFLN
KKDSSSSGCSNLCKKPQQQPVTCEKNRPLHIINDITKGTENVKISLVGDIGKQELPKFTYMRD
NIIYQDAYVHISLARVADEDCCSGCSGDCLSVSIPCACAHETGGEFAYTTDGQLRDKFLKACI
SMKQDPEGHDSVYCQDCPLERLKNEYKPEKCKGHLVRKFIKECWRKCGCSMQCGNRVVQR
GITCKLQVFWTREGKGWGVKTLQDLPKGTFVCEYVGEILTNTELFERNLKGSGNEKHTYPVT
LDADWGSERVLKDEEALCLDATFCGNVARFINHSLAGCCCEKHCLRTFKKCFLY

>GrKMT1B;2a

MAPNPRVVQAFRTMKEIGISEEKVKPVLKKLLKLYDKNWELIESENYRVLADAIFEEEDSKVS
EPKKSTAHDQEDIDEEGSTPVELVRPLKRARLKNQEGLATCSHTNGSSNVAGTLLKEPKVEE
NEIPPASLQHRSLQSNVGNIRTEILPASPGPVCPQPPSHAPVSPHHSGRDKGKQIVEPRPNYKG
KEPMSPHVASKGKGPERASVALRIKDPAPEPGIIPNNRVSATQALIIPKEEPFTDDMPQDEVPL
AVIQPDSLSGRDLPIGDFSTEKSNWLEPPESLHAAEIAGSSASASGSERHTSCEHATVPDEIPSIL
EIASSQLGEVTISLSYNSALGGPNFQLPSIDELRGLMELRCLQSYKLIDPNFDVIKILTDMCECIS
ELATNSSNQSQEGNEMPALDVLKKSPSRGDAEKNKENGCCEAAMMLNESFDNHCSGNGFV
DNVGRKELVVAPQHHLTSNELRRVLDASDITKGEENFEISWVNEINKEFPTPFQYISDNLVFQ
NAHVSFSLSRIGDERCCPTCLGDCLFSQKPCVCACQAGGKFAYTPEGVIKEDFLEECISMTRD
PQKQCLLNCTECPLERSKADDFPEPCKGHLQRKVIKECWIKCGCNKQCGNRVVQRGVNYKL
QVFLTPDGKGWGLRTLENLPKGAFVCEFVGEILTIPEFYARNREKHTSPVLLDAYWGLKGVP
KDEEALCLDATCYGNVARFINHRCLDANLIEIPVEVETPDLHYYHLAFFTTREVHALEELTWD
YGIDFDDLDHHVKTFQCRCGSKFCRNMKRSIRSKSATR

>GrKMT1B;2b

MARSPRIVQAFRAMKEIGIREDKVKPVLKRLLNLYEKNWELIESENYRVLADAIFDEEESKVS
EPEKDKKCDEDEDTEGGGLVHDELVRRPLKRLRRLGETSCSHRTGSPNVAGTLLKKPKVEDE
PSPASLQQKPLIGKRTEYLPASPGSVSPQPVSPASVSPHHGDRTASPGPVLSQSPSPAHVSPHH
GGMNKGKQTVEPRPLAVLGRPQPNSHSSQMHFSSKGKELMSPNVASNGKGPQRVSLSLHVK
DLGPEPCVIPKKRVVDTHALIIPKEEPFTDDMPQDEVPIAVIHPDPLSGKDLAIREFSTGKSNW
QEPPESLHADEIVGGGASASMSKRHTSCELATVSNEIPSRLEIASSQLGEVKISVSYNNSALGG
QKFQLPSIDELRELMAQRCLRSYKLIDPNFDVIKILNDMCECISELATNSSNQSREGDVMPALD
VLKRSPGRDALDAESNRENGCMLARLLNGSFIVQPSSNGCVDDVGGKELVVLPQHELTLNEL
RWLHDASDITKGEEKVEISWVNEINKDFPPPFQYISENLVFQNARVSFNLYGIGDESCPTCFGD
CLLAEQPCGCACRAGRKFAYSSAGVVEEDFLGECISMTRDPQQQFLLYCTECPLERSKKDEFP
EPCKGHLKRKVIRECWSKCGCHKQCGNRVVQRGLNYKLQVFLTPDEKGWGLRTLEKLPKG
AFVCEFVGEILTISELYARNREKHTCPILLDAYWGLKGGARDEEALCLDATCYGNVARFINHR
CFDANLIEIPVEVETPDLRYYHLAFFTTREVHALEELTWDYGIDFDDLDHPVKAFRCRCGSKF
CRNMKRSTRSKSAIITG

>GrKMT1B;3a

MSRLIEGQARYFIDASKYGNVSRFINHSCSPNLVNHQVLVDSMDCHRAHIGLYASQDISVGEE
LTFDYRYELLPAQGYPCQYGVSTCRGRLY

>GrKMT1B;3b

MYIQFSVYILTRFLLQHLTLAWIELCLDSKVILGDNQCLYAHYTALEIPGCIMEVLPCSGVQY
VADSDCAQLSPEATFTYDRESNCLEQKKQVQVADSRMDDLLLTNEGNQEGRQDEGQGTRV
ELPISEDHHSGSSYYDCQAEGQRLSCGSHDDEYDDLNAQNCCTGPYLTSENSHVLVNTIESES
PINNREGELSLSEPKWLEHDESVALWVKWRGKWQAGIRCARADWPLSTLKAKPTHDRKQY
FVIFFPHTRNYSWADMLLVRSISEFPQPIAYRSHKVGLKMVRDLTVARRYIQQKLAVGMLNII
DQFHVEALIEAARNVVVWKDFAMEASRCNGYSDLGKMLLKLQTMILPCYINAEWLQESLHS
WVQQCQNAHSAESVELLKEELYDAILWNEVKSLGDASVQPTLGSEWKTWKHEVMKWFSTS
HPVSSAGDVNQRSSDGLSNTNIQVSRKRAKLEVRRADTHASMVQSNGSDQTMAVEIDSDFFS
NRDAVDVNLPTPQHCKKEDEREETTPMDTSNSLTDRWNNIVVEARHPEVIHTKNVEITTASE
EVKTTSTLHIQPKEVELTPVNEAVAKKSIDSGSKNRQCTAFIESKGRQCVRWANEGDVYCCV
HLASRFTGSFSKIEATPAVDTPMCEGTTVLGTRCKHRSLYGSSFCKKHRPKSDVNNSSHSPEH
TQKRKHLEIIQSSETTLCRDIVLVGDNESPLQVEPVSVIEADALHRGNSLIEKPEHSGKDHDGT
ELMHCIGLYSNNGFDPCQESPKRHSLYCDKHLPSWLKRARNGKSRIVSREVFVDLLKDCDSL
EQKLHLHQACELFYKLFKSILSLRNPVPVDVQLQWALSEASKDFRVGELLMKLVYSEKERLQ
SLWGFTGDKGTPSSSFMEEPVPLPLAINDSFDDDKTIKCKMCSVEFLDDQQLGTHWMENHKK
EAQRLFRGYACAICLDSFINKKVLESHVQERHHVQFVEQCMLLRCISCGSHFGNTEELWLHV
LSTHPVDFRLSKIALQHNPSAGEEPPLKLELGNSASLENNSENVGSVQKFICRFCGLKFDLLPD
LGRHHQAAHMGPSLASSRPPKKGVRYYAYKLKSGRLSHPRFKKGLGAVSYRIRNRATATMK
KRLQASKLIDAEIISAEPHVMENSNLGRLAEPQCSALAKILFSRTHKTKPRPNNLDILSIARSSC
CKVSLKASLEEKYGMLPECLYLKAAKLCSEHNVQVEWHQEKFVCINGCKPAKDPDFLSPLIP
LPNGFEGCQSADSLDDADEELELDECHYIIDSQHFKKGPMQKASILCDDLSFGKESVPVACVV
DEGLFDSVYISGLSSNEQNARSSMPWENFIYVTNSSLDQSLDLDVESVQLGCTCSNSTCFPETC
DHVYLFDNDYEDARDVFGKPMRGRFPYDDKGRIILEEGYLVYECNRKCSCNIACPNRVLQK
GVRVKLEVFKTENKGWGVRAGEPILSGTFVCEYVGEILGEQEANNRLTRYGRDGCNYMFNI
GSQINDMSRLIEGQARYFIDASKYGNVSRFINHSCSPNLVNHQVLVDSMDCHRAHIGLYASQ

DISVGEELTFDYRYELLPGQGYPCQCGASTCRGRLY
**>GrKMT1B;3c**<br>MKQVQVADNRMNDLLVTNEGNQTGRQDEGQGTRGELPISEEHHSGSSYYDCQAEGQRLFC<br>GSHDDEYDNLNAQSCCTRPYLTSESSHLLVNTIKSESPSNNREGELSLSAPKWLEHDASVAL<br>WVKWRGIWQAGIRCARADWPLSTLKAKPTHDRKQYFVVFFPHTRNYSWADMILVRPINEFP<br>QPIACRSHKVGLKMVRDLTVARRYIQQKLAVGMLNIIDQFHCEALIETARNVVVWKEFAME<br>ASRCNGYSDLGKMLLKLQSMILPRYINADWLQESFHSWVQQCQNAHTAESIELLKEELYNAI<br>LWNEVKSLGDAPVQPTIGSEWKTWKHEVLKWFSMSHPVSTAGDVNQRNSGSPSNTNIQVSR<br>KRPKLDVRRADTHAFQVQSNGSEQTMATEIEYDFFSAVDVNLPTPRLCRKEEEREETTPMDR<br>SNNLTDRWDNIVVEARHSEVIHTKNVEITTASEEVNSTSTLHIQSKEVKLTPVNEAVAKKSID<br>AGSKNRQCVAFIESKGRQCVRWANEGDVYCCVHLASRFTGSFSKIEVTPPVDTPMCEGTTVL<br>GTRCKHRSLYGSSFCKKHRPKSDANNSCHSPENTRKRKHLEIILSSEITFCRDIVLVGDNESPL<br>QVEPVSVIEADALHRGNSLIEKPEHSGKDHDSTELLHCIGLYSNNGFDPCQESPKRHSLYCDK<br>HLPSWLKRARNGKSRIVSREVFVDLLKDCDSLEQKLHLHQACELFYKLFKSIFSLRNPVPVDV<br>QLQWALSEASKDYRVGEFLMKLVYSEKERLQSLWGFTGDKGTPSSSFVEEPVPLPLAINDSF<br>DDDKTIKCKMCSLEFLDDQQLGTHWMENHKKEAQRLFEGYACAICLDSFTNKKVLESHVQE<br>RHHVQFVEQCMLLRCISCGSHFGNTEELWLDVLSAHPVEFRLSKIAQQHNLSAGEEPPPKLEF<br>GNSVENNSENVDSFQKFTCKYCGLKFDLLPDLGRHHQAAHMGPSLASSRPPKKGVRYYAYK<br>LKSGRLSHPRFKKGLGAVSYRIRNRATATMKKCLQASKLIDAEIISAEPLVMETSNLGRLAEP<br>QCSALAKILFSRTHKTKPRPNNLDILSIARSSCCKVSLKASLEEKYGMLPECLYLKAAKLCSEH<br>NVQVEWHQEKFVCINGCKPAKDSDFLPPLIPLPNGFEGHQSADSLEDVDEELELDECHYIIDS<br>QHFKKGPTQKASVLCDDLSFGKESVPVACVVDEGLFDSLNISGLSFNEQNAGPSMPWENFTY<br>VTNSLLDQSLDLDVESMQLGCTCSNSTCYPETCDHVYLFDNDYEDARDIYGKPMRGRFPYD<br>DKGRIILEEGYLVYECNRMCSCNKACPNRVLQKGVRVKLEVFKTENKGWGIRAGEPILSGTF<br>VCEYVGILGKQEANYRLTRYGRNGCNYMFNVDCHINDMSRLIEGQARYFIDASKYGNVSRFI<br>NHSCSPNLVNRQVLVDSMDCHRAHIGLYASQDISVGEELTLDYQYELLPGQGYPCQCGASTC<br>RGRLY
**>GrKMT1B;3d**<br>MWASQVTAAEAPGLLAHPHPLITRRDELAFARVNYGITTQGTPTTGKLEFGQEESRSENVLA<br>LRPLSADYPFRVFSFFLFRFLPSSSTLRFSHASLHRLGVGGGEPILSSTFVCEYVGEILGEQEAN<br>NRLTRYGRDGCNYLFNIGSQINDMSRLIEGQARYFIDASKYGNVSRFINHSCSPNLVNHQVLV<br>DSIDCHRAHIGLYASQDISVGEELTFDYRYELLPGQGYSCRLMFS
**>GrKMT1B;4**<br>MLPPSPKKPPQNKLGLSHAFLQCADLILPWLTPQELANVSLTCKTLHRAAHSITLYRSLDASR<br>SFENFPIPFHNTVDQYPYAYFFYTPSQIIPSSSSSSLNRQFWGPNSSQTLVLPDSGSSYAEMGCT<br>LDSMDLLGGSWVSVMDESGCECERCEKVSEDNVIGCPCMELEGDEGMGIRSECGPSCGCRL<br>ECGNRLSQRGIQVQLKIVKDVRKGWGLYAAQWIQQGQFICEYSGELLTTKEARRRQQIYDKL<br>ASDDHFSSALLVVREHLPSGNACLRINIDATRAGNVARFINHSCDGGNLSTVLVRSSGALLPR<br>LCFFASKDIKEGEELTFSYGEIRVQPKGSKCFCGSFSCLGTLPSEHT
**>OsKMT1B;1**<br>MGSKSNTERARKALEAMKQLGFSKKEATPVLKNLLRLFGNNWEPIEDECYRALADAILDRH<br>QETAADRGCSATRPTPDDDHHPLTLCGASRDVDTETDEPRTKKPRATNSDPQSPPSLTDDQD<br>VPAAISPPSHGASPQFRPQTRASARLRQASPSSVTAAHKRPRQMMDEDFQDSAFLREPKPEPD<br>IDMDAIQGTAATSDCPNAHLGLIDYPLNASSSRVALPLALLPPDQNVPQISGQIDNDVSTQSFS

LCVATGKDFLNLLAFKNSAGNRLQPLFDILAAIQPCSKVNTGEGSSVIDVASSTMGEVKMSLK
CSVDPKFRMPSLEAVFKMVEDKYLHSYKILPPEFSIGSLMNEICQCVVQLGSDHIAEHNTESD
VAGNGRCSQNEPMTGSIPFVKPIACEDGGNRKCKSAGESFIVEDSENSSVAKQQTHLALANL
KPIHDVTDISKGEERVRISVANEFASEKCPPSFYYIRGNLVFQNAYVNISVARIGDEDCCADCF
GNCLSAPIPCACTRETGGEYVYTPEGLVRTPFLDECVSMNRFPEKCHKFFCKSSCPLERSRNE
ASPEPCRGHLARKFIKECWSKCGCNMQCGNRVVQRGITCNLQVFFTGEGKGWGLRTLDELP
KGAFVCEYVGEVLTSTELHERTLQNMNNGRHTYPVLLDADWGSEGVLKDEEALSLDSTFYG
NVGRFINHRCYDANLVEIPVEVETPDHHYYHLAFFTTKKVEAFEELTWDYGIDFGDGKDPVK
AFQCLCGSRYCRGIRHPRKRGKAAAK

>OsKMT1B;2

MAKPNGKEKTGDTGLSMAPPKISKDRFDAAIRAMADIGILKETAAPVLNNLLNLFDYNWVHI
EADNYLALADAIFCDSDPKEGQKRQANETNLDADQSNKKLKTKKRSQNPTSKMHGNDNREF
VEAPPQQGRGTLSARTVNGKKVTRAHLELPSSQLLIKEPHTCPSIAKNTTIVENNSAVLCHGQ
DLQTFEVPVATTCPQVVAPSTRKDARRTSGARHDQKHEGVSGAHERNRAVACSNQEIVSSK
DSPSNIEVVLSNYGAGKLSFTYNSSLANRSDFHLPDIKLICKKMEARCLRKYKSLEPNFSFKNL
IKDTCQCIVESSGPRHEGIIQTVPALDILSKPSVPQILQSNQANSAFMPPNNVMSLGGTSSSCTV
AGVSQNSSNMPVVPHQLHIGANRPPHDVNDITKGEERLRIPIINEYGNGILPPPFHYIPHNITLQ
EAYVNISLARIGDDNCCSDCFRDCLAQSLPCACAAETGGEFAYTTDGLLKGAFLDSCISMIRE
PLKHPHFYCKICPNERMKIEVNSDSSNTEMNPGPCKGHLTRKFIKECWRKCGCTRNCGNRVV
QRGITRHLQVFLTPEKKGWGLRSTEKLPRGAFVCEYVGEILTNIELYDRTIQKTGKAKHTYPL
LLDADWGTEGVLKDEEALCLDATFYGNVARFINHRCFDANIIGIPVEIETPDHHYYHLAFFTT
RIIEPFEELTWDYGIDFDDVDHPVKAFKCHCGSEFCRDKTRRSKSRARV

>OsKMT1B;3

MQPELVPEWKTWKQEVMKQFFSSHPVGNAGNFEQHNCYDDPGMDQQARIKHSKLEVRRGE
AHFSQEDDANLNTLSEDPNKSNLPSISIIHEAVGPLESRDQNKTAAFPSTSGVQDTGEPNSALH
NVRHELDSFKSSRQCSAYIEAKGRQCGRWANDGDIYCCVHQSMHFADHSSREDKSLTVETPL
CGGMTNLGRKCKHRAQHGFIFCKKHRFQTNPDAMSSDSLLSSSEGRKWEESQKSVEKMSSS
NATCSVGSEQANNFQVAVHMKVTPTMAVETTSDKVNVSENADLCYPMSTSMENSNLDASIC
IGIRSHDNIAECQDYAVRHTLYCERHIPKFLKRARNGKSRLISKDVFINLLKCCTSRKEKLCLH
QACEFLYWFLRNNLSHQRPGLGSDHMPQILAEVSKNPDVGEFLLKLISSEREKLSHVWGFGT
DSSNQMHSENQDGSVMVLREDGTHPSPGLKCKICSQEFSDDQGLGLHWTEVHKKEVRWLFR
GYSCAVCMDSFTNRRVLERHVQEKHGAQYLQYSTLLRCISCNSNFLNTDLLWQHIVSDHSRD
FSLLDHVPRRPRGQSIKRTERASDELLYDNHNLGKDDGLQKFTCRLCGMMFDLLPDLGHHH
QVAHTNSGTVSDIPSGREKYQFNRGRHYYSAFKKSLRPSGSLKKRTSSGVEKHFKAQSLDLS
MDTSHIVESETTTLGRLLDFQCSDVALTLFSKIQKTRPHPSNLDILSIARSVCCKTSLRAALKA
KYGILPDNIFVKAAKLCSDVGIQIDWHQEEFFCPKGCKSRSSSNSLLPLQPTQVDFVMSPPIGD
EIWGMDEYHYVLDSEHFGWNLKNEMVIVCEDVSFGREKVPVVCAIDVDAKEFPYMKPGEIL
QSENSLPWQGFHYVTKRLMDSSLVDSENTMVGCACSHAHCSPEECDHVSLFDSIYENLVDLH
GVPMRGRFAYDENSKVILQEGYPIYECNSSCTCDASCQNKVLQRGLLVKLEVFRTENKGWA
VRAAEPIPQGTFVCEYIGEVLKMKDDGAIRHVEREAKSGSSYLFEITSQIDRERVQTTGTTAY
VIDATRYGNVSRFINHSCSPNLSTRLVSVESKDCQLAHIGLFANQDILMGEELAYDYGQKLLP
GDGCPCHCGAKNCRGRVY

>OsKMT1B;4

MRNSATPGAVGELAELVLPWLPPQDLAAAASASRALRAAASSVSAGRAADAAHGLEPHPIPF
DNLVDGKPYAYFLYTPFSLTPSSASASPRRAQPWGRTWARPPGPTWPRSDLGGFPSSGCACA
QGACGGARGCPCADPEAEAVGLGSEAGMGSLRECGDGCACGPSCGNRRTQLGVTVRLRVV
RHREKGWGLHAAEVLRRGQFVCEYAGELLTTEEARRRQGLYDELASVGKLSPALIVIREHLP
SGKACLRVNIDATKVGNVARFINHSCDGGNLHPVLVRSSGSLLPRLCFFAARDIIEGEELTFSY
GDARLRPNGLPCFCGSLCCSGLLPSEET

KMT2

>AtKMT2;1a

MACFSNETQIEIDVHDLVEAPIRYDSIESIYSIPSSALCCVNAVGSHSLMSKKVKAQKLPMIEQF
EIEGSGVSASDDCCRSDDYKLRIQRPEIVRVYYRRRKRPLRECLLDQAVAVKTESVELDEIDC
FEEKKRRKIGNCELVKSGMESIGLRRCKENNAFSGNKQNGSSRRKGSSSKNQDKATLASRSA
KKWVRLSYDGVDPTSFIGLQCKVFWPLDALWYEGSIVGYSAERKRYTVKYRDGCDEDIVFD
REMIKFLVSREEMELLHLKFCTSNVTVDGRDYDEMVVLAATLDECQDFEPGDIVWAKLAGH
AMWPAVIVDESIIGERKGLNNKVSGGGSLLVQFFGTHDFARIKVKQAISFIKGLLSPSHLKCK
QPRFEEGMQEAKMYLKAHRLPERMSQLQKGADSVDSDMANSTEEGNSGGDLLNDGEVWL
RPTEHVDFRHIIGDLLIINLGKVVTDSQFFKDENHIWPEGYTAMRKFTSLTDHSASALYKMEV
LRDAETKTHPLFIVTADSGEQFKGPTPSACWNKIYNRIKKVQNSDSPNILGEELNGSGTDMFG
LSNPEVIKLVQDLSKSRPSSHVSMCKNSLGRHQNQPTGYRPVRVDWKDLDKCNVCHMDEEY
ENNLFLQCDKCRMMVHAKCYGELEPCDGALWLCNLCRPGAPDMPPRCCLCPVVGGAMKPT
TDGRWAHLACAIWIPETCLSDVKKMEPIDGVNKVSKDRWKLMCTICGVSYGACIQCSNNSC
RVAYHPLCARAAGLCVELENDMSVEGEEADQCIRMLSFCKRHRQTSTACLGSEDRIKSATHK
TSEYLPPPNPSGCARTEPYNCFGRRGRKEPEALAAASSKRLFVENQPYVIGGYSRLEFSTYKSI
HGSKVSQMNTPSNILSMAEKYRYMRETYRKRLAFGKSGIHGFGIFAKLPHRAGDMMIEYTGE
LVRPSIADKREQLIYNSMVGAGTYMFRIDDERVIDATRTGSIAHLINHSCVPNCYSRVITVNGD
EHIIIFAKRHIPKWEELTYDYRFFSIGERLSCSCGFPGCRGVVNDTEAEEQHAKICVPRCDLID
WTAE

>AtKMT2;1b

MISMSCVPKEEEGEDTQIKTELHDHAADNPVRYASLESVYSVSSSSSSLCCKTAAGSHKKVN
ALKLPMSDSFELQPHRRPEIVHVYCRRKRRRRRRRESFLELAILQNEGVERDDRIVKIESAELD
DEKEEENKKKKQKKRRIGNGELMKLGVDSTTLSVSATPPLRGCRIKAVCSGNKQDGSSRSKR
NTVKNQEKVVTASATAKKWVRLSYDGVDPKHFIGLQCKVFWPLDAVWYPGSIVGYNVETK
HHIVKYGDGDGEELALRREKIKFLISRDDMELLNMKFGTNDVVVDGQDYDELVILAASFEEC
QDFEPRDIIWAKLTGHAMWPAIIVDESVIVKRKGLNNKISGGRSVLVQFFGTHDFARIQVKQA
VSFLKGLLSRSPLKCKQPRFEEAMEEAKMYLKEYKLPGRMDQLQKVADTDCSERINSGEEDS
SNSGDDYTKDGEVWLRPTELGDCLHRIGDLQIINLGRIVTDSEFFKDSKHTWPEGYTATRKFI
SLKDPNASAMYKMEVLRDAESKTRPVFRVTTNSGEQFKGDTPSACWNKIYNRIKKIQIASDN
PDVLGEGLHESGTDMFGFSNPEVDKLIQGLLQSRPPSKVSQRKYSSGKYQDHPTGYRPVRVE
WKDLDKCNVCHMDEEYENNLFLQCDKCRMMVHTRCYGQLEPHNGILWLCNLCRPVALDIP
PRCCLCPVVGGAMKPTTDGRWAHLACAIWIPETCLLDVKKMEPIDGVKKVSKDRWKLLCSI
CGVSYGACIQCSNNTCRVAYHPLCARAAGLCVELADEDRLFLLSMDDDEADQCIRLLSFCKR
HRQTSNYHLETEYMIKPAHNIAEYLPPPNPSGCARTEPYNYLGRRGRKEPEALAGASSKRLFV
ENQPYIVGGYSRHEFSTYERIYGSKMSQITTPSNILSMAEKYTFMKETYRKRLAFGKSGIHGFG
IFAKLPHRAGDMVIEYTGELVRPPIADKREHLIYNSMVGAGTYMFRIDNERVIDATRTGSIAH
LINHSCEPNCYSRVISVNGDEHIIIFAKRDVAKWEELTYDYRFFSIDERLACYCGFPRCRGVVN
DTEAEERQANIHASRCELKEWTES

>AtKMT2;2

MVAVDSTFPSHGSSYSSRRKKVSALEPNYFGSMCMGVYSDDVSISAREVAQDYSCDSCGDL
ATVSSACCNFDELCGLDSALEMGCRSNEDCRAGQEASGSGIASGLDKSVPGYTMYASGWMY
GNQQGQMCGPYTQQQLYDGLSTNFLPEDLLVYPIINGYTANSVPLKYFKQFPDHVATGFAYL

QNGIIS VA PS VT SFPPS SSNA T VHQDEIQT EHA TSA THLISHQTMPPQTSSNGS VLDQLTLNHEE
SNM LASFLSLGNEHA CWFLVDGE GRNHGPHSILELFSWQQHGY VSDAA LIRDGEN KLRPITL
ASLIGVWRVKCGDA NCDEPVT GVNFISEVSEELS VHLQSGIMKIARRA LLDEIISS VISDFLKA
KKSDEHLKS YPPTSA VESISSRVINA EKS VVSNTESA GCKNTMNEGGHSSIAA ESSKYTKS VGS
IENFQTSCSA VCRTLHHHCM QIMWNA VFYDT VA THSSCW RKNKIWFRSSDIST VNYCK GSHT
KYSDKPESFESFTCRVDSSSSKTA YSDEFDLATNGA RVRGLSSDTYGTES VIASISEHVENELF
LSLKTHLTDYTSILIKDGANNTTSSA RDGKMHEGSFREQYNLE GSSKKKNGLNVVPAKLRFS
NDFSDSQRLLQEGESSEQITSEDIIANIFSTALETSDIPVNDELDA LAIHEPPPPGCESNINM PCLR
YKYQPVRSKESIPEIKA YVSMA LCRQKLHNDVM RDW KSLFLKCYLNEFLASLKGSHQVSRK
ETLA LKKRKTVTRNKKLVQSNISNQTAEKLRKPCVGASEKVLVKRSKKLSDSHSM KEVLKV
DTPSIDLS VRKPSQQKM RNTDRRDHCIIKDATKLHKEKVGKDA FSKVICDKSQDLEM EDEFD
DALLITRLRRISRNKTKELRECRNAAKSCEEIS VTA EESEET VDCKDHEESLSNKPSQKVKKA
HTSKLKRKNLSDA RDEGTKSCNGA VKSFTEISGKEGDTESLGLA ISDKVSHQNLSKRRKSKIA
LFLFPGFENTSRKCFTKLLSPEDAAKNGQDMSNPTGNPPRLA EGKKFVEKSACSISQKGRKSS
QSSILKRKHQLDEKISNVPSRRRLSLSSTDSEDA VIKEDYDVRNEEKLPCHTSDKLQKGPNKLI
RRRKPLAKHTTERSPIKDLSVDDGRPKPIALKPLEKLSSKPSKKKLFLSIPKSDGCARTSINGW
HWHAWSLKASA EERA RVRGSSCVHMQHFGSKSSLTQNVLSA RTNRAKLRNLLAAADGA DV
LKMSQLKA RKKHLRFQQSKIHDW GLVA LEPIEA EDFVIEY VGELIRSSISEIRERQYEKM GIGS
SYLFRLDDGY VLDATKRGGIA RFINHSCEPNCYTKIIS VE GKKKIFIYA KRHIDA GEEIS YNYKF
PLED DKIPCNCGA PN VYCFCEQVPW IAKLKRRTWFSRRN

>AtKMT2;3a

MILKRTLTTFENQNLKRCKIDSEIEYGRKKGEIIVYKKRQRATVDQPCSKEPELLTSSSSSLTSK
EESQQVCSDQSKSSRGRVRA VPSRFKDSIVGTWKSSRRKGESTESSHDDDDVSLGKKVKGFS
GSSKLHRSKDSKVFPRKDNGDSSEVDCDYW DVQISYDDANFGMPKKSDASRKGVYKPEE FT
VGDL VWA KCGKRFPA WPA VVIDPISQAPDGVLKHCVPGAICVM FFGYSKDGTQRDYAW VR
QGM VYPFT EFMDKFQDQTNLFNYKA SEFNKALEEA VLA ENGNFGDA EIISPDSSATESDQDY
GPASRFQA LMPLKSLKRTKDSQPEELLCKHCSKLRKSNQ YCGICKRIW HPSDDGDW VCCDG
CDVW VHA ECDNITNERFKELEHNNYYCPDC KVQHELTPTILEEQNS VFKSTEKTTETGLPDA I
TVVCNGMEGTYIRKFHAIECKCGSCGSRKQSPSEW ERHTGCRAKKWKYS VRVKDTML PLEK
WIAEFSTYTLETQMLDKQKMLSLLEEKYEPVRA KWTTE RCA VCRW VEDW EENKMIICNRCQ
VA VHQECYGVSKSQDLTSW VCRAC ETPDIERDCCLCPVKGGA LKPSDVE GLW VHVTCAWF
RPEVGFLNHENMEPA VGLFKIPANSFLK VCTICKQTHGSCVHCCKCATHFHAMCASRA G YN
MELHCLEKNGVQRTRKSVYCSFHRKPDPDS VVVVHTPSGVFGSRNLLQNQYGRA KGSRLVL
TKKM KLPGFQTQTQA EQSRVFDSLSAARCRIYSRSNTKIDLEA ISHRLKGPSHHSLSAIENLNS
FKATDFTSFRERLKHLQRTEN FRVCFGKSGIHGW GLFARKSIQEGEM IIEYR GVKVRRSVA DL
REANYRSQGKDCYLFKISEEIVIDATDSGNIA RLINHSCMPNCYA RIVSM GDGEDNRIVLIA KT
NVAA GEELTYDY LFEVDESEEI KVPCLCKAPNCRKFMN

>AtKMT2;3b

MIIKRKFKTQIPSLERCKLGNESRKKKRKLNLGGGGYYYPLNLLGEIAA GIVPGNGRN GFSAS
WCTEVTKPVEVEESLSKRRSDSGT VRDSPPA EVSRPPLVRTSRGRIQVLPSRFNDS VLDNW RK
DSKSDCDLEEEEIECRNEKVVSFRVPKATNLKSKELDRKSKYSA LCKEERFHEQHNDEARA R
VDEKLPNKKGTFGPENFYS GDLVWAKSGRNEPFWPAIVIDPMTQAPELVLRSCIPDAACVVF
FGHSGNENERDYAW VRRGMIFP FVDYVA RFQEQPELQGCKPGNFQMA LEEAFLA DQGFTEK
LM HDIHLAA GNSTFDDSFYRW IQETA VSNQELNNNAPRQGLLKKHRNPLA CA GCETVISFEM

AKKMKDLIPGDQLLCKPCSRLTKSKHICGICKKIRNHLDNKSWVRCDGCKVRIHAECDQISD
RHLKDLRETDYYCPTCRAKFNFDLSDSEKQNSKSKVAKGDGQMVLPDKVIVVCAGVEGVYF
PRLHLVVCKCGSCGPKKKALSEWERHTGSKSKNWKTSVKVKSSKLALEDWMMNLAELHA
NATAAKVPKRPSIKQRKQRLLAFLSETYEPVNAKWTTERCAVCRWVEDWDYNKIIICNRCQI
AVHQECYGARHVRDFTSWVCKACERPDIKRECCLCPVKGGALKPTDVETLWVHVTCAWFQ
PEVCFASEEKMEPAVGILSIPSTNFVKICVICKQIHGSCTQCCKCSTYYHAMCASRAGYRMEL
HCLEKNGQQITKMVSYCAYHRAPNPDNVLIIQTPSGAFSAKSLVQNKKKGGSRLISLIREDDE
APAENTITCDPFSAARCRVFKRKINSKKRIEEEAIPHHTRGPRHHASAAIQTLNTFRHVPEEPKS
FSSFRERLHHLQRTEMDRVCFGRSGIHGWGLFARRNIQEGEMVLEYRGEQVRGSIADLREAR
YRRVGKDCYLFKISEEVVVDATDKGNIARLINHSCTPNCYARIMSVGDEESRIVLIAKANVAV
GEELTYDYLFDPDEAEELKVPCLCKAPNCRKFMN

>AtKMT2;3c

MIIKRKLKTLKRCNSTNEEDDIVRKKRKVNLNGGGSGGDYYYPLNLLGEIGAGIVPGKNGFS
VSLCKQVSCSPKVEVVEEEEEEEEIKSTRLVSRPPLVKTSRGRVQVLPSRFNDSVIENWRKDN
KSSGEEREEEIEEEACRKEKVKVSSNHSLKIKQQETKFTPRNYKYSSSSALCGEIDDEDKCEEI
VRYGNSFEMKKQRYVDDEPRPKKEGVYGPEDFYSGDLVWGKSGRNEPFWPAIVIDPMTQAP
ELVLRSCIPDAACVMFFGHSGTENERDYAWVRRGMIFPFVDYVERLQEQSELRGCNPRDFQ
MALEEALLADQGFTEKLMQDIHMAAGNQTFDDSVYRWVEEAAGSSQYLDHVAPSQDMKK
YRNPRACVGCGMVLSFKMAQKMKALIPGDQLLCQPCSKLTKPKHVCGICKRIWNHLDSQSW
VRCDGCKVWIHSACDQISHKHFKDLGETDYYCPTCRTKFDFELSDSEKPDSKSKLGKNNAPM
VLPDKVIVVCSGVEGIYFPSLHLVVCKCGSCGPERKALSEWERHTGSKAKNWRTSVKVKSSK
LPLEEWMMKLAEFHANATAAKPPKRPSIKQRKQRLLSFLREKYEPVNVKWTTERCAVCRWV
EDWDYNKIIICNRCQIAVHQECYGTRNVRDFTSWVCKACETPEIKRECCLCPVKGGALKPTD
VETLWVHVTCAWFQPEVCFASEEKMEPALGILSIPSSNFVKICVICKQIHGSCTQCCKCSTYY
HAMCASRAGYRMELHCLEKNGRQITKMVSYCSYHRAPNPDTVLIIQTPSGVFSAKSLVQNKK
KSGTRLILANREEIEESAAEDTIPIDPFSSARCRLYKRTVNSKKRTKEEGIPHYTGGLRHHPSAA
IQTLNAFRHVAEEPKSFSSFRERLHHLQRTEMERVCFGRSGIHGWGLFARRNIQEGEMVLEYR
GEQVRGIIADLREARYRREGKDCYLFKISEEVVVDATEKGNIARLINHSCMPNCYARIMSVGD
DESRIVLIAKTTVASCEELTYDYLFDPDEPDEFKVPCLCKSPNCRKFMN

>GrKMT2;1

MAFPEKGDEEDADTPIRYVSLDRVYSSASVCVSATNSSNVMSKKVKARKLIVDNDHPLKTH
NPPVVHVYSRRLKRPRQCVSFYDSLLEGESQKTAVKSEIDESLRKKRRIGSNELANLGVDSSV
LCQSDRPRLRDCRNNCSVNNNVNSNSVKKRKHNSTLNSQRSFTASATAKKWVRLSFDGVDP
KAFIGLQCKVFWPLDADWYLGRVVGYNLETNRHHVEYVDGDEEDLILSNERLKFHVSHEEM
ERLNLSFSVDSTKDDDHDYDEMVALAASRDDCQELEPGDIIWAKLTAFSAGHAMWPAIVVD
ESLLGDRKGLSKISGGRSVPVQFFGTHDFARIKLKQVVSFLKGLLSSFHRKCKKPRFSRGLEEA
RLFLCEQKLPRRMLQLQNGIVVDGGEASSEDEGGKDSIDDHLKKDQGIQITLGGHGGSSYVIG
GLQIINLGKVVKDSEYFQDDGIIWPEGYTAVRKFTSVKDPSVCTLYRMEVLRDPQSKNHPLFR
VTSDEEKFEGPDPSACWNMIYERIRKRQNDSSDCKAGKGRLFEPGADMFGFSNPEVTKLIQG
LSKSRLSSKFSAFKLASGRFRDLPAGYRPVRVDWKDLDKCSVCHMDEEYENNLFLQCDKCR
MMVHARCYGELEPLDGILWLCNLCRPGAPESPPSCCLCPVIGGAMKPTTDGRWAHLACAIWI
PETCLSDVKRMEPIDGLNRISKDRWKLLCSICGVSYGACIQCSNPTCRVAYHPLCARAAGLCV
ELEDEDRLFLLSVDEDDEDQCIRLLSFCKKHRQPSNDRVASDERFGRIARRCSDYTPPLNPSGC
ARTEPYSHFGRRGRKEPEALAAASLKRLFVENQPYLVGGCCQHGMSGSTVPNNRVSGIKFSF

SLNKLKAPQLDAPNNILSVAEKYDYMKQTFRRRLAFGKSGIHGFGIFAKHPHRAGDMVIEYT
GELVRPSIADRREHFIYNSLVGAGTYLFRIDNERVIDATRAGSIAHLINHSCEPNCYSRVISVHG
NEHIIIFAKRDIKRWEELTYDYRFFSIDERLACYCGFPRCRGVVNDTEAEEQVSKILVNRGELI
QWTGE

>GrKMT2;2a

MGLPMVSSTSPIDEYDHPNFSRKRLKVSDLSADFASSNRGEEQFATEMSCQSNGNSSGISQSC
NGGGSSCDKSYSSYAPSSSSYVSGWMYVNDHGQMCGPYIQQQLYEGLSTGFLPDELPVYPV
VNGALINPVPLKYFRQFPDHVATGFLYLTSPTASNYLKSSFTNVQHTPSPSPSQFNCNSGEDEC
WLFEDDESRKHGPHSLLQLYSWHRCGYLADSIMIYHTDDRFRPTQLLSVLNAWKGGQYVAE
NEQELSVNFISAISEEVSSDLHSRIMKAARRVMLDEIISTMISEFVAAIKSQRPLMVESYNQDA
KSSDGKLIKNTMERSIHCTSKFGTATSDGVSNFSIQESTHFDASLKSVGSLENFWGSYTVVCK
MLFEYCMQVTWNAVFYDSMVEYLSSWRKGKLWYGQPNVLASASGSIGHGKETENIKATTL
FSRMELTAYDIESPPGYELETVSVGNQAEETYISQSAVQEILSKQNSSLHNSGLYGGIQCILEG
VGNELHSSVMVFMADYLDGLVKSEAKIVIDLENDKKLNETPDEEAAEKSVCLSVDDELKEL
QDTVGSSDQCHLASEVDNSDDSEEKKNVSNRMSDLSCNLQNSVQSKKPVCQSISENNYASRQ
ETFMAEAFKRLFGKVGDVSNEQEVNEPPPPGLEVKSGTLVPSHNCKFRPLTSVGCSPKIGEYV
AMAMCRQKLHDDVLREWKSSFAGDASLYQFLILRSSSKKHCKADGKEAKTFSEDRKNLAGF
SASRDKPRDGSRKSLSSGSSDISLVTGTCTYYRKKKLVHKKVGSSLSTIINGSRDQPVERPRTK
RPSKNLLDHADQKLSAATSKKGGTNKSMSQSSNISRSSKIIAKNSLPNDHSLPKSAIGRKTSKG
AAAAVRKNLIGEGAIKVGRERASTFQNCDVEKIARKSNHTVGSEGEVTNDSSKKTLKAKKVS
GVKRKQLNYDECPSPSIKVQKVASCGSKSSSSRGVADQKSRTVRSRTANPCPRSDGCARTSIN
GWEWHKWSLNASPAERARVRGVQCIQMKYSGPEVNSMTHLSNSKGLSARTNRVKLRNLLA
AVEGADLLKATQLKARKKRLRFQRSKIHDWGLVALEPIEAEDFVIEYVGELIRPRISDIREHYY
EKMGIGSSYLFRLDDGYVVDATKRGGIARFINHSCEPNCYTKVISVEGQKKIFIYAKRHIAAG
EEVTYNYKFPLEEKKIPCNCGSKKCRGSLN

>GrKMT2;2b

MLFTWKVEGPLHDGGNSTLRSLGVPEDQSGWDWPEGLLPTTGLVKSSSTVPKISITSHVANPI
GSSQALSSLLDVADGRRMECAFSKSSTMSTFGGRDSSNGCQSVSTWIDSILKTRNQSLAHSSL
QNLRTLGRNYDATAAKITGDIVISDRDAASSNVELKLGQPYQPSQPIGNSALPVVAPKHFKTV
VDPPQSYHSEKMVDRAMFFGKEETRQYCLQEADSSNTMARSQQSLLNFSKHAFAASSFVDA
TKPESRLDATKNLVVPAHSLLPLEGSACSKGTNNMVSEFSMPKIFHCGSSTTKCDPLNAPLKI
GNSLGRQLNMPELGFCRLTEKGKGAGLGCLPVGFSDAIDPALRIHKQEESSRIAIGMMPGFSS
VHNMNSCQSSNVSSGRFDERSCLNLPGNSSFVGNNAHTDQAFLTMSSSYLGSRHISQPSAATT
GFLLATSTFVPGSTSTTSKQEGPCLLDDSMRVLALSQISELSKRHAISSVGMSHELGRLDRTSN
PNIQHSLLESSKSREGRCGSILPSKQDVFEGASSFPSAAEKSIPMTGLSSCCDFSQLTQGLPLH
SRIDMPCQFSNDRFCDHSTLRLIRGESITQSSGHAKCCQRVPCTYSRGDCICSVHAKCLEGYTK
CRFEGSCVVSKEQVGVCCEAHNPVASEFVKEHIYPNDRTNLLDQGGKVNGQLPMRIACHAS
QWRDVPSKQKEACKMTQINLSAELLDASGCAGDQFGASAGVHGIGSAINEADSLKWQGMS
NISSGCSGAAVTQASTEVNNVDSSTIDAGDNEYRNDLVVDEGSGIDKCSSNDELESERSAEFI
GVSCRNKIRNNRSSKIPNGQSSLSLLDELKLLDSLTWKKSQNQSYMSLSSIERINHVKKIRKGL
KGGKRKRATKFRMLDMSFAPKLSLQHCPKGNGSHHMSSRSSKDWQTLIPSVLESYEPTHLIR
PGELASAKIVCRKRDLSDVYRDQDGEDYQVKLKDDDRSDNILEVSGRKRLKQNLAYNSFEK
LGSPKPLRTVEKTSNSDSVYCTNAFSCFETVCDKKVRPIVCGEYGEICSCKSAAAEFKPPKIVP
LSRVIKSLDQRNLRKSCKPKITSRMSNKKRTATTTGYFDLSSDLKKEEENGAHHVSFFDEVSG

CLVEEGKKTCLGEIKQFHNMSFILEEGNADGSEKSCVPDAITCNWSNARCKESRKRSLSELTG
KGKESRSDSYPLVEISKCMPRMKARKGLNKADDVECHGHRACDINPEKAIDELRCSSSADSD
AFCCVCGSSNKDEFNCLLECSQCSIRVHQACYGVSKVPKGQWYCRPCRTNSKDIVCVLCGY
GGGAMTRALQSHTFVKGLLKAWTIESECRPKDTVSSAETMVDDQSLVVGKELCNLQCKDLG
LSRTAVWKMDMQNSLNNIQNSPCSVSKLNVYNSVIAGVLDPSVKQWVHMVCGLWTPGTRC
PNVNTMSAFDVSGVSRGRENVVCSICNRSGGSCIQCRVVDCSVRFHPWCAHQKGLLQSEVE
GLDNESVGFYGRCMLHALHPICESDSDPTNGKLSFPRKGESTCARTEGFKGCKQDGFWHNPY
GQSRRKNGCFVPQEQLNAWIHINGQKPYMQRLPKLSKSDIENDCRKEYARYKQAKGWKHL
VVYKSGIHALGLYTSRFISRGEMVVEYVGEIVGQHVADKRELEYLSGRKVQYKSACYFFRID
KEHIIDATRKGGIARFVNHSCLPNCVAKVISVKNEKKVVFFAERDIYPGEEITYDYHFNHEDE
GKKIPCFCNSKNCKRFLN

>GrKMT2;3a

MIIKRTGNLEMRSMKRCKMEREGYDDCGEEEIFYDYEGNPKRLKSNGYYSYGEFDDFSSGSG
YWSSEESYWAGEFESNSMNLNKAKQNKKSSKRNFKPPLLKSSRGRTQTLPSRFNDAVLDSW
KSRKLRVDYADSSLEDDEFDGSRYLKDKYGYGSSDLYLISKRREERGMSCVGINNSYEYGSY
LDSSSSTLLETEETVPYNSFKGIERLKKERGGKRKDVYKPEDFALGDLVWAKCGKRYPTWPA
IVIDPILQAPESVLSCCVPGAICVMFFGYSKNGTQRDYAWVKQGMIFPFAEFMDRYQGQTQLF
KWKQSDFQMALEEAVLAENGFLDSEHKTQQIGYADAQPSCSSQDLDCLYTHNQDTRPCDSC
GSVVPLKTMKKMKKSAELLCKHCYKLRKSKQYCGICKKIWHHSDGGNWVCCDGCNVWVH
AECDNISSKVFKDMENIDYYCPECKAKSKLESCLAKREPKIKSREKSGESVPPDKLTVVCNGM
EGSYIPNLHLVVCECGACGSKKYTLSEWERHTGCRAKKWKYSVKVKDTMTTLEKWIVDYN
AHGVHTLKLDKQKLIGFLQEKYEPVDAKWTTERCAVCRWVEDWDYNKIIICNRCQIAVHQE
CYGASNVQDLTSWVCRACETPDIERECCLCPVKGGALKPTDVESLWVHVTCAWFRPEVGFL
DHEKMEPAVGVLRIPPTSFLKSCVICKQTHGSCTQCCKCSTYFHVMCASRAGYIMEIHCSEKN
GMQMTKKFVYCTDHRSPNPDAVVVMHTPSGVFAARNVLQNVNDCPRGSRLISSKNAELPES
PALDTNDFDACSAARCRIFTRSKFKGAEREPIFHSPSGPSHHHIDALRALSTYKEVDDSTVFSS
FKDRLYQLQRTENHRVCFGKSGIHGWGLFARRNIQEGEMVVEYRGEQVRRSVADLREAQYR
SEGKDCYLFKISEEVVIDATNKGNIARLINHSCTPNCYARIMSVGDEESQIVLIAKTDVSAGDE
LTYDYLFDPDEHDELKVPCLCKAPNCRKYMN

>GrKMT2;3b

MIIKRNLKSQMPSLKRCKLGSSVGEYEDNSGRARKKRKINGYYPLALLGEVAAGTIPFKLHHI
ITSAEADKGFSASWCTEVSCSPDEVESKSNGLDSSKAKNRTVEITRPPLVRTSRGRVQVLPSRF
NDSVIENWKKEGKTSLPDDSLDDGDDDDDYDFECKNDKFSFKKPKVCKQNMKNRRAEEKN
GYKGRKYTTLCEEDGREAEYARTFDIRKYSSSRSTLTKVHERFVKEEKYVNGVDTFDLTTGK
QASLENGERKDGLYGPQDFYSGDIVWARPGKREPFWPAIVIDPMTQAPELVLRSCIPEAACV
MFFGHSGTESEGDYGWVRRGMIFPFIDFLDRFHDQPELRRCKPSDFQLAVEEAFLAEQGFTEK
LIHDIDMAAGNTNYEESVFRWVQGATGPNRDHDYRLSNQGFFGKHTDSRLCEGCGMNLPFK
MGKKMKASAPAGQFLCKTCARLTKSKHYCGICKKVWNHSDSGSWVRCDGCKVWVHAECG
KISSHHFKVLGATDYYCPPCKVKFNFELSDSEKWQPKTKSKTNNSQLVLPNKVTVLCCGVEG
IYYPSLHSVVCKCGSCGLEKQALSEWERHTGSQQRNWRISVKVKGSLLPLEQWMLQLAEYH
ANAATSTKPPKRPPIRERKQKLLAFLKEKYEAVHVKWTTERCAVCRWVEDWDYNKIIICNRC
QIAVHQECYGARNIRDFTSWVCKACETPEVARECCLCPVKGGALKPTDVETLWVHVTCAWF
RPEVSFASDEKMEPALGILSIPSNSFVKICVICKQIHGSCTQCCKCSTYYHAMCASRAGYRMEL
HCLEKNGRQITKMVSYCAYHRAPNPDTVLVIQTPLGVFSAKSLVQKKKKNGSRLISSNRLKIE

EVPTEETIEIEPFSAARCRMFKRSNNNRKRTEEEAVAHRLMRPYHHPLRTIQSLNAFRTVEEPK
GFSSFKERLHHLQRTEHDRVCFGRSGIHGWGLFARRNIQEGEMVLEYRGEQVRRSIADLREA
HYRKEGKDCYLFKISEEVVVDATDKGNIARLINHSCMPNCYARIMSVADDESRIVLIAKTNVS
AGDELTYDYLFDPDEPEEFKVPCLCKAPNCRQFMN

>GrKMT2;3c

MIIKRNLKSQMPSLKRCKLGDSVGEDEDSSGTTRKKRKINYYYPLTLLGHVAAGIIPVSLHYII
ASGELEKGFAASWNTEVSCSPDEVESKSKRSDSSKPKNRTVEIARPPLVRTSRGRVQVLPSRF
NDSVIESWKKESKTSLHDHSFDEDEDDDDFECKKDKFSFKSSKNCKQNMKNQRNKENNGYK
GRKYTTLCEEDDREAGYGRSSGIGKYSSFRSSLTSVHEQLVGEYEKNANGVLIVDLTTQGQA
SRENGEKEDGLYGPEDFYSGDIVWARPGKREPCWPAIVIDPMTQAPELVLRSCIAEAACVMF
FGHSGNENQRDYAWVRRGMIFPFADFLDRFHEQPELAGCKPSDFQLAMEEALLAEEGFTEKL
MDDINIAAGNPAYDESILRWGQEATGSNQDLGYCLPNQGLFGKCKDARFCESCGMTLPFKM
GKKMKASTPGGQFLCKTCARLTKSKHYCGICKKIWNQSNSGSWVRCDGCKVWVHAECDKI
SSRHFKDLGATDYYCPACKAKFNFELSNPEKWQPKAKSKNNNSQLLLPNKVTIVCCGVEGIY
YPSLHLVVCKCGSCGSEKQALSEWERHTGSRERNWRVSVKVKGSLLPLEQWMLQLAEYHA
NATSSAKPPKRPSLRERKQKLLAFLQEKYEPVRAKWTTERCAVCRWVEDCDYNKIIICNRCQI
AVHQECYGARNVRDFTSWVCKACETPEVTRECCLCPVKGGALKPTDIETLWVHVTCAWFQP
EVSFASDEKMEPALGILSIPSDSFVKICVICKQIHGSCTKCCKCSTYYHAMCASRAGYCMELH
CLEKNGRQITKKVSYCAYHRAPNPDTVLIIQTPLGVFSAKKKSGSRLISSKRVKIEEVPTVETT
DVEPFSAARCRVFKRSNNNRKRTEEEAVAHRLMRPCHHPLSTIQSLNAFRMVEEPTGFSSFRD
RLYHLQRTENDRVCFGRSGIHGWGLFARRNIQEGEMVLEYRGEQVRRSIADLREARYRIEGK
DCYLFKISEEVVVDATDKGNIARLINHSCMPNCYARIMSVGDDESRIVLIAKTNVSAGDELTY
DYLFDPDEPEEFKVPCLCKAPNCRKFMN

>OsKMT2;1

MVIAVEGGFVHEEEEVDHPIRYLPLGRVYSSSAPCPLPKKPRSAEDGKPPVIVYYRRRRKKPR
VEGPPPSPATAPPMLHPREDDEDEEVTRRKGSLKYELLSLGQAPPALGGDGEEPARRRCLRRS
GGAERRGYFSEPKRRQRQGVHKEAASSAGRRWLELEIEAADPLAFVGLGCKVFWPLDEDWY
KGSITGYNEATKKHSVKYDDGESEDLNLADERIKFSISSEEMKCRNLKFGISNLNKRGYDELL
ALAVSLHDYQGLDPGDLVWAKLTGHAMWPAVVVDESNVPANRALKPGRLDQSILVQFFGT
HDFARIKLKQAVPFLNGLLSSLHLKCKQARFYRSLEEAKEFLCTQLLPENMLQLQKSMEKGS
SDANSNKDVHSCDNLSEDKTAESGGDYDEMTPIELGNLRVSKLGRIVTDSDYFHNKKHIWPE
GYTAFRKFRSVKDPHVVILYKMEVLRNSDIKARPLFRVTSEDGTQIDGSTPNTCWKEIYCRLK
EKQRNVASGLDRDVCQGSGSYMFGFSNPQIRQLIQELPNARSCLKYFENAGDTFRGYRAVHV
NWKDLDYCSVCDMDEEYEDNLFLQCDKCRMMVHARCYGELEPLNGVLWLCNLCRPEAPR
VSPRCCLCPVTGGAMKPTTDGRWAHLACAIWIPETCLKDVKRMEPIDGLSRINKDRWKLLCS
ICGVAYGACIQCSHPTCRVAYHPLCARAADLCVELEDDDKIHLMLLDEDEDPCIRLLSYCKK
HRQPSTERPSLESNLAKPAVVVQTDAVPPSGCARTEPYNIHGRRGQKQPQVMATASVKRLYV
ENMPYIVSGFCQNRVGHDAISEPIQSVGFLDVAHQEAVGNVSSMIEKYKSMKATFRRRLAFG
KSRIHGFGVFAKVSHKAGDMMIEYIGELVRPPISDIRERRIYNSLVGAGTYMFRIDDERVIDAT
RAGSIAHLINHSCEPNCYSRVISVLGDEHIIIFAKRDINPWEELTYDYRFVSSDQRLPCYCGFPK
CRGVVNDVEAEGQSAKIRVNRSELFQQ

>OsKMT2;2a

MPHSRSDSGSGSRGADPCRGRKRGRLLMLEEEEEEEESGMEGCSAPACGDVRGDFVGWCSD
RHQVASCSGDQTQSASMFAAMQENACSIDSKGVVCPQSGLGYSAGQNGTHGGGGSMLHQN

LEGCMYMNQLGQMCGPYPPEQLYDGLSTGFLHRDLAIYAVFGGKMANPVSLGSLKQFLSQ
WSSDSVVATRDESVENKKMAPVNKLILPDNLSSEESCWMFEDAEGRRHGPHSLAELSYWHH
SSYLHDLSMIYHVDSKFGPFTLVSLIDWWSGGTEHSESSANDSGSLNALMDDVVEDISHQLH
AGIMKSARKVFIDEIFSSVLPEMIACRKTEKQMAAKRKSQAAKTDNVSNKNALVLKGKGDG
TSTRPKSLNSYNNKVPEDPSVAVQSTAMQYEFADILSAVWETIYNKSMKSIWDEVLYDPVM
DYCDAWLKRKNESNLLSTVVPGASDNQKMQDTDEMSPKAICDSDAPESDMDFPPGFGPNQE
SAEHSHSACVEYVTEKTDGRSGSSITLFSGPLGRVQVMLANELYVAAKEALFQHFEEVISEEIT
NCLCIGFEDDINQERIRTPVHAPEPSSPPGISVHETPSPAEMPRDEISDMAEMARDEISDMAVD
TIPCPADMAASGTSTVPEVTTDKLIIPYVEHQSPSASHASIFEKLDAHEEAELDDSFDEVPPGTE
AGLASLVIMEKNKYQPSKSVDSVLDIYRYTSWAFFRQILHESVMKEWASLFSGALSNCFDSW
YARKNIVAKTMDDTLRPKEYTYYRKRKLRKNCEASSSEKPMDEQLSRPLRDLVECKVNMKN
IHRSSKAGISQRVSVVEKPSKKRAKPSHNDNINLNIQQDLKLLSDKVPKRNRSSHPTSKPLVSS
KVPTEDRTTSAMPAKKRKQKNLATESNLKTKAVILSPESHGCEAPTEKRTTAIMPVNKRKKN
LSGESKLKAKPLTSPESYVCEAPIDNRTTSTMPAKKRKQKNLSNESNLKKKPLVLCPESYGCA
RASVSGWEWRDWARNATPSERAQVRGYRVRSILSAPENNVLKSSQVKGSSARTNRVKLRNL
LAAAEGTDLLKIMQSKSRKKRLRFQRSKIHEWGLVALESIDAEDFVIEYVGELIRRQVSDIRED
QYEKSGIGSSYLFRLDDDYVVDATKRGGLARFINHSCDPNCYTKVITVEGQKKIVIYAKRRIY
AGEELTYNYKFPLEEKKIPC

>OsKMT2;2b

MASFYCVRVQIDSASLRHILRPFAAQGAAPPEQLAASHPSSQYGQPARAAPSTSLLAQVAGN
HPHATHVSDRKALFGMLNAGNAANVIDLTRASPLGGAEPLPKHPRHGLEASSSVEQPSCLGP
LFQNTSANVQGSFPGECSVNNGISQGAIQFQDSSTCAVQKLPSQSTPRHHPALLGDQICVSCL
NVGGEFFVGEAGIFGVRCFCHRLRMSVAKFCEHSGGPAEKAGEIVIMDNGMTIVQWLKYCM
GVGASISDTKWDWPEWAYMRYSSEEYWTKSLLTTNNNMEKTGLFSGHGKSTGHINNPVYSS
DIHNEVGRFTSVEKLVNKPDETFYRKSVGLHEAFSKNPAIQQSSKINLANHMIHDMNMNSISR
PSERTYSTANMGITYSRNHLAHDYANFLEKNLNNLSRSPGPSSTRVLSNDSRACMPDVPHKII
QDGSESLGADAYPKQTTKANKTIENIEPSFGTGNRKRSLDVSNGTSHSELNEITDDAAKNSFIS
LFLSHLERNSTSESIDDVLNSNEHYLLKAPDVAYSSDRLKTASTQVETRANDNQLKLAPAIIHT
KRISDSRSLPVPVASKGYVHQDVLHANSQEPLINGDCLPHLLPSQPNAGISKICAEVSSPVNCR
CCNHVADKSHLAHSETGAPCFYDRTENQCIDAVAKLKKILPNLVLELAISADLISVMMALQF
QPIGLDEVRTRSTFIPRSSLCSRELMLQSCCHACPIDGYYRSSMGHTANSLTKNTLLDAPNNTE
CSPYRDGKCCCSLAPKCLAGYGFTKHCVARIDQTDHTVQKSKDDGMQAAARCCTLGESEKL
ICQCSSEIIARKSDSKASFQNEVSTEVLNRPCVPTLQQLKNVTEASAVGGHWPYETVKEKASA
CRDSGIFKELKSGFSSGFSSDVVTKFSASPELNKYGLEHKNLVFDEGSRIEKCSSSSYLPISTGC
EEAQNSFSRFHLEPSLVKHKNNQISEGSTRKEHENEGQCSEMSKKTRTLRCCANKSESDDCTR
KIDLSSREGDSQPQHKAGPFSRRVSKTKRKHPPTHLNKHVKRLHSNCKVLNVDNERSDDEGI
YVGESNSSDRKKQEDNMTTLDRTKCQQQGSRLLVRKLPKYVSLNCIVNETNSEDACSGSASI
DSSLIATGITNDNRKSPKIVPLNLILKKAKRCHAIKPLSKTENIHFSEEKSSDGSADKSSSGDRSF
SPQDELWSPKKNRYSSNVSRPHVKTDCQSPCCVLEEDEPLSLADMGTSQLSASRSRGSKNQR
ACISLNRMERCEEFTNESACSPCGDKHAAVQACVTKFERYIQRPSLDASCCVCGISNLEPSNQ
LIECSKCFIKVHQACYGVLKVPRGQWFCKPCKINTQDTVCVLCGYGGGAMTRALKAQNILK
SLLRGIATAKRSDKYVYSSGNVNSECTSKLHGEYVRHDSFNGHRSRSFNAISSFGIKEASIGSA
RGDIISKSWTSNRNSSLLGPRTRQWVHVVCGLWTPGTKCPNTITMSAFDISGASPAKRNTECS
MCNRTGGSFMGCRDVNCSVLFHPWCAHQRGLLQSEPEGEHNENVGFYGRCLDHAMLDPNH

VNPKKECLRSNDWTCARTEVFRGRKGDSFGANRSRKPEEKFGECSVSQEQINAWIRINGSKS
CMRGQKEYVHYKQLKGWKHLVVYKSSIHGLGLYTSEFIPRGSMVVQYVGEIVGQCVADKR
EIEYQSGKRQQYKSACYFFKIGKEHIIDATRKGGIARFINHSCQPNCVAKIISVRNEKKVVFFA
ERHINPGEEITYDYHFNREDEGQRIPCFCRSRGCRRYLN

>OsKMT2;3a

MLASRIPLKRCTAAAVVPLPGELEMEEGPTPPAGGGEGSGAAVVPAKRRRERVVPSRFRDSV
VSLPLPPAKKGRPAKKAAAREGGDGEVYDVEVRAVEQQGATAAAFGAVETAVWPGDERPA
QTEEELYRACRNIRRSSSSSGFSGSVVTSLSNAGGSVAPEGKPVVVVECKPKREGGDKKEDFY
WPEDFVLGDVVWARSGKKCPAWPAVVIDPLLHAPAVVLNSCIPGALCVMFFGFSSGGHGRD
YGWIKQGMIFPFVDYLDRFQGQALYKLKANRFRQAIEEAFLAERGFCELEMDEGCSLEKSVN
DQSVPDGLQEGSGSNNDQECQSEAQVVGKSTGCCDSCGNRVPPKIAKKKKQAGEQLLCRHC
DKLLQSKQYCGICKKIWHHTDGGNWVCCDECQIWVHVECDLTCINMEDLENADYFCPDCKS
KRKTVPPVEQMNTPNSSECASTSKEKLPEMIPVFCFGMDGMYLPKKHMILCQCNSCKERLMS
LSEWERHTGSRKKNWKMSVKLKSNGDPLVTLLDDIPCANVKSSTPSINKEELLKLLANSFRP
VNARWTTERCAVCRWVEDWDYNKIIICNRCQIAVHQECYGARDVQDFTNWVCRACELPKQ
KRECCLCPVKGGALKPTDIDQLWVHVTCAWFQPKVSFPVDETMEPAMGILSIPSEYFKKACV
ICKQMHGACTQCYKCSTYYHAMCASRAGYRMELQYSEKNGRNITRMVSYCAFHSTPDPDN
VLIVKTPEGVFSTKFLPQNNEKQSGTRLVRKENLQEKVLPAKISDCPAARCLPYEMLKNKKEP
GEAIAHRIMGPRHHSQESIEGLNACMDQKDEKSFATFRERLRYLQKIENKRVSCGRSGIHGW
GLFAAKKIQEGQMVIEYRGDQVRRSVADLREARYHREKKDCYLFKISEDVVVDATEKGNIA
RLINHSCMPNCYARIMSVGDEKSQIILIAKRDVSAGEELTYDYLFDPDESEDCRVPCLCKALN
CRGYMN

>OsKMT2;3b

MIFKRNQRSEILSLRRCNAGGGAGVGEDDGDGGERRPKRRRGDEFFPVELLGDVPVAGIPYA
AFGFRWCEEAEVASPAAASRAAAAAAAASRPPVVRTSRGRAQVLPSRFNDSVLIDPWKKDK
PAKPPVPAKAVQLVPKSEVLYRKGAIADRSLTMSELDENGDDDYEEGHNFVASRKYSMSLST
VTSVHGEPYSYYHRKGLMKRQYDDDDDDDDDDDDDDDDEDEEAEEEEEEEEEEEEELSYW
RNDFVYGDIVWARLGKRQPVWPGVVVDPAQPAAAQALPPQPRSGAVLCVMLFGWAAEFG
DEKKFIWVRQGGIFPFVDYMDRFQGQTELSSCKPGDFQRALEEAFLADQGFFEVPMDGNTTG
QPAVCQSFPADLEEVTGSNELECQSQIKRYKRALQCESCGNCFPNKDPSMMVYVMEQLACR
QCAKILRSKEYCGVCLKSWQHKCGGRWVCCHGCESWVHAECDKKCSNLKDLRDNSYFCPY
CRVKQNSNLSSKKTKSYEHRTDNSTQKSSKPDKVAVICFGMEGTYLRDLELISCHCGPCKGQ
KFLFNEWERHAGCRSKNWKSSIKIKDTLMPFGKWIEQHQSSSYSTNPAKRSSQKMKKQKLLD
LLSEPYDTVNVKWTTERCAVCRWVEDWDYNKIVICNRCQIAVHQECYGVRGKQDFTSWVC
RACETPDQKRECCLCPVKGGALKPTNVDNLWVHVTCAWFQPQVAFASDELMEPAIGILNITP
LLFMKMCVICRQIHGSCTQCYRCSTYYHAICASRAGYRMEIRCLEKNGKQTTNKISYCAHHR
APNPDNVLIIQTPAGTISSKKLVQSNGTVAASRLIRKDLPKDSVSEVEISENLSAARCRFYVKK
ELKRSREGAIAHRVRGSCQHRWDEIDSLNPPREERDPESFSTFKERLHYLQKTEHTRVCFGRS
GIHRWGLFARRGIQEGEMVLEYRGEQVRRSVADLREEQYRVQGKDCYLFKISEEVVVDATD
KGNVARLINHSCTPNCYARIMSVGHDESRIVLIAKKNVSAGEELTYDYLFDPDEADDRKVPC
LCQTANCRKFMN

KMT3
**>AtKMT3;1**<br>MDCKENGVGDASGCNIDANSLASNLAMNTNEDFYEKLSSRGQNLDSVSSLEIPQTASSVNHT<br>IEGQRKCFTEIEQMGYGNSNSQEDAGNTDDDLYVCYNADDTQEQGVVSGELEQSQELICDTD<br>LLVNCNKLDDGKESQDTNVSLVSIFSGSMQEKEAPQAKEDEGYGGTTLPIGGSGIDTESTFVN<br>DAPEQFESLETTKHIKPDEVESDGISYRFDDGGKEGRNGPSSDLDTGSSDDISLSQSFSFPDSLL<br>DSSVFGCSATESYLEDAIDIEGNGTIVVSPSLAITEMLNNDDGGLCSHDLNKITVTETINPDLKL<br>VREDRLDTDLSVMNEKMLKNHVGDSSSESAVAALSMNNGMAADLRAENFSQSSPIDEKTLD<br>MEANSPITDSSLIWNFPLNFGSGGIEVCNPENAVEPLRIVDDNGRIGGEVASASGSDFCEAGMS<br>SSRRKARDGKQCKVVQTKTSARHLRKSSRKKQSERDIESIFKCSKQKRSSLLKTSRSSEWGLP<br>SKTTEIFLQSNNIPYDGPPHHEPQRSQGNLNNGEHNRSSHNGNVEGSNRNIQASSGSCLRLKV<br>KFGKSGGQNPLNITVSKVSGNSLPGNGIVKAGTCLELPGSAHFGEDKMQTVETKEDLVEKSN<br>PVEKVSYLQSSDSMRDKKYNQDAGGLCRKVGGDVLDDDPHLSSIRMVEECERATGTQSLDA<br>ETSPDSEVINSVPDSIVNIEHKEGLHHGFFSTPEDVVKKNRVLEKEDELRASKSPSENGSHLIPN<br>AKKAKHPKSKSNGTKKGKSKFSESAKDGRKNESHEGVEQRKSLNTSMGRDDSDYPEVGRIE<br>SHKTTGALLDADIGKTSATYGTISSDVTHGEMVVDVTIEDSYSTESAWVRCDDCFKWRRIPA<br>SVVGSIDESSRWICMNNSDKRFADCSKSQEMSNEEINEELGIGQDEADAYDCDAAKRGKEKE<br>QKSKRLTGKQKACFKAIKTNQFLHRNRKSQTIDEIMVCHCKPSPDGRLGCGEECLNRMLNIE<br>CLQGTCPAGDLCSNQQFQKRKYVKFERFQSGKKGYGLRLLEDVREGQFLIEYVGEVLDMQS<br>YETRQKEYAFKGQKHFYFMTLNGNEVIDAGAKGNLGRFINHSCEPNCRTEKWMVNGEICVG<br>IFSMQDLKKGQELTFDYNYVRVFGAAAKKCYCGSSHCRGYIGGDPLNGDVIIQSDSDEEYPE<br>LVILDDDESGEGILGATSRTFTDDADEQMPQSFEKVNGYKDLAPDNTQTQSSVSVKLPEREIP<br>PPLLQPTEVLKELSSGISITAVQQEVPAEKKTKSTSPTSSSLSRMSPGGTNSDKTTKHGSGEDK<br>KILPRPRPRMKTSRSSESSKRDKGGIYPGVNKAQVIPVNKLQQQPIKSKGSEKVSPSIETFEGKL<br>NELLDAVGGISKRRDSAKGYLKLLLLTAASRGTDEEGIYSNRDLSMILDALLKTKSKSVLVDII<br>NKNAPFAGMESFKDSVLSFTEHDDYTVHNIARSFRDRWIPKHFRKPWRINREERSESMRSPIN<br>RRFRASQEPRYDHQSPRPAEPAASVTSSKAATPETASVSEGYSEPNSGLPETNGRKRKSRWDQ<br>PSKTKEQRIMTILSQQTDETNGNQDVQDDLPPGFSSPCTDVPDAITAQPQQKFLSRLPVSYGIP<br>LSIVHQFGSPGKEDPTTWSVAPGMPFYPFPPLPPVSHGEFFAKRNVRACSSSMGNLTYSNEILP<br>ATPVTDSTAPTRKRELFSSDIGTTYFRQQKQSVPPWLRNNGGEKTANSPIPGNLTLEKKLNS
**>AtKMT3;2**<br>MQFSCDPDQEGDELPQYEHIYQNDFSYRKHKKQKEEDISICECKFDFGDPDSACGERCLNVIT<br>NTECTPGYCPCGVYCKNQKFQKCEYAKTKLIKCEGRGWGLVALEEIKAGQFIMEYCGEVIS<br>WKEAKKRAQTYETHGVKDAYIISLNASEAIDATKKGSLARFINHSCRPNCETRKWNVLGEVR<br>VGIFAKESISPRTELAYDYNFEWYGGAKVRCLCGAVACSGFLGAKSRGFQEDTYVWEDGDD<br>RYSVDKIPVYDSAEDELTSEPSKNGESNTNEEKEKDISTENHLESTALNIQQQSDSTPTPMEED<br>VVTETVKTETSEDMKLLSQNSQEDSSPKTAIVSRVHGNISKIKSESLPKKRGRPFSGGKTKNV<br>AQKHVDIANVVQLLATKEAQDEVLKYEEVKKEAAVRLSSLYDEIRPAIEEHERDSQDSVATS<br>VAEKWIQASCNKLKAEFDLYSSVIKNIASTPIKPQDTKTKVAEAGNEDHIKLLEAK
**>AtKMT3;3**<br>MLDLGNMSMSASVALTCCPSFLPAASGPELAKSINSPENLAGECNGKHLPMIPPEEEVKDIKI<br>ANGVTAFTRKQNPSDRVKKGFVLDDHVKDWVKRRVASGVSESTCFLPFLVGAKKMVDCLV<br>CHKPVYPGEDLSCSVRGCQGAYHSLCAKESLGFSKSSKFKCPQHECFVCKQRTQWRCVKCP

MAAHDKHSPWSKEILHLKDQPGRAVCWRHPTDWRLDTKHAVAQSEIEEVFCQLPLPYVEEE
FKIDLAWKDSVVKEDPPSYVHIRRNIYLVKKKRDNANDGVGCTNCGPNCDRSCVCRVQCISC
SKGCSCPESCGNRPFRKEKKIKIVKTEHCGWGVEAAESINKEDFIVEYIGEVISDAQCEQRLW
DMKHKGMKDFYMCEIQKDFTIDATFKGNASRFLNHSCNPNCVLEKWQVEGETRVGVFAAR
QIEAGEPLTYDYRFVQFGPEVKCNCGSENCQGYLGTKRKEPNCLVVSWGAKRRRLFHRPIAR
KPQQD

>AtKMT3;4a

MPASKKISDRNHLGQVFDKLLNQIGESEEFELPEWLNKGKPTPYIFIRRNIYLTKKVKRRVED
DGIFCSCSSSSPGSSSTVCGSNCHCGMLFSSCSSSCKCGSECNNKPFQQRHVKKMKLIQTEKC
GSGIVAEEEIEAGEFIIEYVGEVIDDKTCEERLWKMKHRGETNFYLCEITRDMVIDATHKGNK
SRYINHSCNPNTQMQKWIIDGETRIGIFATRGIKKGEHLTYDYQFVQFGADQDCHCGAVGCR
RKLGVKPSKPKIASDEAFNLVAHELAQTLPKVHQNGLVNRHIDAGKSWNNLSQRDTCSRNCI
GVVIRLSRPTSDRCFGLVRHFDEYSRKHSVMFEDGVTEFVDMSREDWEIV

>AtKMT3;4b

MSSSKKGSDRNQIRKSLRKLKKQIGELEKLESPDRLNNVKPIFIKRNIYLKKKLKKKVKDHGIF
CSCSLDPGSSTLCGSDCNCGILLSSCSSSCKCSSECTNKPFQQRHIKKMKLVQTEKCGYGIVAD
EDINSGEFIIEYVGEVIDDKICEERLWKLNHKVETNFYLCQINWNMVIDATHKGNKSRYINHS
CSPNTEMQKWIIDGETRIGIFATRFINKGEQLTYDYQFVQFGADQDCYCGAVCCRKKLGAKP
CKTKNTTLEEAVKPVACKVTWKTPKLLNSEVRETNLDASGQAWNNHSQRKICCRDCIGAYY
TAQMKVLTLVVDIFQVMYEDGVTEIIDMCREVWKVVTA

>GrKMT3;1a

MGSCGNMTFVNEPSRDVASAAAEQHLCSESMGKLVSEQRACVVIDSNGDYASEPGEDENSA
CKRSGDIDCKVGIKGEAQIVGSGTKGLMGGECCENSVCLKKNGGEIADASCSKALVGDMCG
DSVVCLENNQGDLMGDSGLKELMGNANDDTVVCLKDQGESMNDSDSKELMDDRCRDCIV
YSIENQGEKEDGSFPLDLRSDTYRDSTVCSIGNQGENVDGAGSKELIVDICGDAMVCLNDNQ
GENPDCSGPEELMGYVDNTGYSNEIQCKNVDSGLNELKGDRISDHGVGLNENQDDVDIHDS
VTDVCLEKSGPSGEDGTNAAKGSLGLSQDKNTAALSGGIEISTNCEDQMKGDNENVVGLML
KECMGNNQGRICLTGNMGIDDHLDSENDVSQDGEMPIELNTMSTSPKSSVKLDKQDDDERV
NGSILQRILEYGEMKFEEKSDAVERKGTDVLNQILLSENFKGPFELIDATGEHKSKDGMSTYC
SSSEVAMEEKREVLTEVEANICNQMSSIQGSHLASTSIGIGDCRSDCPRQNDLKDSNTIVGPSL
DGESGASPMIESDTCGKISTSCCVETISNLQQTGDSVGSCDGHSQKDDLSSSGLSLERFPKPVE
TKSTDDLCIELLASRRLFDTQKNAQTVGNDISDSSGDGVAEVSEGRTDFLAYTKAETSSEIIIN
AKGKACNSNRDSFEQGANCLCDKSTSLSCQPVDVVDNGLSGRLDPQDLLAKDACAAISSSSS
IDCSGQRENEGKDIIKADCVLETKNCPTTSSSSRKGRQKGKSSRKTPAKRGARNCSSTKLRHP
HESMEFLFKATRRKRSCSSKPARSSIWGLFSNIAKFIEPCPDPSCNEVQNRKPSKARGGRGSGK
RSKNRAGQNRKESSGLSSTLTSCLRLKIKVGKEVAPSNVNTLVAEVVDPSVLIGTSFSNYGKE
TNLQYPTVANIVEDKVELGSEMRFQSKEDQEMVKTCSDVFLTEVKLANKAVRCSENLERLSE
DATDSSLISQSDAVAEASQEAIENKCMDPGTSPDSEVINTIPDAQIGLIHQEESLDTVLNTSGAL
ASPRGARTSKGSKRGKKDNHRSPGAASTRKAKSSKSRRSREKTTGNGLVSIEALTSSSAANSS
RENWIGVPKEATEMENSMDIKDCCSPDVPDTKNTKHFSSSNSKCNQLSKSSRSQGASKGKSR
VSHSVKSRKAKGSKRRGDESKSVSKSKVKEKCSDEEIVARGGKSPVTVGAAGNQISDDNEHL
NTGNSIESANMVNVDLVPDGVMEQHTQPDNAWVRCDDCHKWRRIPVSLVKSIDEAYHWVC
GDNVDKAFADCSIPQEKSNADINAELGISDAEEDGCDGFNYKELEKGFETKRMTVSPPSHFW
RIDSNQFLHRGRKTQTIDEIMVCQCKRPPDGKLGCGDECLNRMLNIECVQGTCPCGDLCSNQ

QFQNHKYAMMKWDRFGKKGFGLRMLENISAGHFLIEYVGEVLDMQAYEARQKEYASRGQ
RHFYFMTLNGSEVIDAYVKGNLGRFINHSCDPNCRTEKWMVNGEICIGLFALRDIKKGEEVTF
DYNYVRVFGAAAKKCHCGSPHCRGYIGGDPLSAEVIVHDDSDEESPEPMMLEDGETWTGFK
DVISRSGSSDGAAMQSVESVITDGVLKPENMPEAEDSVNRSASATSELNTVETEDLKGNFQL
AIQPEEASPVTIASESVQPDGTEEQKAMNKTSCSIQKLDASQDMSDNRLSSDVIDANKKSKSD
AAEDKQVSAKSRPLMKTSRSSSSIKKGKISSNSLSGNKVQTTSNKFQVSTVKPRKFSENSSTCR
FEAVEEKLNELLDSEGGITKRKDASKGYLKLLLLTATSGDSGGGEAIQSNRELSMILDALLKT
KSGHVLTDIINKNGLQMLHNIMKKYRKDFKKIPILRKLLKVLEYLAGRGILTQEHINGGPYCA
GRESFRESILSFTEHDDKQVHQIARNFRDKWIRKPVRKLGYRDKDEGRMEFFRGLDCNRVSA
SHNHWRDQAIRSTEAINCIMQSVVGSTSSDTSTREGGSSLSVCLSQTNSTRIRKRKSRWDQPA
DTEKIDSQSPKKLECSLLSALGQATPDQIEKMNSGDNKCQSFSKGEAINIENGNQRFQQDAPP
GFSSPLNASLVSSTAPSTATGFPPPKVGQLKCPDVVIAHPQKRFISRLPVSYGIPLPIFQQTRLPE
GESLESWAIAPGIPFHPFPPLPPCPPDKKDTRPVSAALSTGINADSKEGQQESRRPSTSCPDESV
ASTAGGNHPDSDIPGTDIQQTLKRTRESSYDLGRKYFRQQKRKGPPWDKSESFGNNHTGEICC
IDVGNINNEPRNSYYSDDINQ

>GrKMT3;1b

MCLCESTALVNEPLSVVASAEQHSCSESMENLVPEPRDCIISDSSGDSTGNRYDDTVVYLEEN
RGESNGDSADYSYENHCENVDCSGLKELLGARIDDHVACLNVSPGKIDVHNSENDQLCLEN
RLFSGKYVPTAINGSSGLSQDEYSACLSSGTEIDTEIYNRIQSVKDSNLTLESIAIAGCRSGCAQ
QNGQNDNNIVRGPLLDGKNCASSMIKSVTSSEISAICCAQTLSSLQGSGDSVSSCDWLNQKDD
MSSRDLSLESFNKAVEAKSIGDTYSKLLASKCCVSSFETLHRAESLCTKQNAQIDNKNFIVLSG
DSVAKVSEERTDIAAGAKVETSSEIMNAGDSFELSENSLCDKLVPLSCHPFDIVENGLSGRLDP
PDCLTNGAYAALNSSSSIDFCGQRQNEGKVVVKADCVSEIKHHPTESSSSRRGGRKGKSSQK
TNAKTRNCRNKLQQPPESIELHFRASRRKRSCSSRPGRSSIQGLFSNITQFLEPCDDPEFNEVQN
QKPSNGRDGQGSRKSCKDQSGQSIKGSGGLSKSSTSCLRFRIKVGKGVGPSNLNSVVAEVVN
LPVSVDTSFSIYGKGTGLQFPKLANVAEDKVGELGIERQFLNKEDQEKVKTCLDASFMDLKL
TNNVSGSAEYLKKYAEDALGDYLVSKPNALAESSGRAIDNKYSGFGTSPDSVVINSIPDAQV
GLIHQEELHDPVLNNSGFLASPGGVKSSMVSKKGKKDNHRSPRTVCLRKAKSSNNCRGRTKT
RDNEFISNKAISSSAGANSSRGNGLGVSEEAMKMDINMDAKACCSHHVPETKKFKNLSSTKY
TLNQLSKSSKSQGVRKERSKVSDSAGSRKGNACKQWGDELKSVSKIKVKEKGSNQEIVTRG
GKHPLTGNHISDDFENSDAGNSSASAYMTNIDSVSDVIKQHRQPDNAWVCCDDCHKWRRIP
VILLNSIDEACRWICGDNMDKTFADCSIPQEKSNADINAELGVSDAEEDGCDGLNYKEFDKG
FNNNRVTVPPPSHFWRIDSNKFLHRGCKTQTIDEIMICHCKRPPDGNLGCGDECLNRMLNIEC
VQDTCPCGELCSNQQFQKRKYAKMMWDRFGRKGFGLRMLESISAGQFLIEYVGEVLDMQA
YEARQKEYASRGQRHFYFMTLNGSEVIDAYVKGNLGRFINHSCDPNCRTEKWMVNGEICIGL
FALRDIKKGEEITFDYNYVRVFGAAAKKCHCGSSHCRGYIGGDSLSEGVIVYDDSDVESPEPM
MLEDGETWNGYANVISRSSPFVGAEMQPVERVITDGVRKLEKMPEAEGSVYHSASASSKLDI
SAEIEDLQGNFQLPIEPEEVSPLTAPYEPVQQDDTIQQKAMKKTSRLIHILDTFLNMSDNKLPS
VFIDANKESKFNTAEDKRVPPKSHPLMKASCLSSSHKKGKLSSNSLNGTKVRMISDKSQVPSF
KLKKFSETSSSCRFEAVEEKLNELLDSEGGITKRKDASKGYLKLLLLTATSGDSCNGEAIQSTR
ELSMILDALLKTKSRLVLTDIIDKNGLQMLHNIMKKYRRDFNKIPVLRKLLKVLEYLARRKIL
TVERINGGPPCAGRESFLESILSFTEHYDKTVHEIARNFRDTWIPKPLRKHSYRDKVERRMEFC
RYLDCNRVSASHNHSREQAIRSTEAITVVEKTTLDTSHEICSSSPTGVCQTNGTKIRKRKSRW
DQPAETEKIDSRSPKKHEYSQLTILGKPTSNHMNKLSRWDKECHDILCKGEAVNVVNGKHRF

QGDAPPGFSSPCSASLVSSTAALTATSFPQPKTCQLKCPEMTIAHPQTRLISRLPVSYGIPLPIVQ
RFGAPKDESVESWVIAPGMPFHPYPPLPPSPCPHGRKDTPPVCAANSIGNNEDAKDEQQDCCR
PATSYPDNSIRSTAHCNEPNSEIPCANIQRTSKRTRESSNDLGKYFRQQKRKGPLWHKSESTGS
KHNNIGGTSFLDVGNVKNDVRNS

>GrKMT3;2

MGGLSIAGLFCQVYVWDPENEDLPQYEHIFQNEFSYRKHKKQKEEDIAICECKFDFSDPDSTC
GERCLNVLTSTECTPGYCPCGVYCKNQKFQKCQYARVTLFKTEGCGWGLLAAEYIKTGQFIV
EYCGEVISWKEAKRRSQAYENQGLKDAFIISLNGSESIDATKKGNLARFINHSCQPNCETRKW
TVLGEIRVGIFAKEDIPIGTELAYDYNFEWYGGAKVRCLCGALNCSGFLGAKSRGFQEDTYL
WEDDDERYSVEKIPLYDSAEDEPATKLLKAVNLNSENDVNTKSEQSITMDVNLKSKHQLEST
IDTVPMEGVDVNTLKIESPKDINLYSQDAQQAFSQKNAMISRIRSNSACRNYHIRSGPMLKKK
SQHYSNGKLKHLSKKQIDLKHLAKLLASKEAQEEVFRYEEMKNEAASQLASLYNDIRPAIEE
HERDNQDSVSTSVAEKWIEASCTKLKIEFDFHSSILRNIVCTPQKACEQVKPCEPEGHGGNND
TEVKLEF

>GrKMT3;3

MPGLGNLSHSSSLSLSPCPNLKLLSVDSADSIDAFDSTKLKTLPVNCKWDQRLVPKRVNCNGS
GIRVLKKVKKGSNGKALQDYLRDWVHRKMESGLPKSCYFLPFLVGAKRLVECLDCHKLIYP
GEEVLCSVRGCQGVYHKTCAEESFRMSNPKKFQCPQHVCFVCRQRLQWRCVCCTMASHDK
CSPWPDAVIHLKDKPGRAICWRHPTNWRLDKKHADPATEIQEIFCQLPLPYMDEEFKLDLTW
RDLIENKLVPPPYVHIKRNVYLVKKKRDDVVDDIGCTSCSSTCSEDCVCRVQCISCSKACRCP
ESCTNRPFSKEKKIRIVKTELCGWGVEAAEPIKKGDFVIEYIGEVIDDAQCEKRLWDMKHKG
VKNFYMCEIRKDFTIDATFKGNTSRFLNHSCDPNCVLEKWQVEGETRVGVFAARSIKVGEPL
TYDYRFVQFGPEVRCHCGASNCQGYLGTKRKIVKLDLCWGSKRRRTSTACLAIITV

>GrKMT3;4

MPATKKSSDCSHIGYAFNKLMKQIGHPVEFELPDCFNKWKDTPYTVIRRNIYLTKRIKRRLED
DGIFCSCTSSSPGSSVCGRDCHCGMLLSSCSSGCSCGSSCLNKPFQHRPVKKLKLIKTEKCGEG
IVADEDIKHGEFVIEYVGEVIDDKTCEERLWNMKHRGETNFYLCEINRDMVIDATYKGNKSR
YINHSCCPNTEMQKWIIDGETRIGIFAMRDIKKGEHLTYDYQFVQFGADQDCHCGAAGCRQK
LGVKPSKSKMSSDATLKLVACQVAVSSPTLKAVLFGNENGGLPVGTSQHDYNQRQLRFRCCI
GEVIKISRPINDSYFGIIKRFDKYSKKHLVMFEDGDVEFLDLSKEDWEIITL

>OsKMT3;1

MEEPDGEARGREDHAAVGRLGGEEGAVGGGGLALLAVPEIGGELGDGGKVCGGQERRLPT
EEDGVRDNGGGSAAELVESAVNVSTPFEGRGQIGGEKESSMQEGSMNMAGEKHGSYHVESA
EPSNLQTCHAPNGGVSNKTLFAPFSEVFSSDNSHMRYLLDKATEGSICEHGDLADSKDDLGG
ATDVKTNTEDLQMVCTKPHCDSEGLSDLHNDSERWPQVVDGVGFTIKGNNELKQVDLIPKIE
AEVSRSVEDDSIPSFSGGIDDSLRKAGCACETLNDMGMSHMANGDLWCNVLYAPLSEGCQS
KDARHIAVMGNKVTQGSQCGQGDLACDGIVLRGGVDVEKSLDDLQMCSKEPQCDNKGFPY
LTEFGVQQPSYGMNVICSKTDPNHQLEKDELLTNTRGEFSSSIHEDSVPSISVSSVDFTFDGNA
GQIGKTSEHRAIMEKVSHGSQRGGVLSCESRSLKESHADENQSSTLEVKTCEEGLQTGQVEPC
HSIVALSDSGKYGTDILPRGGDGLRSMTGANHELVKDDFHPKSDVVVSCPVDEASIPSNYNSP
IDVLLYKEDGLVGEISENRIGVEKLAHDLLGEVMLSFDSRPQTEASGDENQHFWMDVPKGST
ASVCEVENTGTRRSCDPCAEIEFPLQQSREKHVISESPPERDLTSSSHNLPCENEPCYSGRETPA
FCLGHQDSAGLGLESSDCLVQELNTCTSTDDKACSVDFVENGNGSHNQKEVPVIFFRRRNPV
RAASSRNSNFEKCDQINKSGNSTRKSKKVDSVSSLLKSTMIKFPNKTTKGRSGINRPLNSSAW

GSLQKLMDGFNQNCGPSTSRSHQTCLGKEISNRGSSEKKQLSIRKIRTSRCSKYKNTSLSDIGY
LAGELNGQPTCSVRIDTNVSSDALFNSPNGAHKAAQCVEGNHTLKLTSSLTDTQQFGLENVT
QETCPGYIHGECGTSTSERSLNNIVGFSPDSVLDIASVTCESNTSATLDVIVHENPSCPGGLIGG
GLRASALSTSHCENHHASSLMDLEQQVKTVRENDMGEEDVIPSHAMMYNDIGEGKQTLAKS
NTMRKGRNVGKQECRKKDGKKGKNINKNRSSTKISSSEASKLVSFSNDSPSLDPSELLLHTRP
PKFGSCSKVVTSAIHDVGMHGYDNMRPFGIDNDDEGSAFDNVKSLRRKKKDSHGGKKGKV
RDPHGKGRSKKKNIADNTYGDLIPAAELVFKNSSAVSVELPAVVACKTDGASVPPAPAWVC
CDDCEKWRCIPTELADKISKENLRWTCKENEDKTFANCSIPQEKTDDEINAELGLSDASADEA
NGDGSNSKASGEPNFALLRSNLFLHRNRRTQSIDESMVCNCKPPHDDRMGCRDGCLNRILNI
ECTKRTCPCGEHCSNQQFQRRTYAKLGKFHTGKKGYGLQLKEDVSEGRFLIEYVGEVLDITA
YESRQRYYASKGQKHFYFMALNGGEVIDACTKGNLGRFINHSCSPNCRTEKWMVNGEVCIG
IFAMRNIKKGEELTFDYNYVRVSGAAPQKCFCGTAKCRGYIGGDISGADMITQDDAEAGTFE
PMAVQEDAEEVLGANGLSSHGTHLDIVDHEASTKTEDSNDCPSVNPPELESEQQTSGTLFDTS
EPENSLEALSPQDDEDVVRTPVHVSRTVESTSRQFPEYGTRSSEILQRAPCTLDGPKVPSTTNG
IPPSSDLGSHWVPGFHANKKTNVKHHLILNPSSAPIDSEHILGVEGRLNSLLDVNGGISKRKDA
TNGYLKLLLVTAAEGDNAGGTSKSVRDLSLILDALLKTRSNSVLLDIINKNGLQMLHNILKQN
KSDFHRIPIIRKLVKVLEFLASKGILTSEHINGGPRCAGTESFRESMLGLLRHNDMQVQQIARN
FRDRWIQWAPRNISRNEPTEYSRASISAHDIHVISTAGGSFPTSANTMDWKSIRRKRKSRWDY
QPDDHYKMGGLKIQKVCPVQSEFRTGSVGNKLHGNWGTNSSHNDVPVVGSSADGADDEAP
PGFESQQESRPGQACLESGVSPGLYLERYQHNLTISYGIPIAFVEHFGTPEVEGGPCRKNWKV
APGVPFQPFPPLPPYPRGSPCPSTQMSQHEHNSLGHCGRAANRDGRIHRNWRNGARTKFPYN
HQGRRFPNNNQRF

>OsKMT3;2

MEEERMEPPPPPPYIHIETNDFLHRRHKRQKEEDIAVCECQYNLLDPDSACGDRCLNVLTSTE
CTPGYCLCGVYCKNQRFQKSQYAATRLVKTEGRGWGLLADENIMAGQFVMEYCGEVISWK
EAKRRSQAYENQGLTDAYIIYLNADESIDATKKGSLARFINHSCQPNCETRKWNVLGEVRVGI
FAKQDIPIGTELSYDYNFEWFGGAMVRCLCGAGSCSGFLGAKSRGFQEATYLWEDDDDRFS
VENVPLYDSADDEPTSIPKDILIKDEPNTQDGNNNTIQNTGIPIIASSSEFTPMNVEPSIASSNEFT
PMNVEPLNVSSNELTPMTIEPLNAIPMGVDFTQNGSIEYGAQCAEDALQNSTRGVANLQNQS
APRDNNHTELVAVKRRPTLRGGKAKRGMRKQLNVVGICDRLASEVAREEILYCEEMKNEAA
AEIDSLYDEIRPAIEEHERDSQDSVATSLAEKWIEASCCKYKADFDLYASIIKNLASTPLRSKED
AAPTEQNGLMYLENGP

>OsKMT3;3a

MPDLSTVCLPLSPQPPSTAAAAGGELAGEQPAAAAVTTAAAAVSASAEAEAAGEAESASGG
DGAPVLPVECRWSGRVRSFAGAGEGAGAAAVPACPAPRRGGGKKPSSAPSPSSTVATAPAHP
SGRPFEEYVKEWKAKKAALGVPAGRCELPFLTGTPKAVECRLCSKIIHPGEEIKCSVSRCGEL
FHLTCAAEDTTNFIAESFKCPQHGCMVCKQKMFFWRCGRCTVAAHTKCAPWPVIHLKDDQG
SAICWRHPSDWLLQNEEVFCRLPVPYVNEDFKIDSTIRDFTAAVCKPPHFTFIRRNVYLIKKKR
PDSRAEAGCTNCSADSTCKDDCECRGLYMSCSKNCHCSDMCTNKPFRKDKKIKAVKTKRCG
WGAISLEPLEKGDFIIEYVGEVINDATCEQRLWDMKRRGDKNFYMCEISKDFTIDATFKGNTS
RFLNHSCDPNCKLEKWQVDGETRVGVFASRSIQVGEHLTYDYRETAFLQSAMVNHDLKYM
HI

>OsKMT3;3b

AGPPGPAPGTSSSSSSLPSRRRRRPPPRLGRRRGGDDQQPPHPPKASSEALPCAASPPARCRGG
DHQPPHPPEAALEALRGAASPPARRRGGDLHPSHPPEAASEALRGAASPPERRRGGDRQPSHP
TEAASEAPSGSASPPARRRGGDQQPPPAVAVAVTSEGGVGPRRSFRISLRHRVRVVPWVKPP
VARKPKDPAKPPRPSIEALAAEWAKEKAASGAPEEECVLPFLQKDAPKKLDIVKVLGLDGFG
YMDTVILSLKSYRKNIPITVGNFVIVMQSCIRLVLKKVMDAVLDMMQVQQMEAFRRLPLPHT
FQEFNIDPIKKEELDNGTEPPPYKIKIIPTGLYWLAALGLVTAQMSALTNHFADRKRLRSLSGI
LLQTQYCGWGSRALEAIEKDDFVIEFVGEVIDDETCEERLEDMRRRGDKNFYMCKVKKDFVI
DATFKGNDCRFFNHSCEPNCQLQKWQVNGKTRLGVFASKAIEVGEPLTYDYRYLFLSP

>OsKMT3;4

MPRPAKIRKKHENVFDQLIKAIKAPVDFDLPPVLKEWKSNYYVPIKRNAYITRKRVEDDGIFC
SCTPSGSSATCDKDCQCGMLFSCCSSTCKCENKCANKPFQHRTLRKTKLIKTEKCGNGVVAE
EDIKKGEFVIEYVGEVIDDRTCEQRLWKMKRQGDTNFYLCEVSSNMVIDATNKGNMSRFINH
SCEPNTEMQKWTVEGETRVGIFALRDIKTGEELTYDYKFVQFGADQDCHCGSSNCRKMLGIT
KPVNSIVLHNGNLSQDQHVRKKRKTYLENCIGEIVRLWHRRHSMYLAASIYDFNERNGIHTL
LFTDATIEEFDLREEDWDFLPDPDGPEEV

KMT6A

>AtKMT6A;1

MASEASPSSSATRSEPPKDSPAEERGPASKEVSEVIESLKKKLAADRCISIKKRIDENKKNLFAI
TQSFMRSSMERGGSCKDGSDLLVKRQRDSPGMKSGIDESNNNRYVEDGPASSGMVQGSSVP
VKISLRPIKMPDIKRLSPYTTWVFLDRNQRMTEDQSVVGRRRIYYDQTGGEALICSDSEEEAID
DEEEKRDFLEPEDYIIRMTLEQLGLSDSVLAELASFLSRSTSEIKARHGVLMKEKEVSESGDNQ
AESSLLNKDMEGALDSFDNLFCRRCLVFDCRLHGCSQDLIFPAEKPAPWCPPVDENLTCGAN
CYKTLLKSGRFPGYGTIEGKTGTSSDGAGTKTTPTKFSSKLNGRKPKTFPSESASSNEKCALET
SDSENGLQQDTNSDKVSSSPKVKGSGRRVGRKRNKNRVAERVPRKTQKRQKKTEASDSDSI
ASGSCSPSDAKHKDNEDATSSSQKHVKSGNSGKSRKNGTPAEVSNNSVKDDVPVCQSNEVA
SELDAPGSDESLRKEEFMGETVSRGRLATNKLWRPLEKSLFDKGVEIFGMNSCLIARNLLSGF
KSCWEVFQYMTCSENKASFFGGDGLNPDGSSKFDINGNMVNNQVRRRSRFLRRRGKVRRLK
YTWKSAAYHSIRKRITEKKDQPCRQFNPCNCKIACGKECPCLLNGTCCEKYCGCPKSCKNRF
RGCHCAKSQCRSRQCPCFAADRECDPDVCRNCWVIGGDGSLGVPSQRGDNYECRNMKLLL
KQQQRVLLGISDVSGWGAFLKNSVSKHEYLGEYTGELISHKEADKRGKIYDRENCSFLFNLN
DQFVLDAYRKGDKLKFANHSPEPNCYAKVIMVAGDHRVGIFAKERILAGEELFYDYRYEPD
RAPAWAKKPEAPGSKKDENVTPSVGRPKKLA

>AtKMT6A;2

MVTDDSNSSGRIKSHVDDDDDGEEEEDRLEGLENRLSELKRKIQGERVRSIKEKFEANRKKV
DAHVSPFSSAASSRATAEDNGNSNMLSSRMRMPLCKLNGFSHGVGDRDYVPTKDVISASVK
LPIAERIPPYTTWIFLDRNQRMAEDQSVVGRRQIYYEQHGGETLICSDSEEEPEPEEEKREFSEG
EDSIIWLIGQEYGMGEEVQDALCQLLSVDASDILERYNELKLKDKQNTEEFSNSGFKLGISLE
KGLGAALDSFDNLFCRRCLVFDCRLHGCSQPLISASEKQPYWSDYEGDRKPCSKHCYLQLKA
VREVPETCSNFASKAEEKASEEECSKAVSSDVPHAAASGVSLQVEKTDIGIKNVDSSSGVEQE
HGIRGKREVPILKDSNDLPNLSNKKQKTAASDTKMSFVNSVPSLDQALDSTKGDQGGTTDNK
VNRDSEADAKEVGEPIPDNSVHDGGSSICQPHHGSGNGAIIIAEMSETSRPSTEWNPIEKDLYL
KGVEIFGRNSCLIARNLLSGLKTCLDVSNYMRENEVSVFRRSSTPNLLLDDGRTDPGNDNDE
VPPRTRLFRRKGKTRKLKYSTKSAGHPSVWKRIAGGKNQSCKQYTPCGCLSMCGKDCPCLT
NETCCEKYCGCSKSCKNRFRGCHCAKSQCRSRQCPCFAAGRECDPDVCRNCWVSCGDGSLG
EAPRRGEGQCGNMRLLLRQQQRILLGKSDVAGWGAFLKNSVSKNEYLGEYTGELISHHEAD
KRGKIYDRANSSFLFDLNDQYVLDAQRKGDKLKFANHSAKPNCYAKVMFVAGDHRVGIFA
NERIEASEELFYDYRYGPDQAPVWARKPEGSKKDDSAITHRRARKHQSH

>AtKMT6A;3

MEKENHEDDGEGLPPELNQIKEQIEKERFLHIKRKFELRYIPSVATHASHHQSFDLNQPAAED
DNGGDNKSLLSRMQNPLRHFSASSDYNSYEDQGYVLDEDQDYALEEDVPLFLDEDVPLLPS
VKLPIVEKLPRSITWVFTKSSQLMAESDSVIGKRQIYYLNGEALELSSEEDEEDEEEDEEEIKKE
KCEFSEDVDRFIWTVGQDYGLDDLVVRRALAKYLEVDVSDILERYNELKLKNDGTAGEASD
LTSKTITTAFQDFADRRHCRRCMIFDCHMHEKYEPESRSSEDKSSLFEDEDRQPCSEHCYLKV
RSVTEADHVMDNDNSISNKIVVSDPNNTMWTPVEKDLYLKGIEIFGRNSCDVALNILRGLKT
CLEIYNYMREQDQCTMSLDLNKTTQRHNQVTKKVSRKSSRSVRKKSRLRKYARYPPALKKT
TSGEAKFYKHYTPCTCKSKCGQQCPCLTHENCCEKYCGCSKDCNNRFGGCNCAIGQCTNRQ
CPCFAANRECDPDLCRSCPLSCGDGTLGETPVQIQCKNMQFLLQTNKKILIGKSDVHGWGAF
TWDSLKKNEYLGEYTGELITHDEANERGRIEDRIGSSYLFTLNDQLEIDARRKGNEFKFLNHS

ARPNCYAKLMIVRGDQRIGLFAERAIEEGEELFFDYCYGPEHADWSRGREPRKTGASKRSKE
ARPAR

>GrKMT6A;1a

MYLLKFSSICLSQMHINFIILAIGLFIFTQMTTDEKTPTVKEVLLIIDSLKTQLTADRSVYVKKR
LEENRQKLGGITYHLYKLSNERRSSWISDTDSAPDLLTKRQKDALGMQNGIDASNGDKDRYS
CQESSTAVLMGSSIPVKNAVRPIKLAEVKKLPPYTTWIFLDRNQRMTEDQSVVGRRRIYYDQ
NGGEALICSDSEEEVIEEDEEKRDFVESEDFIVRMTIKQVGLSDPVLESLAQCLSRSPCEVKAR
YETLMKEEKDAGASKNGDVEVENWNSFLEKDLEAALDSFDNLFCRRCLVFDCRLHGCSQDL
IFPQADKQTPWNHPDEENVPCGLHCYRTVLKLERNGTVSSPIDPEEKLNSSSDGVGARTSSYK
KSSGSSARRKVKSCHSENASSNAKNLSESSDSEIGPRHEDASPIPQLSPSKNKIAGKSGILKRNS
KRVAERVLICMRKRQKKMAASASGSAVSGGVSPIDIKLQSDVQKENEDVTSSSQNVKPPNTG
RSRRKEWSLGVQGEFSEVPSSEMINGLAQATSNGGLRKEEFLDENLCEQAPNDDKSWKAIEK
GLFEKGVEIFGNNSCLIARNLLNGLKTCWEVFQYMTCSGNKLACHAADGVMSLLDGYSKFD
LNGSVGNNEVRRRSRFLRRRGRVRRLKYTWKSAAYHSIRKRITERKDQPCRQYNPCSCQTAC
GKQCSCLLNGTCCEKYCGCPKSCKNRFRGCHCAKSQCRSRQCPCFAADRECDPDVCRNCWV
SCGDGTGTLGVPPQRGDNYECRNMKLLLKQQQRVLLGRSDVSGWGAFLKNSVGKHEYLGE
YTGELISHREADKRGKIYDRENSSFLFNLNDQFVLDAYRKGDKLKFANHSPDPNCYAKVIMV
AGDHRVGIFAKERINAGEELFYDYRYEPDRAPAWARKPEASGSKKEEVAPSSGRAKKLA

>GrKMT6A;1b

MTAKHSRSASADRSEPPKDSSMTQIEGKTLSVKEILSVIDSLKKQVAVDRSLSVKTRLEENKQ
KLVGITSHLYKLSKERRSSWIIDTDSASDLLTKRQKDALGMQNGIDASNGDKDGYSYQEPST
AVLMGSSIPVKNAVRPIKLTEVKKLPPYTTWIFLDRNQRMTEDQSVVGRRRIYYDQNGGEAL
ICSDSEEELLEEDEEKKDFVESEDFILRMAIKEVGLSDPVLESLAQCLSRSPADVKARYETLMK
EDTGASKNRDTEEQNWNSFLDKDLEAALDSFDNLFCRRCLVFDCRLHGCSQDLIFPADKQTP
WNRTDDENAPCGLHCYRLVLKSERNDTVRSPMNPEDKSNSSSDGVAAQISSSKKSAGPSTRR
KAKSSQSESASSNAKNLSESSDSEIRPRHEDSSPIPQLSPSKNKIAGKSGILKRNSKRVAERVLIC
MRKRQKKMEASESDSLVSGGVSPTDMRLRSNPRKENEDATSSSQKDVKSSNTGRSRRKDWP
LKGVQGEIPYSETVNDLAQTSSNGCLRNEEFVDENLCKQELSDDKSWKAIEKGLFDKGLQIF
GRNSCLIARNLLNGLKTCWEVFLYMTCFDNKLACHAADGVLSLLEGCSKFDLNGAMGNNE
VRRRSRFLRRRGRVRRLKYTWKSAAYHSIRKRITERKDQPCRQYNPCSCQTACGKQCSCLLN
GTCCEKYCGCPKSCKNRFRGCHCAKSQCRSRQCPCFAADRECDPDVCRNCWVSCGDGTGSL
GVPPQRGDNYECRNMKLLLKQQQRVLLGRSDVSGWGAFLKNNVGKHEYLGEYTGELISHR
EADKRGKIYDRENSSFLFNLNDQFVLDAYRKGDKLKFANHSPEPNCYAKVIMVAGDHRVGIF
AKERINAGEELFYDYRYEPDRAPAWARKPEASGSKKEDGAPSSGRAKKLA

>GrKMT6A;3

MVSKGSDPSTKSKKSLEECSSGGVGNLTHKLNQLKRQIQAERIASIKEKVEKNRKKLESHISEI
LSATSSRNVLCVEENGFGKMLSSRIQIPLFKYAGFAQGSGDRDYSNGHEVVSSTSVKLPYVEK
LPPYTTWIFLDKNQRMAEDQSVVGRRRIYYDQHGSEALICSDSEEDIAEPEEEKHEFSEVEDRI
LWNVCQEYGLGEETLAAVSQFIGVTGSEIEERHGMLREKYSDQNIKDSEDCRSEKGISLDKSL
SAALDSFDNLFCRRCLLFDCRLHGCSQTLINPSEKQPYWSEYEDDRKPCSDQCYLRLRAVKD
VAEGSGVNALHGVKTTLEEKDKVASSDAKEQKTNVDADLMQDERGISEEEGPVTLEGINDS
EGAGKALTSVMSSIPIDNHENSGKRKASQESNRPLDDLLHCSDSSQDSSCKKQKTLLVLDVA
RESSEAIPSHASAQSSKSRNYQVRTLLENETQITAKNNQNESGERGLETSTCSASASKTKDNTR
NGAKDVLKVPELKWSSSEWKPIERELCLKGVEIFGRNSCLIARNLLSGLKTCLEVSSYMCDG

GSSTLNRSIMTSSFLEENGKSESDFMEQEMSARPRLLRRKGRTRKLKYSWKSAGHPSIWKRIA
DGKNQSCKQYTPCGCQSMCGKQCPCLNNGTCCEKYCGCSKSCKNRFRGCHCAKSQCRSRQ
CPCFAAGRECDPDVCRNCWVSCGGDSLGEPPKQGDGQCGNMRLLLRQQQRILLAKSDVAG
WGAFLKNSVNKNDYLGEYTGELISHTEADKRGKIYDRANSSFLFDLNDQYVLDAYRKGDKL
KFANHSSNPNCYAKVILVAGDHRVGIFAKERIEASEELFYDYRYGPDQAPAWARKPEGSKRD
ETSASQGRAKKHQSH

>OsKMT6A;1a

MASSSSKASDSSSQRPKRPDQGPSGKDAAGLVALHGKLAQLKRQVQSTRLAAIKERVEANR
KALQVHTCALFDVAAAAEVASRGAEGGNALSRGAAEGHRRFVGWDSASGPGERELVHVQE
ENLVAGTLVLSSSGGSGASHRTVVQLVKLPVVDKIPPYTTWIFLDKNQRMADDQSVGRRRIY
YDPIVNEALICSESDDDVPEPEEEKHVFTEGEDQLIWKATQDHGLSREVLNVLCQFVDATPSEI
EERSEVLFEKYEKQSQSSYKTDLQLFLDKTMDVALDSFDNLFCRRCLVFDCRLHGCSQNLVF
PSEKQPYGHELDENKRPCGDQCYLRRREVYQDTCNDDRNACTTYNMDSRSSSLKVSATILSE
SEDSNRDEDNIKSTSIVETSRSKITNSEYADKSVTPPPGDASETENVSPDMPLRTLGRRKISKHA
SKSNDHSPDKRQKIYSSPFPFAMSVLNKQSVPEIGETCPDSIESAVDQLPSLDDPNKKISTKDM
CAGSTTNTTENTLRDNNNNLFISNKEHSISHWSALERDLYLKGIEIFGKNSCLIARNLLSGLKT
CMEVASYMYNNGAAMAKRPLSGKSILGDFAEAEQGYMEQDLVARTRICRRKGRARKLKYT
WKSAGHPTVRKRIGDGKQWYTQYNPCGCQQMCGKDCACVENGTCCEKYCGCSKSCKNRF
RGCHCAKSQCRSRQCPCFAASRECDPDVCRNCWVSCGDGSLGEPLARGDGYQCGNMKLLL
KQQQRILLGKSDVAGWGAFIKNPVNRNDYLGEYTGELISHREADKRGKIYDRANSSFLFDLN
EQYVLDAYRKGDKLKFANHSSNPNCYAKVMLVAGDHRVGIYAKDRIEASEELFYDYRYGP
DQAPAWARRPEGSKKDEASVSHHRAHKVAR

>OsKMT6A;1b

MAGDSRNEPMFCEEGSSESGYVLCVIDSLKKKITSDRFVYIQKRVEENSIKLSPITLHSHNLSK
NRQTSTSNSTDLVSNLLTKRKEDALCAVNSRESSPDESEGANCQDECSSTVIVGGNLSARNSV
RPIRLPEVATLPPYTTWIFLDRNQRMQEDQSVLGRRRIYYDTNCGEALICSDSEDEAVEDEEE
KKEFKDSEDCIIRMTIQECGMSDAVLETLARDIERAPDDIKARYEILQGEKPEGSSKKVSELNV
KMEDVYGDKDLDAALDSFDNLFCRRCLVFDCKLHGCSQDLVFPTEKQAPLCSSDEGTPCGIH
CYKLVSKPDAIMEIDSHLLVDVEEPTSDNLKDQIGSNKKKLGSSGQKTKSQQSESSSTARVSS
ESSESEVQLLSNKSPQHSPGLSKNKLGAKGGIKKSTNRRIAERILMSVKKGQQEMSPDSNSIV
NGCHWPRDMKLRSDTRSGIKDSVVSSQCNSPSTRSFRKKGTLQMENNSSFVDAQSDSMEDT
NNEHSATDGCDSSRKEECVDESICRQEAHGRSWKVIEQGLLLKGLEIFGKNSCLIARNLLGGM
KTCTDVFQYMNYIENSSASGALSGVDSLVKGYMKGNELRTRSRFVRRRGRVRRLKYTWKT
AGYHFIRKRITERKDQPCRQYTPCGCQSACGKQCPCLTNGTCCEKYCGCPKMCKNRFRGCH
CAKSQCRSRQCPCFAADRECDPDVCRNCWVGCGDGTLGVPNQRGDNYECRNMKLLLKQQ
QRVLLGRSDVSGWGAFLKNSVGKHEYLGEYTGELISHKEADKRGKIYDRENSSFLFNLNNEY
VLDAYRMGDKLKFANHSPDPNCYAKVIMVAGDHRVGIFAKERISAGEELFYDYRYEPDRAP
AWARKPEGPGAKDDAQPSTGRAKKLAH

KMT6B

>AtKMT6B;1

MATWNASSPAASPCSSRRRTKAPARRPSSESPPPRKMKSMAEIMAKSVPVVEQEEEEDEDSY
SNVTCEKCGSGEGDDELLLCDKCDRGFHMKCLRPIVVRVPIGTWLCVDCSDQRPVRKETRK
RRRSCSLTVKKRRRKLLPLVPSEDPDQRLAQMGTLASALTALGIKYSDGLNYVPGMAPRSAN
QSKLEKGGMQVLCKEDLETLEQCQSMYRRGECPPLVVVFDPLEGYTVEADGPIKDLTFIAEY
TGDVDYLKNREKDDCDSIMTLLLSEDPSKTLVICPDKFGNISRFINGINNHNPVAKKKQNCKC
VRYSINGECRVLLVATRDISKGERLYYDYNGYEHEYPTHHFL

>AtKMT6B;2

MVAVRRRRTQASNPRSEPPQHMSDHDSDSDWDTVCEECSSGKQPAKLLLCDKCDKGFHLFC
LRPILVSVPKGSWFCPSCSKHQIPKSFPLIQTKIIDFFRIKRSPDSSQISSSSDSIGKKRKKTSLVM
SKKKRRLLPYNPSNDPQRRLEQMASLATALRASNTKFSNELTYVSGKAPRSANQAAFEKGG
MQVLSKEGVETLALCKKMMDLGECPPLMVVFDPYEGFTVEADRFIKDWTIITEYVGDVDYL
SNREDDYDGDSMMTLLHASDPSQCLVICPDRRSNIARFISGINNHSPEGRKKQNLKCVRFNIN
GEARVLLVANRDISKGERLYYDYNGYEHEYPTEHFV

>GrKMT6B;1

MAPATIMAGARRLVSSRRRTEAPRRRPSPSTPPKKLRPMSEIMARAKYAVVERADYSDIICEQ
CGSGERPGELLLCDKCDKGFHMRCLRPIVVRIPIGSWLCPKCSGHRRVRTFSQKRIIDFFKIQK
SGDGKKKCNLSQDTRKRRRRPLVLLKKRRRLLPFIPSEDPNQRLKQMGSLASALTAMQMEFS
DDLTYSSDMAPRSANQAKFENGGMQVLSREDMETLELCRSMSRRGECPPFIVVFDSCEGYLY
HEIICTAAVMLLNCLFSIFILIMWCSYTVEADAQIKDMTFIAEYTGDVDYIKNRENDDCDSMM
TLLLATNPSESLVICPDKCGNIARFINGINNHTPEGKKKQNCKCVRYSVNGECRVLLVATRDI
AKGERLYYDYNGYEHEYPTHHFV

>GrKMT6B;2

MAVAKTTTMILRRRTRAPRRYSEDEDGLGCDDVYCERCGSGDFGSKLLLCDKCDKGYHLFC
LRPILVSVPKGSWFCPSCTNIKQPQLFPLVQTKIVDFFRIRRSSESMENQSNIKKRKRACSLAVS
KRKRKLLAYNPTEDPQRRLEQMASLATALKASGTEYSDGLTYRPGMALRSANCAALEKGG
MQILPKEDIETLNLCKKMMEKGECPPLMVVFDPVEGFTVQADRYIKDLTIITEYVGDVDYLK
NRENDDGDSMMTLLHASNPSKSLVICPDKRSNIARFVNGINNFSPDGRKKQNVKCVRYNVN
GECRVLLIANRDIRKGERLYYDYNGYEHEYPTEHFV

>OsKMT6B;1a

MGRRALPPSSSSSSSSSTTTTSPELRRKRTAAPPPPPSPRRYRSISDVMRRSLPVDAAPPVARAY
ESTRCDVCGSGERDEELLLCDGCDRGRHTFCLRPIAARVPTGPWFCPPCAPRSKPVKRFPMTQ
TKIVDFFRIQKGAEDAEAEKYGLFQDVKKRRKRSLVMHKKRRRILPYVPTEDKVQRLKQMA
SLATAMTSSKMKFSNELTYMPGMAGRSCNQATLEEGGMQILPKEDKETIELCRTMQKRGEC
PPLLVVFDSREGFTVQADADIKDMTFIAEYTGDVDFLENRANDDGDSIMTLLLTEDPSKRLVI
CPDKRGNISRFINGINNHTLDGKKKKNIKCVRYDIDGESHVLLVACRDIACGEKLYYDYNGY
EHEYPTHHFV

>OsKMT6B;1b

MGPATPLRRRTRARPAATRAEGGSGGDGDDDDVRCEACGSGESAAELLLCDGCDRGLHIFC
LRPILPRVPAGDWFCPSCIHSASLPAEFPLVQTKIVDFFKIQRGPAAALAAAAESSEGKKRKRK
VGGIRLVSKKKRKLLPFNPSDDPARRLRQMASLATALTATGAVFSNELTYVPGMAPRAANR
AALESGGMQVLPKEDVETLNLCKRMMARGEWPPLLVVYDPVEGFTVEADRFIKDLTIITEYV

GDVDYLTRREHDDGDSMMTLLSAATPSRSLVICPDKRSNIARFINGINNHTPDGRKKQNLKC
VRFDVGGECRVLLVANRDISKGERLYYDYNGSEHEYPTHHFV

KMT7

>AtKMT7;1

MSDGGVACMPLLNIMEKLPIVEKTTLCGGNESKTAATTENGHTSIATKVPESQPANKPSASSQ
PVKKKRIVKVIRKVVKRRPKQPQKQADEQLKDQPPSQVVQLPAESQLQIKEQDKKSEFKGGT
SGVKEVENGGDSGFKDEVEEGELGTLKLHEDLENGEISPVKSLQKSEIEKGEIVGESWKKDEP
TKGEFSHLKYHKGYVERRDFSADKNWKGGKEEREFRSWRDPSDEIEKGEFIPDRWQKMDTG
KDDHSYIRSRRNGVDREKTWKYEYEYERTPPGGRFVNEDIYHQREFRSGLDRTTRISSKIVIEE
NLHKNEYNNSSNFVKEYSSTGNRLKRHGAEPDSIERKHSYADYGDYGSSKCRKLSDDCSRSL
HSDHYSQHSAERLYRDSYPSKNSSLEKYPRKHQDASFPAKAFSDKHGHSPSRSDWSPHDRSR
YHENRDRSPYARERSPYIFEKSSHARKRSPRDRRHHDYRRSPSYSEWSPHDRSRPSDRRDYIP
NFMEDTQSDRNRRNGHREISRKSGVRERRDCQTGTELEIKHKYKESNGKESTSSSKELQGKNI
LYNNSLLVEKNSVCDSSKIPVPCATGKEPVQVGEAPTEELPSMEVDMDICDTPPHEPMASDSS
LGKWFYLDYYGTEHGPARLSDLKALMEQGILFSDHMIKHSDNNRWVTIENATSLRSTXNFPS
VVSDAVTQLVNPPEAPGNLLEDIADTTEAVCIEQGAGDSLPELVSVRTLPDGKEIFVENREDF
QIDMRVENLLDGRTITPGREFETLGEALKVNVEFEETRRCVTSEGVVGMFRPMKRAIEEFKSD
DAYGSESDEIGSWFSGRWSCKGGDWIRQDEASQDRYYKKKIVLNDGFPLCLMQKSGHEDPR
WHHKDDLYYPLSSSRLELPLWAFSVVDERNQTRGVKASLLSVVRLNSLVVNDQVPPIPDPRA
KVRSKERCPSRPARPSPASSDSKRESVESHSQSTASTGQDSQGLWKTDTSVNTPRDRLCTVDD
LQLHIGDWFYTDGAGQEQGPLSFSELQKLVEKGFIKSHSSVFRKSDKIWVPVTSITKSPETIAM
LRGKTPALPSACQGLVVSETQDFKYSEMDTSLNSFHGVHPQFLGYFRGKLHQLVMKTFKSR
DFSAAINDVVDSWIHARQPKKESEKYMYQSSELNSCYTKRARLMAGESGEDSEMEDTQMFQ
KDELTFEDLCGDLTFNIEGNRSAGTVGIYWGLLDGHALARVFHMLRYDVKSLAFASMTCRH
WKATINSYKDISRQVDLSSLGPSCTDSRLRSIMNTYNKEKIDSIILVGCTNVTASMLEEILRLHP
RISSVDITGCSQFGDLTVNYKNVSWLRCQNTRSGELHSRIRSLKQTTDVAKSKGLGGDTDDF
GNLKDYFDRVEKRDSANQLFRRSLYKRSKLYDARRSSAILSRDARIRRWAIKKSEHGYKRVE
EFLASSLRGIMKQNTFDFFALKVSQIEEKMKNGYYVSHGLRSVKEDISRMCREAIKGRNRGG
SKDMNRIIALFIQLATRLEEVSMITSSYGRDELMKSWQDGSGLSSATKYNKKLSKTVAEKKY
MSRTSDTFGVNGASDYGEYASDREIKRRLSKLNRKSFSSESDTSSELSDNGKSDNYSSASASE
SESDIRSEGRSQDLRIEKYFTADDSFDSVTEEREWGARMTKASLVPPVTRKYEVIEKYAIVAD
EEEVQRKMRVSLPEDYGEKLNAQRNGIEELDMELPEVKEYKPRKLLGDEVLEQEVYGIDPYT
HNLLLDSMPGELDWSLQDKHSFIEDVVLRTLNRQVRLFTGSGSTPMVFPLRPVIEELKESARE
ECDIRTMKMCQGVLKEIESRSDDKYVSYRKGLGVVCNKEGGFGEEDFVVEFLGEVYPVWK
WFEKQDGIRSLQENKTDPAPEFYNIYLERPKGDADGYDLVVVDAMHMANYASRICHSCRPN
CEAKVTAVDGHYQIGIYSVRAIEYGEEITFDYNSVTESKEEYEASVCLCGSQVCRGSYLNLTG
EGAFQKVLKDWHGLLERHRLMLEACVLNSVSEEDYLELGRAGLGSCLLGGLPDWMIAYSA
RLVRFINFERTKLPEEILKHNLEEKRKYFSDIHLDVEKSDAEVQAEGVYNQRLQNLAVTLDKV
RYVMRHVFGDPKNAPPPLERLTPEETVSFVWNGDGSLVDELLQSLSPHLEEGPLNELRSKIHG
HDPSGSADVLKELQRSLLWLRDEIRDLPCTYKCRNDAAADLIHIYAYTKCFFKVREYQSFISS
PVHISPLDLGAKYADKLGESIKEYRKTYGENYCLGQLIYWYNQTNTDPDLTLVKATRGCLSL
PDVASFYAKAQKPSKHRVYGPKTVKTMVSQMSKQPQRPWPKDKIWTFKSTPRVFGSPMFDA
VLNNSSSLDRELLQWLRNRRHVFQATWDS

>GrKMT7;1

MGDGVA CMPLQQHQHQHQHIM ERFPVTEKTLCPNNELTTKPVNLKDNAQQQQQQQPQEQQ
QQEQQQQQPQPQLPRKKKKLVKVKKVVVVKKKVVVGAAAAAAAAAAAATSQKSELVVKA
KTEA GLKSSKEIDKGDNSGQKEEVEEGELGTLKWPREGENGEVGTDKSKNGEIEKGEITSEK
CRKGEVVKEEIVREVKGELEKEETVSKKKGEVMNGEIVTGKWRKGEVAKGEMVLEKGRKA
EPEKGEFGSWRGAKDDLEKGEFIPDRWHKGDLMKDEYSYSKYRKYELGKEKSWKYEMERT
PPSGKYSVDDLYHRKEFSRSTLHGRSSSRWETSQDRTSRISSKIVDEEGLYKSEYSNGKNHGR
EYPSSGNRPKRHGTDSDSGDRKHYGDYGDYANSKCRRLSDDFGRNSHPELYSRHSVERFYK
NSSSSRISSLEKYTSRHHESSLSSRVVYDKCGRSPAYSERSPRDRVRNYDHRDRSPIRRERSPW
DRSPYTCEKSPYARDRSVYSRERSPYDRSRHHDHRIRSPINAGRSPEDRPRFHDRRDRTPSYLE
RSPHDRSKTKNQRDTSKKGAINEKRGSQYGSKGQEDKVSRRDHSGRDSHSSAKESQDRISVH
NLNGSDEKNGVCESHKEDQSPTPSVNCQEPPLLVDGAPPEELQSMEEDMDICDTPPHIPLVAE
SAVGKWIYLDVFGIERGPSKLCDLKELVEEGVLLSDHLIKHLDSDRWVTVENAASPLLTASFP
SIVSDSVTQLVSPPEAPGNLLIETGDLKPLGTHSGDETMSFQDDSAATSDSLEDLHIDERVGAL
LDGINIIPGKELEIVGEALQMTFDDAEWEVWGSSDGFPWLLSRTGDWHDKVTEELSSYSDTN
AKEAAEPRAVAISDCSSCADSSDWFSGRWSCKGGDWKRNEEATQDRSSRKKLVLNDGYPLC
LMPKSGYEDPRWHMKDDLYYPSHSKRLDLPPWAFSIAEERNDCNDISRSNQIKPSAVRGVKG
TMLPVVRINACVVQDQGSFVSAPRTKTRVKERHSSRSSRSHSTTSDVKKSSAESDSLSKAVND
QRLKGSWKFAPINTPKDHVCTIDELQLHLGEWYYLDGAGHERGPSSFSELQFLVDQGVIPKY
SSAFRKYDQMWVPVTSAAGSLEVTAWNRPGNVASSADSSGTTLLDSQGVAVSDNNTSSSSF
HRLHPQFIGYTCGKLHKLVMKSFKSREFAAAINEVLDPWISAKQPKKEMDKHIYQKTDSGKR
ARMMINGSEEEYDIEDELQSIRKDDFAFEDLCGDVTFHEQESACSVTEMGNWGLLDGHVLA
RVFHFLRSDMKSLVFASLTCKHWRAAVRFYKGIARQVDLSSLGPNCSDSIAQKILNCYNKERI
NSMVLIGCTNISSITLEDVLQVFPSLSYIDIRGCSQFGELIVKFPNLRWFKSTSLHAMTISDESNS
KIRTLKQITEKTSSGLKTGLGNAIDDFGELKSYFESVDRRDSANQLFRQSLYRRSKLFDARKSS
SILSREARIRRWAIKKSENGYKRMEEFLASSLRDIMKENTSDFFVPKVAEIEEKMKNGYYIGH
GLGYVKEDISRMCRDAIKTKNRGGARDMNRIITLFIQLATRLEEGAKITSSYERDELLKSWKD
DSPTGFSKYKKKLGKAVTERKYMNKSNGTSFANGAFDYGEYASDREIRKRLSKLNRKSLDSE
SETSDELDRSSEDGKSESEIESTASDTESELDFKPEGRSGESRGDGYFMAGDSFDSMADDREW
GARMTKASLVPPVTRKYEVIDQYVVVADEEDVRRKMQVSLPEDYAEKLNAQKTGTEELDM
ELPEVKDYKPRKELGDEVIEQEVYGIDPYTHNLLLDSMPEELEWPLEDKQSFIEDVLLRTLNK
QVRQFTGTGNTPMMYPLKPIVEEIKRVAEVDCDKRTMKMCQGILKAIDDRPDDNYVAYRKG
LGVLCNKEGGFREEDFVVEFLGEVYPVWKWFEKQDGIRLLQNNSKDPAPEFYNIYLERPKGD
AGGYDLVVVDAMHKANYASRICHSCHPNCEAKVTAVDGQYQIGIYALRAIRYGEEITFDYNS
VTESKEEYEASVCLCGSQVCRGSYLNLTGEGAFQKVLKEWHGILDRQQLMLEACELNSVSEE
DYLELGRAGLGSCLLGGLPDWLVAYSARVVRFINFERTKLPEQILRHNLEEKRKYCIDISLDA
ERNDAEIQAEGVYNQRLQNLAITLDKVRYVMRCVFGDPKKAPPIERLSPEEAVSFLWKGEG
SLVEELLQSMAPHVEDETLNDLRSKIQVHDPSWSDNILKELQKSLLWLRDEVRNLPCTYKCR
HDAAADLIHIYAYTKCFIRVREYKAVTSPPVYISPLDLSPKYSDKFTGLQEYCKTYGENYCLG
QLVFWYNQTSVDPDSSLFRASRGCLSLPDIGCFYAKVQKPSRHRVYGPKTVKFMLSWMEKQ
PQRPWPKDRIWTFKGSPRIFGSPMLDAVLNNSSLDREMVQWLKHRPAKFQAMWDR

>OsKMT7;1

MPDKGERGHHTTSNNHHSHSHSHRKHHAELEEGELLNGEADNSSSRDLERSMPPKKWRKVL
AASSAAEVEPGEIVMPSKKARKNGELEKGEIAPERQRKDKSDKSGRKSNKDEVEPGEVAPPD
KKQDRDHNKKLGSSAQVRDDGSKKGSSRDSDEEPGEIRPESSSTGSARKSRATEPENSNHKH
QADTCDQTGSKSRRKGEAKSSGRHLSGRNRDISPMTRDRHERSPGILGRFPHDRLRHDRSPSR
LEPSPRDRGRHYDNRDRSPYISPRHRMRPSHYRDNTPSRGEMHHHRDNTPSRVDSSPRRSQH
EDFRDRSPSRRDKSPSERGRTTESHEAGKKSRGAKLENNSLEKAQHKSKSTKQSTKSKSSSNG
KDMDICDTPPHTTSSAPGPTEPPASDVGKWFYLDHYGIEQGPSKLADLKKLVEDGYLLSDHLI
KHADSNRWVTVENAASPLVPSEFPSVYSDVSTQLVSPPEAPGNLLDEAREEASGTDHEQMKE
ASAEEQEDFYIDDRVDALMDGSIMVDGQELEILGGAGDASSVILLFKPNGEVFHAVHVVGPG
FYKKPFFPQLLPLPHGCTNVVAFCHFQSTGKAKVLNQFSLHLCMVSFNYVDAIHLLGRVTIKF
NVFFHLSRFQVKLERDDGTKRSTEFPDSRTAHIYGVVPAERDTYQPHIESSEWYSGRWSCKG
GDWKRNDDFSQDKPYRKKLVLNEGYPLCQMPKGNHEDPRWGCKDDLYYPLRAKKLDLPL
WAFSSTEENDDTVDDASKSGVMPGRSGQTKQPPKGVKGTTLPVVKINARVVKDQSSSELRIK
PKVADRPPSRSSRSHSIGTDRSSTHEGSSHSKKHHEHDSQSLHKSKSVPNIPKDHVCTVEELSV
KVGDWYYLDGTGHERGPFSYSELQELAKKGTILEGSSVFRKIDNTWLPVLKDLKSGCSARNG
EAGSSTSALTHSNQSNFHEMHPQFVGYTRGKLHELVMKYFKSRELTLAINENFVQEDGGSTK
RARLLPDQSDEYTDMSEDILASQKDDCCFEDLFEGAAHVKESPLNSRTESESWGLLNEHVLA
RIFHFLRADVKSLISSAATCSWWNTAAKYYRSVCRFIDLSSLGPQCTDNVFHDIMAGYDMQN
IRTLVLTGCSNLSSLALAEVLKRFPHISYVHIQGCSQLGDLKNKFQHVKWIKSSLNPDASYQKI
RSLKQIDDGSNSTSKAGRILTSQMGGSDELDGYFADISNRESSTLSFGQGFYKRSKWLDIRKSS
AVLSRDAQMRRLMQRKAENSYRKMEEFVINKLKEIMKSSRFDFFVPKVAKIEVRLKNGYYA
RHGFSYIKNDIRSMCRDALRYKGRSDLGDMKQIVVAFIQLAKKLENPRLISDRDGTAVQKDS
SDMSQYSSDLKLKKKQSKTMSERRGANWTTAGADPSSRAFDREIKRSLSKLKKRDIDSGSET
SDDDDGYSEGDETESETTVSDTESDLDVNSGAWDLKGNGMKLFESSESLTDDRGWGARMT
KASLVPPVTRKYEVIEKYLIVADEEEVLRKMRVALPDDYSEKLLSQKNGTENLELPEVKDYQ
PRKVPGDEVLEQEVYGIDPYTHNLLLEMMPTELDWPSSDKHTFVEELLLNTLNKQVRQFTGS
GNTPMVYPLKPVIEEIQKSAEESGDRRTSKMCLGMLKAMRNHPEYNYVAYRKGLGVVCNK
TGGFGVDDFVIEFFGEVYPSWRWYEKQDGIKHIQNNSDDQAPEFYNIMLERPKGDRDGYDL
VFVDAMHKANYASRICHSCNPNCEAKVTAVDGHYQIGIYTVRPIAEGEEITFDYNSVTESKEE
HEASVCLCGSQICRGSYLNFSGEGAFEKVLMEFHGVLDRHSLLLQACEANSVSQQDLIDLGR
AGLGTCLLAGLPGWLVAYTAHLVRFIFFERQKLPHEIFKHNVDEKRQFFTDINMDSEKNDAE
VQAEGVLNSRLQNLTHTLDKVRYVMRCIFGDPKNAPPPLVRLTGRSLVSAIWKGEGSLVDEL
LESMEPHVEEDVLTDLKAKIRAHDPSGSEDIEGEIRSSLLWLRDELRTLSCTYKCRHDAAADL
IHMYAYTKCFFRVRDYKTVKSPPVLISPLDLGPKYADKLGPGFQEYCKTYPENYCLGQLIYW
YSQNAEPESRLTRARKGCMSLPDVSSFYVKSVKPTQERVYGSRTVRFMLARMENQAQRPWP
KDRIWVFKSDPRFFGTPMMDAVLNNSPLDKEMVHWLKTRSNVFLG

RBCMT

>AtRBCMT;1

MDLEHQTMETFLRWAAEIGISDSIDSSRFRDSCLGHSLSVSDFPDAGGRGLGAARELKKGELV
LKVPRKALMTTESIIAKDLKLSDAVNLHNSLSSTQILSVCLLYEMSKEKKSFWYPYLFHIPRD
YDLLATFGNFEKQALQVEDAVWATEKATAKCQSEWKEAGSLMKELELKPKFRSFQAWLWA
SATISSRTLHVPWDSAGCLCPVGDLFNYDAPGDYSNTPQGPESANNVEEAGLVVETHSERLT
DGGFEEDVNAYCLYARRNYQLGEQVLLCYGTYTNLELLEHYGFMLEENSNDKVFIPLETSLF
SLASSWPKDSLYIHQDGKLSFALISTLRLWLIPQSQRDKSVMRLVYAGSQISVKNEILVMKW
MSEKCGSVLRDLPTSVTEDTVLLHNIDKLQDPELRLEQKETEAFGSEVRAFLDANCLWDVTV
LSGKPIEFSRKTSRMLSKWRWSVQWRLSYKRTLADCISYCNEKMNNLLGTQDRLRDL

>AtRBCMT;2

MEIRAAEDIEIRTDLFPPLSPLASSLYDSFLSSHCSSCFSLLPPSPPQPLYCSAACSLTDSFTNSPQ
FPPEITPILPSDIRTSLHLLNSTAVDTSSSPHRLNNLLTNHHLLMADPSISVAIHHAANFIATVIRS
NRKNTELEEAAICAVLTNAVEVHDSNGLALGIALYNSSFSWINHSCSPNSCYRFVNNRTSYHD
VHVTNTETSSNLELQEQVCGTSLNSGNGNGPKLIVRSIKRIKSGEEITVSYIDLLQPTGLRQSDL
WSKYRFMCNCGRCAASPPAYVDSILEGVLTLESEKTTVGHFDGSTNKDEAVGKMNDYIQEAI
DDFLSDNIDPKTCCEMIESVLHHGIQFKEDSQPHCLRLHACHYVALNAYITLATAYRIRSIDSE
TGIVCDMSRISAAYSLFLAGVSHHLFCAERSFAISAAKFWKNAGELLFDLAPKLLMELSVESD
VKCTKCLMLETSNSHRDIKEKSRQILSCVRDISQVTWSFLTRGCPYLEKFRSPVDFSLTRTNGE
REESSKDQTVNVLLLSSHCLLYADLLTDLCYGQKSHLVSRFRL

>AtRBCMT;3

MAISEEEAKLERFLDWLQVNGGELRGCNIKYSDSLKGFGIFASTSTQASDEVLLVVPLDLAITP
MRVLQDPLLGPECQKMFEQGQVDDRFLMILFLTLERLRINSSWKPYLDMLPTRFGNPLWFSD
DDILELKGTNLYHATELQKKKLLSLYHDKVEVLVTKLLILDGDSESKVSFEHFLWANSVFWS
RALNIPLPHSFVFPQSQDDTGECTSTSESPETAPVNSNEEKGKSLTSAQPAPSVGSGDTIWVEG
LVPGIDFCNHDLKPVATWEVDGIGSVSRVPFSMYLLSVAQRPIPKKEISISYGNKGNEELLYLY
GFVIDNNPDDYLMIKEMLVNFVLTSVVTFNNGFIQVHYPVEAIPSIPFSDSKGQLLEAQNAQL
RCLLPKSVLNHGFFPRTTSVIRESDEKETVRSCNFSWSGKRKMPTYMNKLVFPEDFMTGLRTI
AMQEEEIYKVSAMLEELVESRQGEQPSETEVRMAVWEACGDSGALQLLVDLLNSKMMKLE
ENSGTEEQDARLLEEACVLESHEESRDLDGRRMSRNKWSSVVYRRGQKQLTRLLLKEAEHA
LHLALSSDH

>AtRBCMT;4

MSASVAVVSGFLRIPSIQKSQNPSFLFSRPKKSLVRPISASSSELPENVRNFWKWLRDQGVVSG
KSVAEPAVVPEGLGLVARRDIGRNEVVLEIPKRLWINPETVTASKIGPLCGGLKPWVSVALFL
IREKYEEESSWRVYLDMLPQSTDSTVFWSEEELAELKGTQLLSTTLGVKEYVENEFLKLEQEI
LLPNKDLFSSRITLDDFIWAFGILKSRAFSRLRGQNLVLIPLADLINHNPAIKTEDYAYEIKGAG
LFSRDLLFSLKSPVYVKAGEQVYIQYDLNKSNAELALDYGFVESNPKRNSYTLTIEIPESDPFF
GDKLDIAESNKMGETGYFDIVDGQTLPAGMLQYLRLVALGGPDAFLLESIFNNTIWGHLELP
VSRTNEELICRVVRDACKSALSGFDTTIEEDEKLLDKGKLEPRLEMALKIRIGEKRVLQQIDQI
FKDRELELDILEYYQERRLKDLGLVGEQGDIIFWETK

>AtRBCMT;5

MANSKMAIASLAAQIRPFTCLAASLPSRLAPHPPDLIRWIKREGGFVHHAVKLSQETQFGIGLI
STEQISPGTDLISLPPHVPLRFESDDSSSSSSLLSALARRVPEELWAMKLGLRLLQERANADSF

WWPYISNLPETYTVPIFFPGEDIKNLQYAPLLHQVNKRCRFLLEFEQEIRRTLEDVKASDHPFS
GQDVNASALGWTMSAVSTRAFRLHGNKKLQGGSSDDVPMMLPLIDMCNHSFKPNARIIQEQ
NGADSNTLVKVVAETEVKENDPLLLNYGCLSNDFFLLDYGFVIESNPYDTIELKYDEQLMDA
ASMAAGVSSPKFSSPAPWQHQLLSQLNLAGEMPNLKVTIGGPEPVEGRLLAALRILLCGELVE
VEKHDSDTLKSLSAVAPFGIANEIAVFRTVIALCVIALSHFPTKIMEDEAIIKQGVSATAELSIK
YRIQKKSVIIDVMKDLTRRVKLLSSKETPTAA

>AtRBCMT;6

MVGALNIMSETGDLCVELPKDDPFYHHKKKFLSCKGLCVKETLNLSGSLSQQLLNAALEKLL
HFGRIVNLDKVEVYFGEDACTPAGIYSVRNEISALSWILSLIPVSCKMQTQVDTFEALRAALK
GRINEVVGAEKEKARVVDSYRCEKESKLVEWGQDNGVKTKLQIAQIDGYGRGAIASEDLKF
GDVALEIPVSSIISEEYVYNSDMYPILETFDGITSETMLLLWTMREKHNLDSKFKPYFDSLQEN
FCTGLSFGVDAIMELDGTLLLDEIMQAKELLRERYDELIPLLSNHREVFPPELYTWEHYLWAC
ELYYSNSMQIKFPDGKLKTCLIPVAGFLNHSIYPHIVKYGKVDIETSSLKFPVSRPCNKGEQCF
LSYGNYSSHLLTFYGFLPKGDNPYDVIPLDFDVIDDEDIETEFSWTTHMLRGTWLSSNHNIFH
YGLPTPLLNYLRKAHGLLWKNLEVEIGVLENLQSTFDDMMQNLGDADSIDRENADWDVKL
AMEFKERQRKIVSSILDSCSAGIKLVQESITNPPV

>AtRBCMT;7

MAKACLLQSTLLPAYSPLHKLRNQNITLSFSPLPLSRCRPGIHCSVSAGETTIQSMEEAPKISW
GCEIDSLENATSLQNWLSDSGLPPQKMAIDRVDIGERGLVASQNLRKGEKLLFVPPSLVISAD
SEWTNAEAGEVMKRYDVPDWPLLATYLISEASLQKSSRWFNYISALPRQPYSLLYWTRTELD
MYLEASQIRERAIERITNVVGTYEDLRSRIFSKHPQLFPKEVFNDETFKWSFGILFSRLVRLPSM
DGRFALVPWADMLNHNCEVETFLDYDKSSKGVVFTTDRPYQPGEQVFISYGNKSNGELLLS
YGFVPREGTNPSDSVELALSLRKNDKCYEEKLDALKKHGLSTPQCFPVRITGWPMELMAYA
YLVVSPPDMRNNFEEMAKAASNKTSTKNDLKYPEIEEDALQFILDSCETSISKYSRFLKESGS
MDLDITSPKQLNRKAFLKQLAVDLSTSERRILYRAQYILRRRLRDIRSGELKALRLFSGLRNFF
K

>AtRBCMT;8

MLFCISTVKLFGFQQRRNVSSLAKRFSLAGKLTLELQTQASLDNNFLPWLERIAGAKITNTLSI
GKSTYGRSLFASKVIYAGDCMLKVPFNAQITPDELPSDIRVLLSNEVGNIGMLAAVLIREKKM
GQKSRWVPYISRLPQPAEMHSSIFWGEDELSMIRCSAVHQETVKQKAQIEKDFSFVAQAFKQ
HCPIVTERPDLEDFMYAYALVGSRAWENSKRISLIPFADFMNHDGLSASIVLRDEDNQLSEVT
ADRNYSPGDEVFIKYGEFSNATLMLDFGFTFPYNIHDEVQIQMDVPNDDPLRNMKLGLLQTH
HTRTVKDINIFHSSCDTFTIKEVKSAIGKGKGIPQSLRAFARVLCCIIPQELNDLSKEAAQNDGR
LARLPFKDGNRELEAHKILLSHINRLIEDHSVCIKEMEECYFVSQRFAVRRQMARDLLYGELR
VLRSAAEWLNHYCTTLLSETM

>AtRBCMT;9

MASSVSLQFLTNTFISKPQGFCNGIVSAPRPRSNLLRDRQNGVRPIKVASIETQPFPLFQSPASE
ESSSSELETADPDFYKIGYVRSVRAYGVEFKEGPDGFGVYASKDIEPRRRARVIMEIPLELMITI
RQKHPWMFFPDIVPIGHPIFDIINSTDPEIDWDIRLACLLLFSFDRDDHFWRLYGDFLPAADECS
SLLLATEEDLAELQDPDLVSTIRQQQKRILDFWEKNWHSGVPLKIKRLAEDPERFIWAVSMA
QTRCISMQTRVGALVQELNMMIPYADMLNHSFEPNCFLHWRPKDRMLEVMSNAGQDIKKG
EEMTINYMPGQKNNMLMERYGFSTPVNPWDAIKFSGDSRIHLNSFLSVFNIYGLPEEYYHDSE
LSRGDTFVDGAVIAAARTLPTWSDIDLPPIPSAERKAVKELQDECRKMLAEYPTTAEQDQKLL
DSMSEARTTFATAVKYRMHRKMFIGKIIKALDIYQERLLY

>GrRBCMT;1a

MEMRAKQDIEIGDDITPPLLPLSFSLHDSFLSSHCSSCFSPLSFPPSPHHYGSLYCSAPCSSSHSPI
SSSSAESFLPLTCPLSSDLRTALRLLLSLPSTCPHLHRFTNGLLTNYLKLTSSPEFAAQIRQGAIA
MAAARKLRKGLSLDQSDDVLLEEAVLCLVVTNAVEVQDESGRSLGIAVYDPSFSWINHSCSP
NACYRFIVSPPNATSFGEDSASALRIVPSVSEENFGVCSCSEYNKGTEGYKYGPKIMVRSIKRI
KKGEEVCVSYTDLLQPKAMRQSYLWFNHQFTCSCSRCTVFPSTFVDHALEEILASNPSFSSAG
LDLNLYRDEANKKLSHYVDETNTEFLSVGDPESCCKKLESVLEGGFHVEQLESEDGKSRLNC
KFHPFNHIALNSYMTLASAYRIRSSDFLAFQSKTDESQLKAFEMSRISAGYSLLLAGATHYLFC
SESSLIVSAVNFWKQAGESLLTIAGSSVWNLLGLPKSELSTVVKYKCSECSLMDIFGAKSILNQ
AERTNFENISSDFLACVRSASPKFWRFLIHGCHYLETFKDPFDFRWLAHAHCVAEDVDFIKED
SNCEHHAEWYTNARTHIYKVGMHCLVYGVILAHICYGQNSHLTTHVLNHVENFVH

>GrRBCMT;1b

MEKLKCVVPERLKRRVEESHIDDLPSLSSSLLQLFLSLPQFHQLISELAAEPSTQAGLCGKNEG
A[ALDLKQKGNQCYSSGDHSQALRCYSQLQALRLAPIDAHHSGKNLV][ATLYLNRASLFHK
MGLPMESLRDCSRALEISPCYPKVWYRRGKVNATLGNYEDSVHDLSVAKDMEP]SIGGKKQI
ERELDMLARHHEKTTAKPASQKSVEIPDVPSEIKLQCVKTPDKGRGMVSQFDIPQASLIHAEE
PYAVVILKHCRETHCHYCLNELPADTIPCISCSLPLYCSQHCQVQAGGPIHIPDTMVGSNFKPT
LECIPEHKHECQGVHWPAILPSDVVLAGRVVVKTMEQKGQFTDVPNLSETLGLCESYSKMSP
ESKLELHIYSIVLLFCLHHSFSSELSINGDSASQIVILLSQIRVNSIAVVRMRSNTNDSYDQQDW
FQNFSFGEAEAASSVEQVRVGQALYKAASLFNHSCLPNIHAYFMSRTLLLRTTEFVSGGCALE
LSYGPQVGQWDCKDRLRFLEEEYFFRCWCCGCSEVNESDLVISGFCCVNPNCSGVVLDNLV
ANCEKQKPKVPETISNKSHLQVHELNDIDIKKVAQISLDQTQSSFHVDSGYCLKCGSYCNFAS
RSKEVKKAWTDFRRLQDSIASKDTCSTNLPGALRSLGLLRSILHAYNKGIAEAEDILAKAFCFI
GDFEPARDHCKASIEILEKLYGPNHIAIGYELAKLASIQLALGDCTAVYNINRLSAIFSTYYGPH
AGIIFPYLLVLERERSRIIQ

>GrRBCMT;4

MAPSLFTLSPPSSTFPSSSATLKPFSSFSTKPTLSIHFVKRPISVSATADPPPQLQTFWQWLQDQ
GVVSAKSPVRPGMVPEGLGLIAKKNISRNEVVLEIPNRFWINQEAVAASEIGTVCSGLKPWVS
VALFLIRERFRQDSKWRVYLDILPELTDSTVFWSEEELAELRGTQLLSTTLGVKEYVQNEFLK
VEEEIILPNKQLFPAPITSDDFFWAFGILRSRAFSRLRGQNLVLIPIADLINHSPNITTEDYAWEI
KGAGLFSRDLLFSLRSPVSVKAGEQVLIQYDLDKSNAELALDYGFIESKSERNAYTLTLEISES
DPFFGDKLDIAETNGLGETAYFDIVLGRPLPPVLIPYLRLVALGGTDAFLLESIFRNTIWGHLD
LPVSRANEELICQVVRDACKSALSGYHTTIEEDEKLMEDENLDSRLKIAVGIRAGEKKVLQQI
DEIFKGRESELDELEYYQERRLKDLGLVGEQGEIIFWETN

>GrRBCMT;5

MAAAKLLIPTLSHFRPLTCAAAVSASNPARLVPHPPDLLKWIKREGGFVHEAVTITQDTTYGL
GLVASGGIPKGSDLIVLPEHVPLKFQSDKDDEADSVSLPLSNRVPEELWAMKLGLKLLQERA
KVGSFWWPYISNLPETYTVPIFFSGEDIKNLQYAPLLYQVNKRCRFLLEFEQEVKNVLKNLKP
SEHPFGGQDVDASSLGWAMSAVSSRAFRLYGKKLPNGTHSDIPMMLPLIDMCNHSFNSNARI
LQEQDAGNPKMLIKVVAEREIKQSDPLLLNYGCLSNDFFLLDYGFVIPSNPYDHIELKYDGAL
MDAASMAAGVSSPNFSSPAPWQQEILFQLNLDGEVPNLKVTIGGPELVEGRLLAALRVLLSN
DREMVQRYDLSVLKSLSAEGPLGVANEVAAFRTIIALCVIALGHFPTKIMDDESLLKQGVSVS
TELAIQFRMQKKSVIIDVMRDLTKRVKLLLSKETTTA

>GrRBCMT;6a

MREGYSILLELSDADPFFDKKKRLLNDMGFDVKEIVHIKSSLDRDSLSTTLNQMLQIARIIHLD
EVELYFGQIGGLGFYSPRNEMEAQNFILSLIKSALSHHVRMQTHALQDLRDALINRIHELGTEF
KVENKIDINYKCDKEKCLLQWAENNGAKTRLQIAYVEGAGRGAIAMEDMEVGDIAMEIPASI
TISEDLVYKSDMYQVLEKIDGMSSETMSLLWCMKERHNCNSQFKMYFDTLPENFNTGLSFG
VEAIMALDGTLLFEEIMQAKEHLRVQYDELFPALSKDHPDIFPPELYTWEQFLWACELWYSN
SMKIMFADGNLKTCLIPVAGFLNHSLHPHIVHYGKIDSTTNSLKFRLSRPCSAGEQCCLSYGN
FSGSHLITFYGFLPQGDNPYDVIPLDFEVDQVDTMVECPLSNWTTHMVRGTWLSENQNIFYY
GLPSPLLDCLRSARSQMPFTKTLIKANMEVDKEILEDLQSTFNAMMENLSDMDSVDRESSSW
DVKLASQFKEMQRRIVSSILTSCSTGIKLVEHELSKCKAEE

>GrRBCMT;6b

MVGNGKMNPSSEAEEEGVKSLLKWAAKYGIEVSESCSSLGLGHCLGVSCFPGAGGRGVAAL
RPISKGELLLKVPKSALITTDFLLSRDETLSLALKAHPSLSSTKVFTVCLLYEINKGKASPWHP
YFLHLPRSYSILAAFGELETQALQVDYAIWAAQKAVTKAKYEWEQAFTLMKELKLKPPLLTF
RAWIWATGTISSRTLHIPWDEAGCLCPVGDFFNYAAPTEDPDSFENVENWQNEHAKDDLDIH
HSQRLTDGGYEEDVAAYCFYAKKNYNVGEQVLLSYGTYTNLELLEYYGFLLEDNPNEKVFI
PLDLDMHSLSCWPKESLYIHQNGRPSFALMCALRLWATPPRQRKSIGHLAYSGCPISKGNEIY
VMKWIGKKCDALLKEMPTSVEEDKSLVHLIDKMVEYENLREWVKEASAVFGGEFGDNNILK
AAYGVEGDNELTSLVRTKMLIDRWKLAVQWRLMYKTVVARCISYCTDIINSLSTQ

>GrRBCMT;7a

MKNETRRLRTVKTAHIMITVENDQSPIRASRLGFSSFATFPSLSDKVIPSQFPAMAEASRIFHST
LIPTLSLQLSKPCYNSHVYPSLSLKKAPRPVQCSVSTSETKSTSSNATQEVPWGCDIDSLENAE
ALQKWLSNSGLPPQKMAIDKVAVGERGLVALKNIRKGEKLLFVPPSLFITADSEWSSPEAGL
VLKQYSVPDWPLLATYLISEASAQKSSRWCNYISALPRQPYSLLYWTRAELDRYLEASQIRQR
AIERVTDVIGTYNDLRLRIFSKYPDIFPEEVFNMETFRWSFGILFSRLVRLPSMDGKVALVPWA
DMLNHSCEVETFLDYDKSSQGVVFTTDRAYQPGEQVFISYGKKSNGELLLSYGFVPKEGANP
SDSVELPLSLKKSDKCYKEKLEALRKHGLSTSQCYPIQITGWPLELMAYAYLAVSPPSMSKQF
DEIAAAASNKSTIKKDLRYPDIEEKALQFILDSCESSISKYSKFLQVSGSMDLDVTTPKQLNRR
VFLKQLAVDLCTSEQRILFRAQYILRRRLRDMRSGELRALRIFDGLRNIFK

>GrRBCMT;7b

MAEASRTFHATLFSLPTFSPRLHFKLSYSHSHPSLHLKRARSVQRSISETKTMSSDTTLPWGCD
IDSLENAEALQKWLSDSGLPPQKVGINKVEMGKRGLVALKKINRGEKLLFVPPSLLITSDSDW
SSPEVGHVLKQHNVADLPLLATYLISEANLQKSSRWSNYISSLPRQPYSLLYWTRSELDRYLK
ASQIRLRAIERIADITGTFDDLRRRILSKHPHLFPKEIFNLVTFRWSFGILLSRLIYLSSMDGKVA
LVPWADMLNHSCEVETYLNYDKSSQAVVFTTDRAYQSGEQVFISYGKKSNAELLLSYGFVP
KEGTNLNDSVELPLSLKISDKCYKQKLKALKKHGLSASQCYTIQISGWPLELMAYAYLTVSPP
SMSKQFEEMAAMASNESIIRKDLRYPEIEEKALQFILDNCESSISKYSKFLKESGSMDLDITSQE
LQNRGVFLKQLAVDLCISEQKILHRAQYILKRRLRDMRSGELRA

>GrRBCMT;7c

MLKIGGAAPLNPPLPPQPYRHSCSRRNYFSSNLMASRRLRAFKRWMKSQGIQCSDTLDFIDCP
QQGISVGALSDLHEGDVVAKIPKTACLTIKTSGAREMIESAGLDGHLGLSVALMYEKSLAQD
SPWAGYLQLLPPQECLPLVWTLGEVDSLLSGTELHKAIKEDKTLMYEDWKENILPLVYSAPQ
SLNPSSFSVKEYFAARSLIASRSFQIDEYHGFGMVPLADLFNHKTGAEDVHFTSVSPNQEYED
DVDSENGDNNELSKISGHDKRDSTCENSYIHSDSESDYSSVTGEDPMMLQMIMVREVKSGDE

VFNTYGSLGNAALLHRYGFTEANNPFDIVNIDLELVLKWGCSLFSSRYCRARLSLWRRINISG
SVSENSEYFEVSSDGEPELELLTLLYIMLLPDDTCHKLDISICTADKVSANIGMILSEKHDITWS
TSSEISKDLLLTEKVCRALLALADIRESCYGSKSIDEDVEALKRCCMTERKLYHSLMLRISERR
ILEKFRTYATAGAQIQTFQDANGTSTTRKRLKKH

>GrRBCMT;8

MSNSQSSACLIHASRLPCTPRTLKHDVLTTTMLHCGSRISKLCRSPVTPPFNFPRRLLIKFEFSS
LFQAKGLNTYDEESDEFLPWLERKARTKISSMLSIGKSAYGRSLFASENIRTGDCILKVPYSVE
ITPDNLLPKIRAILSDKIGTVSKLAIILLVERKMGQSSGWAPYICCLPQHGEIHSTIFWSEYELN
MICQSSVYQETVNQKAKIEKDFAAVVPALQQFPDLESITLQDFMCAYFSVTSRAWESTKGLS
LVIADRNYAPGEEVLINYGKFPNAMLLLDFGFTVPYNIHDQVQIQLSIPHDDNLREMKLELLQ
HLTPKIKDAVGFNCPEDTFIIKEVRSPRGKGKGLPQALRAFARLLCCNSSQELSDLAMEAAQI
DGRLARRPLKDSRREFKAHKMLSSHITQLMQKYDTAIKSLPVNSPSMSNTFTLRRQMAHDLL
TGELRVLKSASIWLNNYCAVLKSTSNCRSQWKNRI

>GrRBCMT;9a

MAASFVPLHHVSGCFTYACPEREYRRTHFHSHGWSPAPATKYRLRPINASVGTPSFPLFQPTP
VQESPSQLEPADPDFYKIGYVRSMRAYGIEFKEGPDGFGVYASKDVEPLRRARVIMEIPLELM
LTIRQKLPWMFFPDIVPLGHPIFDIINSTNPETDWDLRLACLLLYAFDKEDNFWQLYGDFLPSA
DECTSLLLATEDDLSQLQDPDLVSTMKNQQLRALEFWEKNWHSGVPLKIKRLARDPERFIWA
VSIAQSRCINMQVRIGALVQDANMLIPYADMLNHSFQPNCFLHWRFKDRMVEVMINAGQRI
RKGEEMTINYMNGQQNQMLMQRYGFSSSVNPWDSIPFSGNAHVHLDSFLSVFNISGLPGEYY
HNSQLADKGDNFVDGAVIAAARTLPTWSDGDVPLIPSMERKAVKELQEECQQILAQFPTTSA
QDQKLLESMPEARRTLETAIKYRLHRKLFIEKVIEALDIYQERILF

>GrRBCMT;9b

MVPSRSWVVTSTSDQVLFEIETPSFPLFQPTPVQESPSQLEPADPDFYKIGYVRSMRAYGIEFK
EGPDGFGVYASKDVEPLRCARVIMEIPLELMLTIRQKLPWMFFPDIVPLDWDLRLACLLLYAF
DKEDNFWQLYGDFLPSADECTSLLLATEDDLSQLQDPDLVSTMKNQQLRALEFWEKNWHS
GVPLKIKRLARDPERFIWAVSIAQSRCINMQVRIGALVQDANMLIPYAGLPNCFLHWRFKDR
MVEVMINAGQRIRKGEEMTINYMNGQQNQMLMQRYGFSSSVFSIFPSMPEARRTLETAIKYR
LHRKLFIEKVIEALDIYQERILF

>OsRBCMT;3

MAAAAAAAAVPGDAKLDSFLQWLQANGADLRGCTIRRCGREGYGVFSTAAEAGATDEVV
MVVPLDLAITPMRVLQDPLVGPRCRALFEEGGVDDRLLVMLFLMVERLRPSSLWKPYLDML
PSTFGSSIWFTEDELAELEGTTLHRATVMQRKSLQTLFDNKVKGLVGELLNVDESGSSIEVRF
EDFLWANSIFWTRALNIPLPRFYVFPESLDEKRANIGDDCGDSSLSAPQGTGTAITAKNISGND
NPKSSNTESIWVEGLVPGIDFCNHNVKALATWEVDSMGHVTGCPSSMYLVLADKSFVKAET
EICINYGNKGNEELLYLYGFVIDNNPDDYLMIHYPVEALRQVQSADIKMKLLEIQNAELRCLL
PRSLLENGFFGSCSGENKENKNNTSPFSSYSWSGQRKVPSYIEKIVFSQEFISTLRTIALQEHEL
EHTASLLGEIGSNEDRDDELRSAIWEVSGDNGALSLLVDLLRVKMTELEEGTGTEASDSQLLE
KFDLSDSEDATSDESNETKSKVNIRTCIVYRRGQKQLTKLFLREAEHLLELSSKEEN

>OsRBCMT;4a

MAAAIHHHHLLPPRLLSVHPQPPRLRLRRPLPRRAAASGAAAGTSSSTAAAPPPTDAALQEFR
RWVSSHGADAGAGAAAPAAVPEGGLGLVAARDLPRGEVLAEVPKKLWLDADAVAASDLG
GAVGRGGLRPWVAVALLLLREAARGAGSPWAPYLAILPRQTDSTIFWSEEELLEIQGTQLLST
TMGVKEYVQSEFESVEAEIISENRELFPGTVTFNDFLWAFGILRSRVFAELRGDKLALIPFADL

VNHSDDITSKESSWEIKGKGLFGRDVVFSLRTPVNVKSGEQIYIQYDLDKSNAELALDYGFTE
SNSSRDAYTLTLEISESDPFYDDKLDIAELNGMGETAYFDIVLGESLPPQMLPYLRLLCLGGTD
AFLLEALFRNAVWGHLELPVSQDNEEAICQVIRNACKSALGAYHTTIEEDEELLGSENLQPRL
QIAVEVRAGEKKVLQQIDDIFKQREEELDGLEYYQERRLKDIGLVGDNGEIIFWES

>OsRBCMT;4b

MEASASTSTARRLRAFRRWMRDHGVVCSNALRLDAAEDGGGGVYVRALAALREGDLVATI
PRGACLTPRTSGAAEAIEAAELGGPLALAVAVMYERARGAESPWDAYLRLIPEREPVPLVWP
ADEAERLLAGTELDKIVKQDRQFICEDWKECIEPLILSGELEVDPDDFSLENYFSAKSLLSSRSF
RIDSYHGSGMVPLADLFNHKTGGEHVHFTSVLEASDSDSEDGEDPNNASADEQSTIENSADIP
SGDDDEDLEMIVVRDVNEGEEVFNTYGTMGNAALLHRYGFTEMDNSYDIVNIDLALVTKW
CSSKYSRRYARARVSLWHNLGYSGCTSQDADYFEISYDGEPQLELLILLYIISLKSDAYDKLA
SVAHDLIGDDEVDSISSVLKVVRVTSSNQHPDISGLEKLPDVKKLLLNESVCSALVSLVDMRE
SLYGSNTLEDDRQKLQACSSVNERNLYHSLVLRVSERTILHKLKKHASSWSKTKKRKQL

>OsRBCMT;5

MAAAAAAGATPATARKALLTTTATLLSSSLARSRRSLSCSAAAASAAPRIAPQPPDLLRWVQ
REGGFVHPALRVVDHPEHGLGVSAAAAEGDIPPGDVLIALPGRLPLRLRRPAGAADAVLVQL
ADQVPEELWAMRLGLRLLQERAKSDSFWWPYIANLPETFTVPIFFPGEDIKNLQYAPLLHQV
NKRCRFLLEFEKEVKHKLGTVPLEDHPFCGQDVNSSSLGWAMSAASTRAFRLHGEIPMLLPLI
DMCNHSFNPNARIVQEGNVDSPDMSVKVVAETKIDQNAAVTLNYGCYPNDFFLLDYGFVITS
NSYDQVELSYDGTLLDAASMAAGVSSPNFSAPAKWQQDILSQLNLYGEGAILKVSIGGPEIV
DGRLLAALRVIIAADPDAVSGHDLKTLMSLKEKAPLGPAVEASALRTVLALCTFALQHFHTKI
MEDEAILKGEPPLTTELAVQFRLQKKLLLLDVIQNLSRRIKMLALDKSTV

>OsRBCMT;6

MAAAAAEVAAAGGGGEMVVVRLPPLSQDDPLFQDKKRILDSRNLSCLFQVPNSCSAADAFK
VLDRIIQAARIAHMDELELYFTGDDDFGPLSTRNELESLNLLLKILNTLLLTANVGAMGVLQV
LRDEILIRLRSLELEDNDQMVVQIRNQNMEDSLLKWGEQHGVKTKLQIAFFEGAGRGMVASE
NIDVGDIALEIPESSIISEELLCQSGMFLALKDLDSITTETMLLLWSIRERYNPSSKFKIYFEALP
ANFNTGLSFGIDALAALEGTLLFDELMQARQHLRQQYDELFPMLCIKFPDIFKQDVYTWDNF
LWACELWYSNSMMVVLSSGKLTTCLIPIAGLLNHSVSPHILNYGRVDKVTKSLKFPLSRPCK
AGEQCFLSYGKHPGSHLITFYGFLPRDNPYDVIPLDLDTSVDEEDSSSPSVTTSQTSHMVRGT
WLSRLRGPPTYGLPHRLVSHLHAILGCNQNESAPEADNKENDRMVLETLLSIFTPMLEGLGEP
DDFDRENACWDVNLALDYKDLQRRIVLSIVTSCTSGLAMLDS

>OsRBCMT;7a

MATAAALALHTQFRPPRSPRRLRQHLALPSGVLIRSPVRASAASASASAPAQREAAAAGVPW
GCEIESLESAVSLERWLTDSGLPEQRLGIQRVDVGERGLVALKNIRKGEKLLFVPPSLVITADS
EWGCPEVGNVLKRNSVPDWPLIATYLISEASLESSSRWSSYIAALPRQPYSLLYWTRPELDAY
LVASPIRERAIQRITDVVGTYNDLRDRIFSKHSDLFPEEVYNLETFRWSFGILFSRLVRLPSMDG
RVALVPWADMLNHSPEVETFLDYDKSSGGIVFTTDRSYQPGEQVFISYGKKSSGELLLSYGFV
PKEGTNPNDSVELLVSLNKSDKCYKEKLQALKRNGLSEFESFPLRVTGWPVELMAYAFLVVS
PPEMSQRFEEMAVAASNKSPSKPGLNYPELEEQALQFILDCCESNIAKYTKFLEGSSGSLQLST
NSKQANRTLLLKQLARDLCISERRILYRTQYILRRRLRDMRGGELKALSLFNGLRKLFK

>OsRBCMT;7b

MEALLRWAAELGVSDSPSAPSPSSCLGRSVLIADFPDAGGRGLAAARDLRRGELVLRAPRAA
LLTSGRVMDDDPRIASSVASHLPRLSSVQTLIICLLSEVGKGKSSNWYLYLSQLPSYYTILATF
NDFETEALQVDEAIWVAQKALRGIRSDWEEATPLMKGLGFKPKLLMFKSWIWAFATVSSRT
LHIAWDDAGCLCPIGDLFNYAAPNDDNSSTDEDRDDMMHQETNKMLDQTDFDSSEKLTDG
GYEDVNEYRLYARKRYRKGEQVLLAYGTYTNLELLEHYGFLLGENPNEKIYIPLDLDLCMIG
SWPRDSLYILPNGHPSFALLCALRLWTTPRNRRKALSHQIYSGSLLSVENELEILKWLVKKCK
ETLQQLPTTIEFDDNLLVLLCKLQNSTSCITEMNRSIFEQEFAPFFRFHGFKLDCSIHSKLPVRL
LRSLERWGLAVQWRCNYKRTLTKCIVHCKSLVHELSLQQNQQ

>OsRBCMT;9

MATPAASPLLLPLPLPLPASTFPPRRAVPCARRLVLRPPRAGRPRLRDPPPAAPPPAAEEVGEE
EEDDDAPPLRLLEPPQEDDPFPPEMEPADPDFYRIGYARMMRAYGVEFLEGPDGMAVYASR
DVDPLRRARVIMEIPLELMLTITQKRPWMFFPDIIPLGHPIFDIIESTDPETDWDLRLACLLLYA
FDVEDNFWQLYGDFLPSVDECTSLLLAPKHKTIPLKLKRLAPDHERFLWALSIVQSRSVNLKL
RMGAFLQDANVLVPYADMLNHSPDANCFLHWRFKDRMVEVMIKAGHAVKKGDEMTIDYM
SGVNSSFMERYGFSSPTNPWELINFSSDAKIHLDSFLSVFNIAGLHDELYHNAALTSGENNFVD
GGVVAAARTLPTWSEGDVPAIPSLERKSAQALQEECHTMLESFSTTIQQDQEILDSDGHIRRT
REIAIKYRLHRKLLLQKIIDALDIYQDKIL

S-ET

>AtS-ET;1

MRGEQFELEEDRDGPLELLQSLRSKATELLLREEWEESIKVYTEFIDLSRRQVSSTGGSDPDPD
SIAKLRKSLCL[ALCNRAEARARLRDFLEAMRDCDQALEIEKTH][FKTLLCKGKVLLGLSKYS
LALECFKTALLDPQ]ASDNLETVTVYIEKCKKLEFQAKTGAFDLSDWILSEFRGKCPELAEFIG
SIEIKKSELSGRGLFATKNIVAGTLVLVTKAVAIERGILGNGECGEKAQLIMWKNFVEEVTES
VRKCGRTRRVVSALSTGQGEDSLEIPEIALFRPDEAFETCGDWKQSLDTEKLLSILDVNSLVE
DAVSGKVMGKNKEYYGVGLWTLASFINHSCIPNARRLHVGDYVIVHASRDIKTGEEISFAYF
DVLSPLEKRKEMAESWGFCCGCSRCKFESVLYATNQEVREFEMGLERGVDAGNAVYMVEE
GMKRWKVKGKDKGLLRASYWGVYDEIYNSERLMKRWGRKIPTMEVVVDSVSDVVGSDER
LMKMAVEGMMKKHGGFSNIVEMEKIMKLGKGVYGKVVSKKKAMKTLLGIE

>AtS-ET;2

MSRLALNRYSRCFSRLKTLTTPLFFSSSAASNRDGDYQIGPPPIRVGLTESAGRAVFATRKIGA
GDLIHTAKPVVACPSLLKLDSVCYLCLKKLMGSAKFEDRGVSYCSQECQENSKGFLDVETRA
DWSSFDDYFLTKQWYTAILARIRINAFRIDLVGGSCGEDLLSLAAASVEGEGAVGHAVYMLP
SFYNHDCDPNAHIIWLHNADARLNTLRDVEEGEELRICYIDASMGYEARQTILSQGFGFLCNC
LRCQSTD

>AtS-ET;3

MDSVYKTDENFAADVAALLAPLPTPQLQEYFNKLITSRRCNGIEVKNNGTIGKGVYANSEFD
EDELILKDEILVGIQHSSNKVDCLVCSFCFRFIGSIEKQIGRKLYFKNLGVSGCCDDDSSEEDEC
VKYNGNEEQCGGSSSSHNTLPEGVVSSLMNGEMALPHTDKFPLPSPLSCPGGCQEAFYCSES
CAAADWESSHSLLCTGERSESISREALGEFIKHANDTNDIFLLAAKAIAFTILRYRKLKAEHVD
KKAKQSEPKQSLLLEAWKPVSIGYKRRWWDCIALPDDVDPTDEGAFRMQIKNLACTSLELL
KIAIFDKECEALFSLEIYGNIIGMFELNNLDLVVASPVEDYFLYIDDLPDAEKEETEEITRPFLD
ALGDEYSDCCQGTAFFPLQSCMNHSCCPNAKAFKREEDRDGQAVIIALRRISKNEEVTISYIDE
ELPYKERQALLADYGFSCKCSKCLEDSSSI

>AtS-ET;4a

MINDGGAKPETLLRVAEIGGRGRSLVAAQSLRAGQVILRESPLLLYSAFPFLSSSVSPYCDHCF
RLLASSAHQKCQSCSLVSFCSPNCFASHTPWLCESLRRLHQSSSSAFSDQPSDRQVQARFLLS
AYNLAAASPSDFQILLSLQGSGSSNGDPSCSAGDSAAAGFLHSLLSSVCPSLPVSISPDLTAALL
SKDKVNAFGLMEPCSVSNEKRSVRAYGIYPKTSFFNHDCLPNACRFDYVDSASDGNTDIIIRM
IHDVPEGREVCLSYFPVNMNYSSRQKRLLEDYGFKCDCDRCKVEFSWSEGEEDENEIMEEME
DQDEQEEMEDSVDICVRRRIVLAL

>AtS-ET;4b

MADLQRFLQDRCLGVSNLPQKGRSLFTARDFRPGEVILSQKPYICVPNNTSSESRCDGCFKTN
NLKKCSACQVVWYCGSSCQKSEWKLHRDECKALTRLEKEKRKFVTPTIRLMVRLYIKRNLQ
NEKVLPITTTDNYSLVEALVSHMSEIDEKQMLLYAQMANLVNLILQFPSVDLREIAENFSKFS
CNAHSICDSELRPQGIGLFPLVSIINHSCSPNAVLVFEEQMAVVRAMDNISKDSEITISYIETAGS
TLTRQKSLKEQYLFHCQCARCSNFGKPHDIEESAILEGYRCANEKCTGFLLRDPEEKGFVCQK
CLLLRSKEEVKKLASDLKTVSEKAPTSPSAEDKQAAIELYKTIEKLQVKLYHSFSIPLMRTREK
LLKMLMDVEIWREALNYCRLIVPVYQRVYPATHPLIGLQFYTQGKLEWLLGETKEAVSSLIK
AFDILRISHGISTPFMKELSAKLEEARAEASYKQLALH

>GrS-ET;1
MSGAEEQMQQLRSKATELLLREEWRDSIQLYSELIDLCQSQISNTHQHSDPDHLSKLHKSLCV
AFSNRAEARSKLQHFTQALNDCDQALQIEATHFKTLICKGKILLCLNRYFNALDCFKAALFDP
QGNGNLDIVNGYLEKCKKLEFQSRTGSFDLSDWVLNGFRGKPLELAEYIGPVKVNRSEISGR
GLFATKNIDTGTLVLVTKAIAIERGILGGQDSAENAQLVMWKNFIDKVKEAVTRCQRTQLLID
MLSTGENEAGLEVPDMSIFRPEIEENGCSNDQKLDMDKILSILDVNSLVEEAVSAKVLGKNSD
FYGVGLWILVSFINHSCNPNARRLHVGDYVIVHASRDVKAGEEITFMYFDALSPLEKRVEMS
LSWGFNCRCSRCKFEEAVCSKQELREIEIGLEKGVDVGGAVYRLEEGMKRWAVRGKGKGYL
RASFWAAYAEVYGSDRSMKRWGRRIPAMETVVDSVVEVMGSDERLLKVVVEGLKKSGGV
VEFERAMKLGRGFYGKVIKKQALRTLLEIGINDKGY

>GrS-ET;2
MSPLAFVLTLSCYRRWPSRFSTPYSHNLLVLFSSTATTITPPNGNESPSSGPAPPPIRVALTEST
GRAVFATRRIGAGDTIHTAKPIVSHPSLSAITTVCYFCLKKINAVTASQSQGVYFCSENCKESS
KVFYDVEKKTDWLAFDDYCRTQGLKYPLLVKRLACMVISGAAPAGILDILQPANLTQGMIL
KMEEGFHLLRNALVKANIGDEHMSFLTKQWYTDVLARIRINAFRIELAAGVYEDLLSLASASI
EAEAAVGNAIYMLPSFYNHDCDPNTHIVWIENADAKLKALRDIDEGEELQICYIDASMSYDA
RESLLSQGFGFKCNCLRCMSGD

>GrS-ET;3
MELICPIDQRCSHQIAALLRPPSPPIVQEYFDQLISKRKCQGIKVKQNGDLGKGVFAETDFEEE
QLIFKDEMLVGVQHPSNKIDCLVCSYCFKFIGSIEKQIGRKLYLKTIGIGNSSHNGCQSNSSDD
EEDNHYVQNHHSSENGASSSSGSTIPLPMMAVESLMNGELALPYSNKFPLPSVVSCLGGCEE
AFYCSKSCAEADWESSHCLLCTGEKSESCSRKALLKFIQHANETNDIFLLAAKAISFTILRYRK
LKASHMSEQEKPASSILGTDLSLLLEAWKPISIGHKRRWWDCIALPDDIDASDEATFRREIQEL
AFTSLQLLKEAIFDKECELLFSLEIYGHIIGMFELNNLDLVVASPVEDYFLYIDDLPYPEKKEAE
KITQPYLDALGEDYSNCCQGTAFFPLQSCMNHSCCPNAKAFKREEDRDGQATIIALRPICNGE
EVTISYIDEDLSFEERQALLADYGFRCRCPRCLDEER

>GrS-ET;4a
MLTADSGSVLRLVEIEGRGRALVASQPLKAGQIVLQDSPIVVYSAFPLVKPQSSASYCDNCFRI
LSSSANVVPCPLCSHHLFCGPNCLTAATASSHSPWVCQALSRLRDCPSLFSQPLEQQVQARFLI
AAYNLALVSPSDFQVLLSLQGQYSPSDAPAAEFLHSLILSVCPPPSLPISIELTAALLAKDKLNA
FGLMEPISLQQDGERSVRAYGIYPKASFFNHDCLPNACRFDYLDSAPAQNTDMIVRMIHDVP
VGREICLSYFPVNLNYSARQKRLTEDYGFTCNCDRCKVEANWSDNEADVIDDNGTVEENED
EEIMEEDNDEQMVASEGDEVGEADFPHAYFFVRYMCNRENCWGTLAPLPPSNDVQSKVLEC
NVCGNHKSEEDMC

>GrS-ET;4b
MEELQASLQPRGLTLSTFPDKGRSLLAARDFYPGEVIISQDPYVCVPNNSLTESRCDGCFSKSN
LKKCSACHVVWYCGSSCQKLEWKLHRFECQLLAKLDKERRKSVTPTIRMIVKLYLRRKLQN
ENVIPVTAMDNYNLVEALVSPILFSLKLEKDMSDIDEKQLLLYAQMANLVNLILQLPNIDIKEI
AENFSKFACNAHTICDSELRSLGTGLYPVISIINHSCLPNAVLVFEGRLAVVRAVQHIPKDSEV
SISYVETAASTITRQKTLKEQYLFTCTCVRCNKLGQYDDIQESAILEGYRCRDNGCSGFLLRES
DEKGFVCQQCGLIRNKEEIRKIASDIKALSDKALKCSSSGNLQEAIVLYKNIEKLQKEVCHPFSI
ILMRTREKLLEILMQLEEWKEALAICRLTIPVYERVYPGFHPLLGLQYYSCGKLEWLLGETDD
AIKSFTKAVDILRITHGTSTPFMKELLMKLEEARAEASFKLSSREDSYITL

>OsS-ET;1

MATPGLDDDSLQQLRSRATQLLLKENWTEYIAVCSLIIEAFDAAAACKDRRVLCSTLAHRAD
ARARLGDAPGALADCDAALAADPAHPGALLSKGAVLRGLGRYSRAAECFRAALAVSGTDE
VREMVEQCKRLDAQARSGAVDLSEWVLAGFSGKCPDLAEHVGAVEVRRSAHGGRGVFAV
KNIEAGANLVISKAVAIGRGVIPDAADSGEKMVVWKDLVDKVLDAAEKCPRTASLIYTLSTG
EEPEDELPIPDMAHFKQETEELDDGTAMAPKASLDVDKILKVLDVNCLTEDAAPSANLLGSN
GVVNCGVGLWILPAFINHSCHPNARRTHVGDHAIVHASRDIKAGEEITFAYFDVLTPASKRRE
AARAWGLECQCDRCRFEASDAIVGQELTKLENELVNGRGGDMGALVVRLEERMRKSMVKE
RRKAFLRASFWSAYSALFDSDKLVRKWGRRVPGEAAVAESVAGAIGGNESVLRAMLRGAD
NGNGCGNRLEVEDKVVRIGRATYGRVVKRQAMRALFRLTLDADSNKSL

>OsS-ET;2

MFHHLRRRLLCTAAAPPIRVALTESSGRGVFATRPISAGEVLHSAQPLVSHPSPPLIHEVCYSC
LRRKSGSGGGSSGSCYFCSDACREHAKGFHGVEKKADWSLFDDHCSSRGLKYPYMAKRLAC
MVISGAVSADCLDILQPARLHQGTLTEMEEEFALLDSTFRKAGFQEEITTFLTKEWYINVLARI
RINAFRIELVASSYENLLSSAVASVSCDAAVGNAVYMLPSFYNHDCDPNTHIVWLASADARL
KALRNIEEGEELRICYIDASMDVDARQRILAEGFGFECRCQRCLSGD

>OsS-ET;3

MFISTPLPQVRDKVSRTKPPKPHGGGGERRRKKQPQEAAARAGGGMGGSSASPCDLDREFAP
QIAQLLATPPLQPAQEYYNGLIQSRKHDGIRVNFSSKHGKGVCANKEFAEGDLILKDQILVGA
QHSLNKIDCAVCSYCFRFIGSIEFQIGRRLYWQSVGSSSDCTNRRHCHESDLGSSASSSGATKE
NSSTLPEEVLGSLITGDMSLPFTDHFSLPQVVPCRGCEEERYCSQSCADSDWETYHSLLCTGS
KTEPSQRSALQKFIEHANGSNDIFLVAAKAITFTLLRYKKLKTQPEFQNNTDESNFSLLMEAW
KPLSMGYKKRWWDSVALPEDVDSCDEDTFRQQIRDLALTSLQLLKDAIFDSECAPLFSLDVY
GHLIGMFELNNLGLVVPSPVEDYFIHIDDLPDDEKEEAEKVTRPFLDALGEDYAAPCEGTAFF
PLQSCMNHSCCPNAKAYKRDEDTDGNAVIIALEPIKKDDEITISYIDEDVSYEERQAELADYG
FICTCPRCQEEKPN

>OsS-ET;4a

MAGDALRAADLPGRGRGLLAARSIREGEVILTEQPLLLYPASLASLPSFCSACFRSLSAAASPC
PSCRAAGFCSPSCAAASHPRLLCTALSGGGGNGNLASAAEPHQEPLLFLLSAYSLPEPSLRVLL
SLSSAATPPPSDQDPGSLHAMVAALVPPQMLPPGFSPDLTAALLSKDRTNSFSIMEPYRPEVPQ
PLRKARAYAVYPRASLLNHDCLPNACHFDYADRPGPGNTDIVVRALHDITEGREVCLSYFAA
NWQYKDRQQRLLEDYGFRCECERCQVESKWKQDDDSDGGDGDDTMEEEEEDGNGGEGGD
DGMEQEEGDGGSDSDDDFPHSYFFVRYLCNHGECYGMLAPLPPLPNGEPSHVFECNVCGNL
KNEDEIDAPDGGDSSMAD

>OsS-ET;4b

MASWEEQLRDELAGRDLAVASVPGKGRGLFAARSFFPGEVVISQEPYASTPNKISVGSNCDN
CFASRNLRKCSVCRVAWYCGSACQREEWKLHQLECRAIAALTEDRKKMLTPTIRLMVRLVL
RRKLQDDKAIPSSGTDNYNLVDALESHISEVDKNQLVLYAQMANLVQLILPSFELDLKEITHT
FSKFACNAHTICDPELRPLGTGLYPVLSIINHSCVPNAVLIFEGRTAYVRALQPISKNEEVSISYI
ETAATTMKRQDDLKHYYFTCTCPRCVKDSEEDALLEGYRCNDQKCDGFLLPNAGNKGYTC
QKCSTSRDGEELQKMASDVLLLSDKVSSLVSSGNNSEVGSMYKTIEELERKLYHPLSITLLHT
RETLLKIYMELQDWQTALMYCRLTIPVYERIYPPFHPMIGLQFYTCGKLEWLLEYTEDALMS
LTRAADILRITHGTKSEFMKELLGKLEEVRAEASFRLSAGDEQ
