# Supplementary material for: Identification of SET Domain-Containing Proteins in Gossypium raimondii and Their Response to High Temperature Stress
Source: Sci Rep. 2016 Sep 7;6:32729. doi: 10.1038/srep32729 (PMC5013442; doi:10.1038/srep32729)
Supplement: Supplementary Table S4 [file srep32729-s5.pdf]

Supplementary Table S4

Identification of SET Domain-Containing Proteins in *Gossypium raimondii* and Their Response to High Temperature Stress

Yong Huang[1], Yijia Mo[1], Pengyun Chen[1], Xiaoling Yuan[1], Funing Meng[2], Shengwei Zhu[2, *], Zhi Liu[1, *]

[1] College of Bioscience and Biotechnology, Hunan Agricultural University, Changsha 410128, P. R. China

[2] Key laboratory of Plant Molecular Physiology, Institute of Botany, Chinese Academy of Sciences, Beijing 100093, P. R. China

[*]Corresponding author

Corresponding author:

Zhu S.

Key laboratory of Plant Molecular Physiology, Institute of Botany, Chinese Academy of Sciences, Beijing 100093, P. R. China

e-mail：zhusw@ibcas.ac.cn

Liu Z.

College of Bioscience and Biotechnology, Hunan Agricultural University, Changsha 410128, P. R. China

e-mail: tigerzhiliu@gmail.com

Supplementary Table S4 Blastn and Blastp results between homologous genes or proteins pairs.

Homologs	CDS			Protein		
	Identity %	Coverage %	E-value	Identity %	Coverage %	E-value
GrKMT1A;1a/1b	69	71	4E-169	58	83	0
GrKMT1A;3a/3b	82	98	0	67	99	0
GrKMT1A;3a/3c	46	90	1.00E-151	46	80	1.00E-151
GrKMT1A;3b/3c	46	77	2.00E-154	48	77	2.00E-15
GrKMT1A;4a/4b	70	67	8.00E-157	52	97	0
GrKMT1A;4a/4c	70	70	2.00E-165	52	97	0
GrKMT1A;4a/4d	68	71	2.00E-133	57	91	0
GrKMT1A;4b/4c	80	100	0	76	100	0

GrKMT1A;4b/4d	78	100	0	72	100	0
GrKMT1A;4c/4d	80	100	0	76	100	0
GrKMT1B;2a/2b	83	99	0	72	99	0
GrKMT1B;3b/3a	97	100	1.00E-135	97	100	8.00E-61
GrKMT1B;3b/3c	94	99	0	91	99	0
GrKMT1B; 3a/3c	96	100	3.00E-130	93	100	2.00E-58
GrKMT1B;3a/3d	97	85	1.00E-114	95	94	2.00E-56
GrKMT1B;3b/3d	97	56	0	66	95	2.00E-86
GrKMT1B;3c/3d	92	56	2.00E-168	86	76	2.00E-76
GrKMT2;3a/3b	71	76	0	53	100	0
GrKMT2;3a/3c	71	77	0	54	100	0
GrKMT2;3b/3c	87	100	0	84	100	0

GrKMT3;1a/1b	97	99	0	66	100	0
GrKMT6A;1a/1b	88	97	0	87	97	0
GrRBCMT;9a/9b	91	97	0	91	94	0
GrRBCMT;1a/2b	100	2	0.54	50	3	0.21
Note: Homologs whose covering of full-length-CDS sequence and identity of encoding amino acid between/among shorter and longer sequences >70 % are regarded as duplicated genes (Yang et al., 2008)						
